# Identification of anoikis-related molecular patterns and the novel risk model to predict prognosis, tumor microenvironment infiltration and immunotherapy response in bladder cancer

**DOI:** 10.3389/fimmu.2024.1491808

**Published:** 2024-11-27

**Authors:** Luochen Zhu, Feng Xiao, Yi Hou, Shenjun Huang, Yanyan Xu, Xiaohong Guo, Xinwei Dong, Chunlu Xu, Xiaolei Zhang, Haijuan Gu

**Affiliations:** ^1^ Department of Pharmacy, Nantong Tumor Hospital (Tumor Hospital Affiliated to Nantong University), Nantong, China; ^2^ Department of Pathology, Affiliated Nantong Hospital 3 of Nantong University (Nantong Third People’s Hospital), Nantong, China; ^3^ Department of Pharmacy, People’s Hospital of Zhongjiang, Deyang, China; ^4^ Department of Oncology, Nantong Tumor Hospital (Tumor Hospital Affiliated to Nantong University), Nantong, China; ^5^ Department of Andrology, Nanjing Drum Tower Hospital, The Affiliated Hospital of Nanjing University Medical School, Nanjing, China; ^6^ Department of Urology, The First Affiliated Hospital of Nanjing Medical University, Nanjing, China

**Keywords:** bladder cancer, anoikis, prognosis, tumor microenvironment, PLOD1

## Abstract

**Background:**

Anoikis, a unique form of cell death, serves as a vital part of the organism's defense by preventing shedding cells from re-attaching to the incorrect positions, and plays pivotal role in cancer metastasis. Nonetheless, the specific mechanisms among anoikis, the clinical prognosis and tumor microenvironment (TME) of bladder cancer (BLCA) are insufficiently understood.

**Method:**

BLCA patients were classified into different anoikis subtypes based on the expression of candidate anoikis-related genes (ARGs), and differences in the clinicopathological features, TME, immune cell infiltration, and immune checkpoints between two anoikis subtypes were analyzed. Next, patients in the TCGA cohort were randomized into the train and test groups in a 1:1 ratio. Subsequently, the anoikis-related model was constructed to predict the prognosis via utilizing the univariate Cox, LASSO and multivariate Cox analyses, and validated internally and externally. Moreover, the relationships between the risk score and clinicopathologic features, immune cell infiltration, immunotherapy response, and antitumor drug sensitivity were also analyzed. In addition, representative genes were evaluated using immunohistochemistry in clinical specimens, and in BLCA cell lines, functional experiments were performed to determine the biological behavior of hub gene PLOD1.

**Result:**

Two definite anoikis subgroups were identified. Compared to ARGcluster A, patients assigned to ARGcluster B were characterized by an immunosuppressive microenvironment and worse prognosis. Then, the anoikis-related model, including PLOD1, EHBP1, and CSPG4, was constructed, and BLCA patients in the low-risk group were characterized by a better prognosis. Next, the accurate nomogram was built to improve the clinical applicability by combining the age, tumor stage and risk Score. Moreover, immune infiltration and clinical features differed significantly between high- and low-risk groups. We also found that the low-risk group exhibited a lower tumor immune dysfunction and exclusion score, a higher immunophenoscore (IPS), had more sensitivity to immunotherapy. Eventually, the expression levels of three genes were verified by our experiment, and knockdown of PLOD1 could inhibit invasion and migration abilities in BLCA cell lines.

**Conclusion:**

These results demonstrated a new direction in precision therapy for BLCA, and indicated that the ARGs might be helpful to in predicting prognosis and as therapeutic targets in BLCA.

## Introduction

Statistics show that bladder cancer (BLCA) has been increasing in incidence each year, and it ranks as one of the ten most common tumors. In men, BLCA is more prevalent due to hormones, tobacco use, and other factors, ranking sixth among all cancers (1–4). It is estimated that 45% to 50% of patients with non-muscle invasive bladder cancer (NMIBC) will suffer a recurrence, and 6% to 40% will progress to more advanced stages of the disease (5, 6). In approximately 50% of MIBC patients, disseminated micro-metastases lead to distant disease, even after radical cystectomy and pelvic lymph node dissection. Advanced BLCA patients are often treated with neoadjuvant chemotherapy and cisplatin-based chemotherapy, as well as surgery (7). Recently, immune checkpoint therapy has been shown to be a promising treatment for BLCA, targeting PD-1, PD-L1, and CTLA4. For patients with metastatic or unresectable BLCA, PD-1 inhibitors and PD-L1 inhibitors have shown therapeutic benefit as a second-line treatment (8–10). Nevertheless, like other cancers, this treatment may only be beneficial to a small number of patients (11, 12). As a result, novel medical tools and treatment modalities that can treat BLCA patients are urgently needed.

Anoikis, a specific form of apoptosis, plays a crucial role in the organism’s defense by preventing shed cells from re-attaching to incorrect locations (13, 14). As the cells lose contact with the extracellular matrix (ECM), it is triggered (15, 16). Initially discovered in endothelial and epithelial cells, anoikis is associated with tissue homeostasis and development (17). Currently, studies have shown that anti-anoikis mechanisms play a pivotal role in cancer development (18, 19). A number of molecular pathways and tools have recently been identified that regulate anoikis resistance and some downstream molecules, such as PI3K/Akt, ERK1/2, MAPK, and Bcl-2 family, which have been considered to serve crucial roles in anti-apoptotic and pro-survival (16, 20–24). However, the molecular mechanisms and cellular features of anoikis in BLCA are still unknown.

In our study, the association between anoikis phenotypes and prognosis, and tumor microenvironment (TME) were comprehensively evaluated. Initially, the gene mutations and expression levels of the candidate genes were analyzed, leading to the identification of two distinct anoikis subgroups. Then, three gene subtypes were classified according to differentially expressed genes (DEGs) between two distinct anoikis phenotypes. Moreover, the anoikis-related model was constructed, and we explored its relationship with the prognosis, immune cell infiltration, immunotherapy response, and antitumor drug sensitivity. Additionally, the expression levels of three genes (PLOD1, EHBP1, and CSPG4) were validated with immunohistochemistry (IHC). Last but not least, the role of the hub gene PLOD1 was investigated by further experimental verification. The results might provide novel insights into targeted therapy for BLCA patients.

## Materials and methods

### Data acquisition

Gene expression data, copy number variation (CNV) data, somatic mutation data, and clinicopathological data of BLCA patients were obtained from the TCGA (https://portal.gdc.cancer.gov), including 19 normal samples and 412 tumor samples. The GSE13507 were retrieved from the GEO (https://www.ncbi.nlm.nih.gov/geo/), including 165 tumor samples. Based on previously published literature, 434 anoikis-related genes (ARGs) were identified (25, 26) (**Supplementary Table S1**).

### Consensus clustering analysis of ARGs in BLCA

The consensus clustering analysis was performed in accordance with the ARG expression via the “ConsensusClusterPlus” R package, and different molecular subtypes were identified in BLCA samples (27, 28). Principal component analysis (PCA) was utilized to visualize the distribution of distinct subgroups. Then, the Kaplan-Meier (KM) method was utilized to compare the prognosis of distinct anoikis subgroups. Additionally, by utilizing the “gsva” R package, gene set variation analysis (GSVA) was performed to investigate the differences in biological processes.

### Analysis of the correlation between BLCA immune genome subtypes and molecular characteristics

To calculate the scores of immune cells infiltrating, single-sample gene set enrichment analysis (ssGSEA) was utilized. The differences in HLA genes and immune checkpoints between distinct anoikis subgroups were also evaluated. Moreover, to investigate the differences in TME among anoikis subgroups, the stromal score, immune score, ESTIMATE score and tumor purity were analyzed via the ESTIMATE algorithm.

### Gene consensus clustering analysis of anoikis phenotype-associated DEGs

To identify anoikis phenotype-associated DEGs, “limma” R package was utilized with adjusted *P* < 0.05 and |log2(FC)| ≥ 1. Then, we carried out Gene Ontology (GO) and Kyoto Encyclopedia of Genes and Genomes (KEGG) analyses in accordance with the DEGs. Next, the univariate Cox was employed, and the candidate genes were selected for the clustering analysis. The gene subgroups were eventually constructed and the heatmap was mapped by combining the anoikis subgroups, gene clusters and clinical characters of BLCA patients.

### Identification of the anoikis-related model and the nomogram

The BLCA patients in the TCGA were randomly separated into two groups, including the training set (n = 202) and testing set (n = 202). Then, the DEGs among the gene clusters were subjected to the univariate cox analysis, and *P* value of 0.05 was chosen as a cutoff. To avoid overfitting, the LASSO regression analysis was utilized via the “glmnet” R package. Moreover, multivariate Cox analysis was utilized to select the candidate genes to establish the anoikis-related model. The riskScore formula was as follows: riskScore = Σ (Expi * coefi) (EXpi: gene expression level, coefi: coefficients). In accordance with the median risk score obtained from the risk score, the patients were divided into high-risk groups and low-risk groups. Next, the survival curve and the ROC curve were utilized to validate the prognostic and accuracy of the anoikis-related model in BLCA. According to the risk score and predictive characteristics in BLCA, the “rms” R package was utilized to perform the nomogram that could evaluate the overall survival (OS) of BLCA patients. Calibration curves were made to show the predictive power of our nomogram, and decision curve analysis (DCA) was utilized to assess the clinical net benefit of BLCA (29, 30).

### Analysis of the immune landscape in BLCA

In BLCA, to further explore the association with immune cells and the anoikis-related model, the spearman correlation analysis was utilized via seven methods, including CIBERSORT, CIBERSORT-ABS, EPIC, MCPCOUNTER, QUANTISEQ, and XCELL. Moreover, the ssGSEA was utilized to evaluate the activity of immune-associated pathways between two risk groups. Additionally, the relationship between the expression of eight immune checkpoints and anoikis-related model was analyzed.

### Immunotherapy response and drug susceptibility analysis

Immunophenoscore (IPS) and Tumor Immune Dysfunction and Exclusion (TIDE) were utilized to predict immunotherapy response (31). IPS can calculate z-scores according to 4 immunogenicity-associated cell types, and its data was downloaded from the TCIA database (http://tcia.at/) (32). An algorithm called TIDE was utilized to investigate distinct mechanisms of tumor immune escape (33). In addition, the IMvigor210 cohort was further utilized to assess the predictive ability of our model for immunotherapy response (34, 35). To investigate the differences in sensitivity predictions for common chemotherapeutic agents between low- and high-risk groups in bladder cancer (BLCA), the half-inhibitory concentration values of the drugs were calculated using the ‘pRRophetic’ package (36, 37).

### Cell lines and culture

The human urothelial carcinoma cells lines (T24, 5637, UM-UC-3 and J82) and human normal uroepithelial cell lines (SV-HUC1) were all purchased from ATCC (Virginia, USA) and regularly screed for mycoplasma in the laboratory. All of these cell lines were cultured in RPMI medium (Gibco, Grand Island, USA) containing 10% fetal bovine serum (FBS, Gibco) and added antibiotics penicillin and streptomycin at 1% final concentrations at 37°C in a 5% CO2 incubator.

### SiRNAs and transfections

SiRNAs targeting PLOD1 and the control siRNA were synthesized by ObiO Technology (Shanghai, China), the sequences were listed in the **Supplementary Table S2**. The siRNA transfection was performed using Lipofectamine 2000 (Invitrogen, USA) according to the manufacturer’s instructions.

### Cell Counting Kit-8 (CCK-8) Assay

For the cell viability assay, CCK-8 assays (Beyotime, Shanghai, China) were performed. Cells with respective treatments were seeded in 96-well plates at 4×103 cells/well cell concentration. After cell culture for 0 h, 24 h, 48 h, and 72 h, respectively, 10 μL CCK-8 solution was added to each well and incubated at 37°C for 2 h. Optical density (OD) at 450 nm was measured for each well using a microplate reader (Dojindo, Kumamoto, Japan).

### Migration assay

To test the human urothelial carcinoma cell line (5637) migration, 5637 (5 × 10^4^ were resuspended in serum-free medium and added to the upper chambers of transwell plates (8-μm pore size). The medium containing 10% serum was added to the lower chambers. After 48 h incubation at 37°C in a humidified chamber with 5% CO2 membranes were rinsed by dH2O, and cells remaining in the upper chamber were removed by a cotton swab. Membranes were fixed and then stained with 0.5% crystal violet. Cells that migrated to the lower chamber were counted.

### Wound healing assay

Human urothelial carcinoma cells with respective treatments were cultured in six-well plates at 37°C. A wound was created by scratching the cell monolayer using the fine end of a 1 mL pipette tip. Images of migrated cells were captured under microscopy at indicated time points (0 h after wound scratching and 48 h after wound scratching). The % of wound healing was calculated using the formula below: wound healing (%) = (wound width at 0 h – wound width at 48 h)/wound width at 0 h × 100%.

### Colony formation assays

The treated human urothelial carcinoma cells were seeded into 6 well plates with 500 cells per well for colony formation. After two weeks, the formed cell colonies were fixed with 4% paraformaldehyde for 10 minutes and stained with 0.5% (W/W) crystal violet (diluted in phosphate buffered brine, PBS) for 30 minutes.

### Clinical specimens

BLCA and adjacent tissues were acquired from patients who underwent radical surgery from The First Affiliated Hospital of Nanjing Medical University, and informed consent was signed from all patients included in this study before the surgery. The study was permitted by the Ethics Committee of The First Affiliated Hospital of Nanjing Medical University.

### Immunohistochemistry

In the immunostaining of tissues, the primary antibodies anti-PLOD1(#38770, 1:100) and anti-EHBP1(#93614, 1:100) were purchased from Novus, anti-CSPG4(#43916T, 1:50) was purchased from Cell Signaling Technology. The specific experimental procedures refer to the previous literature (38).

### Immunoblotting

The cell lysates were prepared and the same amounts of proteins were subjected to SDS-PAGE and transferred to polyvinylidene fluoride membrane by electroblotting. After blocking, the membrane was incubated with the corresponding antibody. The second antibody was horseradish peroxidase (HRP) combined with goat IgG against IgG (Santa Cruz Biotechnology). Imprinting is formed with an ECL substrate (Pierce) and exposed to X-ray film for analysis by Image.lab3.0 software.

### Statistical analysis

In our study, all statistical analyses were employed with the R software (version 4.2.1) and GraphPad Prism software (9.1.0). Statistical significance was set at *P* < 0.05.

## Results

### Identification of candidate anoikis-related genes

**Figure 1:**
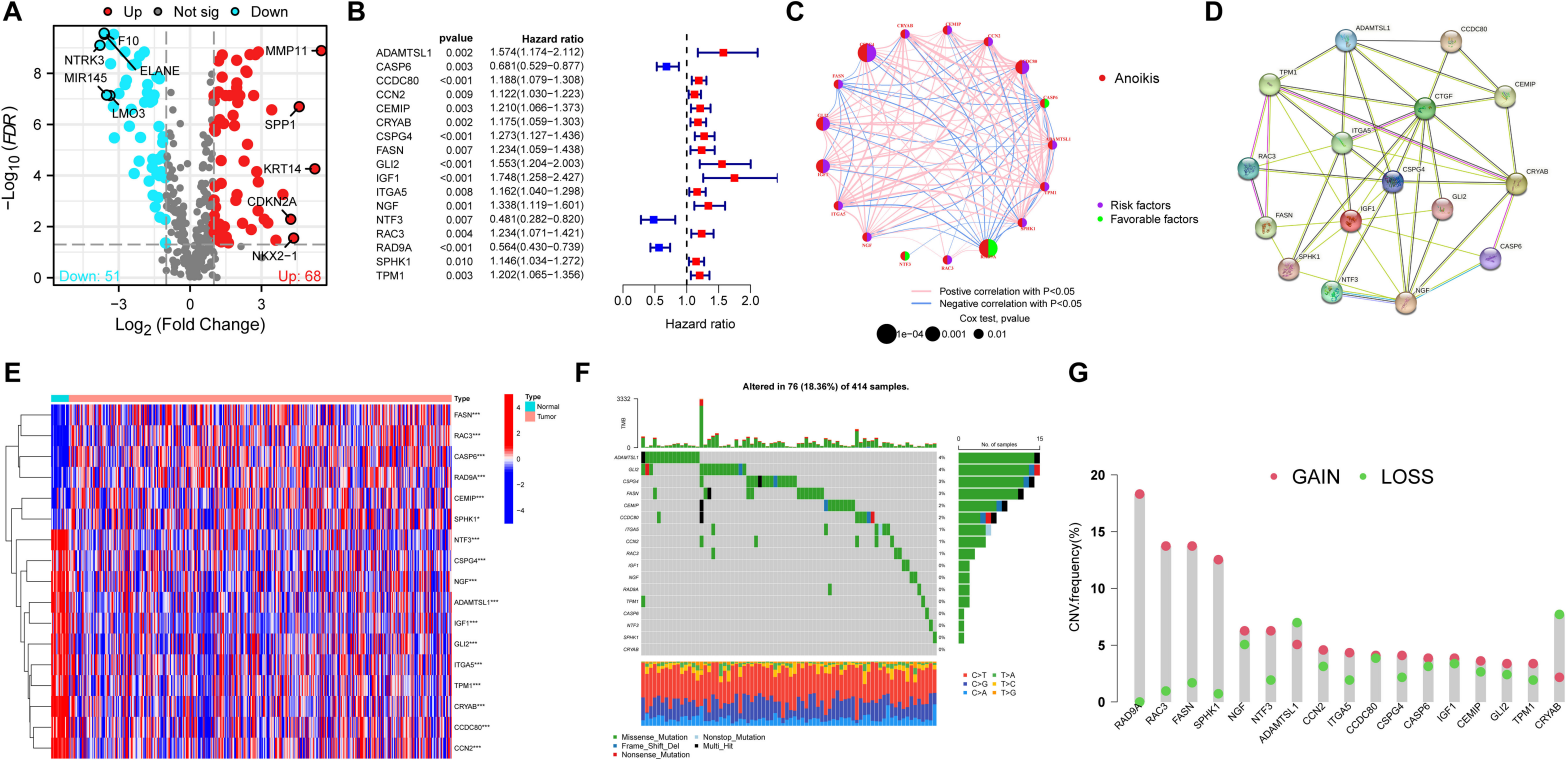
Characteristics of candidate ARGs in BLCA. **(A)** The volcano plot of differentially expressed ARGs in normal and tumor samples in the TCGA cohort. **(B)** The forest plot showed 17 of 119 differentially expressed ARGs concerned with OS (*P* < 0.01). **(C)** The network plot of interactions and the prognosis of BLCA among 17 ARGs. **(D)** The PPI network uncovered a close linkage among most candidate ARGs. **(E)** The heatmap of 17 candidate ARGs. **(F)** The mutation prevalence of 17 ARGs in BLCA. **(G)** The frequency of CNVs of 17 candidate ARGs in BLCA. ARGs, anoikis-related genes; BLCA, Bladder cancer; OS, overall survival. *P<0.05; ***P<0.001.

In our study, by utilizing “limma” R package, differences in gene expression were analyzed. 51 ARGs with down-regulated, and 68 ARGs with up-regulated were obtained with |log2FC| > 1 (**Figure 1A**, **Supplementary Table S3**). Then, univariate Cox analysis indicated that 17 of 119 differentially expressed ARGs were concerned to OS (*P* < 0.01) (**Figure 1B**). Next, the network plots of 17 ARGs interactions and the prognostic value of BLCA patients were presented (**Figure 1C**). Meanwhile, PPI network uncovered a close linkage among most candidate ARGs (**Figure 1D**). By utilizing “pheatmap” R package, the heatmap of 17 ARGs were plotted (**Figure 1E**). Moreover, mutation data demonstrated that 76 (18.36%) of BLCA samples had ARGs mutations, of which ADAMTSL1 (4%), and GLI2 (4%) had the highest mutation frequency (**Figure 1F**). The frequency of copy number variations (CNVs) in 17 candidate apoptotic-related genes (ARGs) was also investigated. Notably, RAD9A exhibited the most significant increase in CNVs, while CRYAB showed the most substantial CNV deletion (**Figure 1G**).

### Identification of anoikis subgroups in BLCA

**Figure 2:**
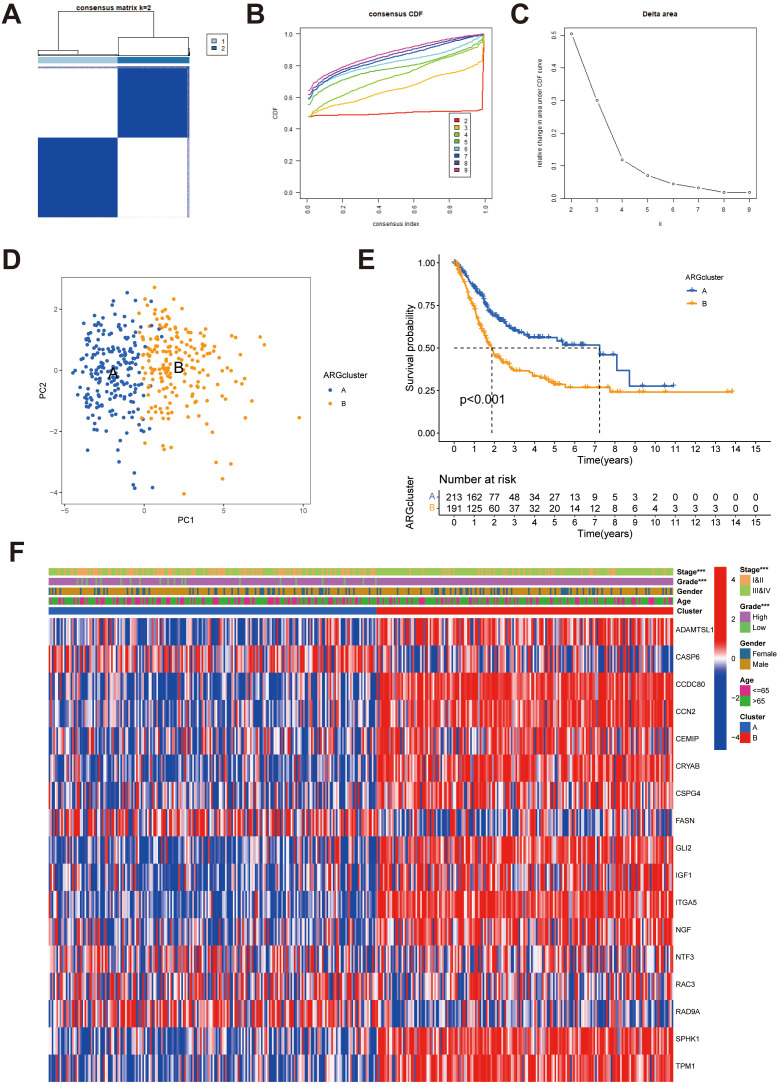
Identification of potential anoikis subgroups in BLCA. **(A)** Two anoikis subgroups (k = 2) and their correlation area are defined by consensus matrix heatmap. **(B)** The consensus clustering CDF. **(C)** The analysis of the variation in area under the CDF region. **(D)** PCA indicated the different distributions between the potential anoikis subgroups. **(E)** Survival analyses for the ARGcluster A (n = 213) and ARGcluster B (n = 191) cohorts. **(F)** Differences in clinical characteristics and expression levels of candidate ARGs between ARGcluster A and ARGcluster **(B)** BLCA, Bladder cancer; CDF, cumulative distribution function; ARGs, anoikis-related genes. ***P < 0.001.

To fully explore the expression pattern of 17 ARGs in BLCA, unsupervised clustering analysis was utilized. The results indicated that K = 2 was determined to be the best cluster, and 404 BLCA patients fell into ARGcluster A (n = 213) and ARGcluster B (n = 191) (**Figures 2A–C**; **Supplementary Table S4**). PCA analysis manifested the remarkable differences in the anoikis transcription profiles between ARGcluster A and ARGcluster B (**Figure 2D**). The differences in prognosis between two subtypes were identified by the OS analysis (*P* < 0.001) (**Figure 2E**). The heatmap indicated significant differences in clinicopathological characteristics, including tumor stage (*P* < 0.001) and grade (*P* < 0.001) between two subtypes (**Figure 2F**).

### Characteristics of the tumor microenvironment in anoikis subgroups

**Figure 3:**
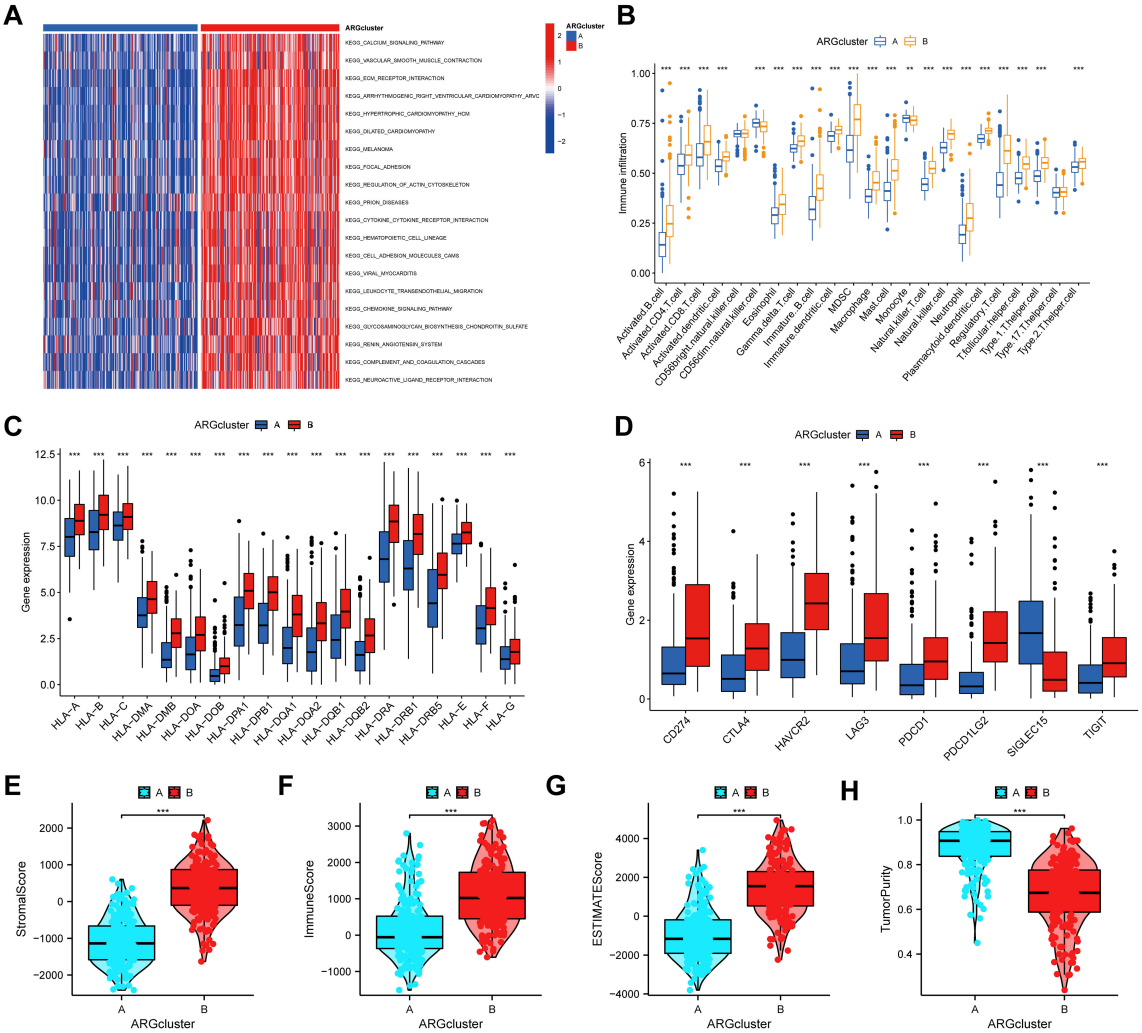
Correlations of the TME and two different anoikis subtypes. **(A)** GSVA of biological processes between two distinct anoikis subtypes. **(B)** The abundance of 23 infiltrating immune cells between two distinct anoikis subtypes. **(C)** The expression levels of HLA genes between two anoikis subgroups. **(D)** The expression levels of immune checkpoints between two anoikis subgroups. **(E-G)** The analyses of **(E)** stromal score, **(F)** immune score, **(G)** ESTIMATE score, and **(H)** tumor purity between two anoikis subgroups. TME, tumor microenvironment; HLA, Human Leukocyte Antigen. **P < 0.01; ***P < 0.001.

To explore the biological processes in the anoikis subgroups, GSVA analysis was conducted. The results indicated that ARG cluster A was significantly enriched in pathways related to calcium signaling, vascular smooth muscle contraction, ECM-receptor interaction, arrhythmogenic right ventricular cardiomyopathy, hypertrophic cardiomyopathy, dilated cardiomyopathy, melanoma, focal adhesion, regulation of the actin cytoskeleton, prion diseases, cytokine-receptor interaction, and hematopoietic cell lineage (**Figure 3A**).We further assessed the differences in immune cell infiltration between two anoikis subgroups, and the infiltration levels of most cells, such as activated CD4 T cell, activated dendritic cell, immature dendritic cell, macrophage, and type 1 T helper cell were higher in the ARGcluster B than those in the ARGcluster A. However, CD56 dim natural killer cells and monocyte had higher infiltration in ARGcluster B than those in ARGcluster A (**Figure 3B**). Moreover, the expression levels of HLA genes were found to differ between two anoikis subgroups. Surprisingly, all of the HLA genes showed higher expression in ARGcluster B than in ARGcluster A (**Figure 3C**). We also observed that except SIGLEC15, other vital immune checkpoints, such as TIGIT, PDCD1LG2, PDCD1, LAG3, HAVCR2, CTLA4, and CD274 were all lowly expressed in the ARGcluster A than in the ARGcluster B (**Figure 3D**). Regarding the TME score, patients in the ARGcluster B have a lower tumor purity, whereas patients in the ARGcluster A had a lower stromal score, immune score, and estimate score, suggesting that BLCA patients in the ARGcluster A had lower immune activity (**Figures 3E-H**).

### Identification of gene clusters based on anoikis phenotype-associated DEGs

**Figure 4:**
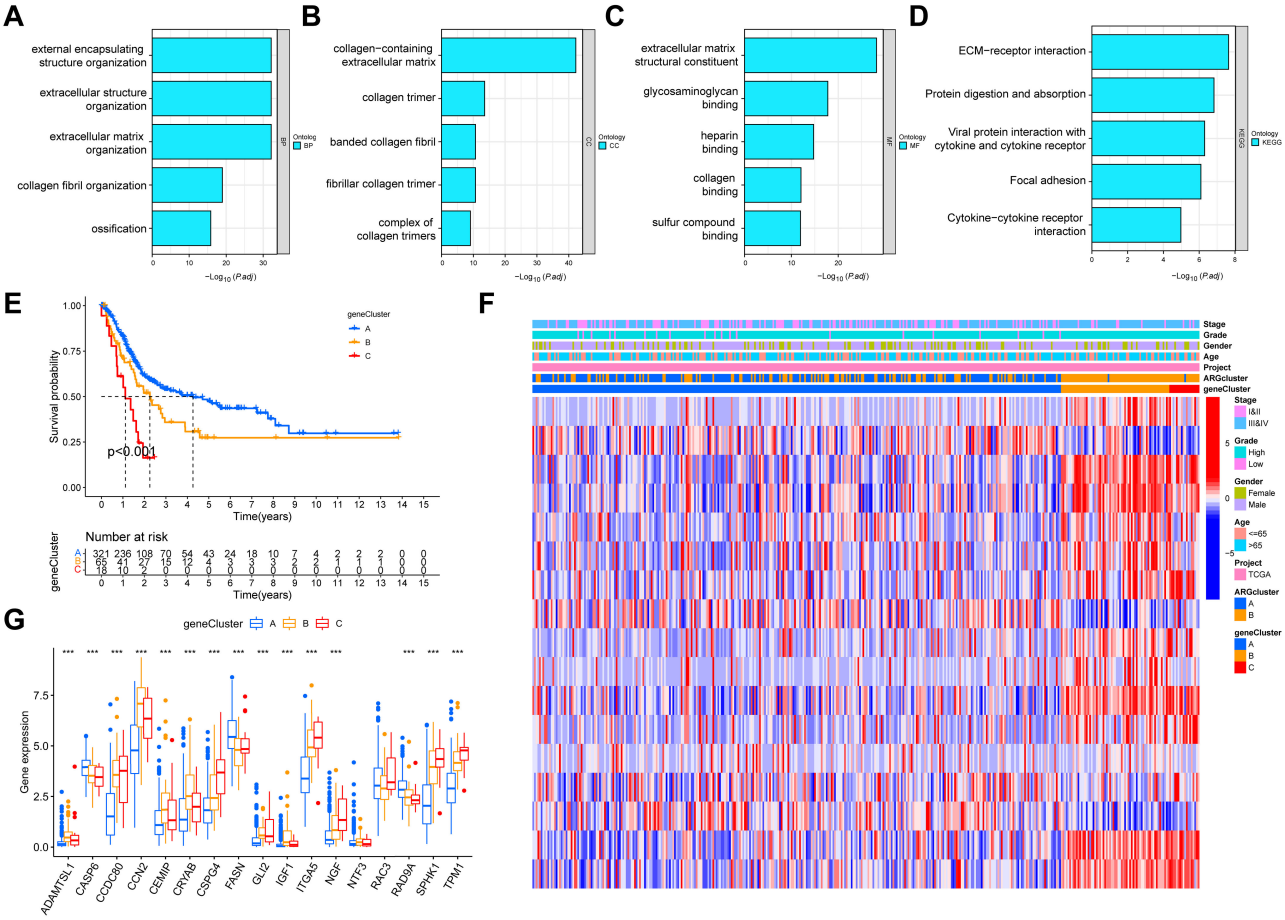
Identification of gene clusters in accordance with the anoikis phenotype-associated DEGs in BLCA. **(A-C)** GO enrichment analyses based on the anoikis phenotype-associated DEGs. **(D)** KEGG enrichment analyses based on the anoikis phenotype-associated DEGs. **(E)** KM survival curves for three gene subtypes in BLCA. **(F)** Heatmap indicating the association between geneCluster and clinicopathologic characteristics. **(G)** Expression levels of 17 candidate ARGs in three gene clusters. DEGs, differentially expressed genes; BLCA, Bladder cancer; GO, Gene Ontology; KEGG, Kyoto Encyclopedia of Genes and Genomes; KM, Kaplan-Meier. ***P < 0.001.

Although consensus clustering algorithm was utilized to identify two anoikis subgroups in BLCA, the potential biological behavior and genetic alterations of two ARG clusters remained clarified. Finally, 446 anoikis phenotype-associated DEGs were obtained (**Supplementary Table S5**). Go results demonstrated an association with external encapsulating structure organization, extracellular structure organization, collagen-containing extracellular matrix, collagen trimer, extracellular matrix structural constituent, glycosaminoglycan binding, etc (**Figures 4A-C**; **Supplementary Table S6**). KEGG showed an association with ECM-receptor interaction, protein digestion and absorption, viral protein interaction with cytokine and cytokine receptor, focal adhesion, cytokine-cytokine receptor interaction, etc (**Figure 4D**; **Supplementary Table S6**). Then, univariate Cox was employed to screen out 446 genes related to OS in BLCA, and 24 genes were eventually identified at *P* < 0.001 (**Supplementary Table S7**). According to the transcriptional levels of these 24 genes in BLCA, consensus clustering analysis was performed to classify BLCA patients into three gene subtypes (geneCluster A, geneCluster B and geneCluster C) (**Supplementary Figure S1**, **Supplementary Table S8**). Survival analysis indicated that patients in geneCluster C had the worst OS among three clusters, and patients in geneCluster A demonstrated a superior survival outcome (*P* < 0.001) (**Figure 4E**). Additionally, anoikis subgroups, gene clusters and clinical characters of BLCA patients were combined to map the heatmap, and the different expression patterns were found among three clusters (**Figure 4F**). Furthermore, most of the expression of 17 candidate ARGs were significantly differed among three gene clusters (**Figure 4G**).

### Construction and validation of anoikis-related model in BLCA

In our study, to further quantify the risk of each BLCA patient, an anoikis-related model was constructed based on gene cluster-related DEGs. First, 86 DEGs were identified among three gene subtypes (**Supplementary Table S9**). Then, the “caret package” in R was utilized to randomize 404 BLCA patients into the training set (n = 202) and the testing set (n = 202) at a ratio of 1:1 (**Supplementary Tables S10**, **S11**). Next, in the training set, univariate Cox, LASSO and multivariate Cox analyses were utilized to build an appropriate model in accordance with the 86 DEGs (**Supplementary Figures S2A-C**). Finally, three genes, including EHBP1, CSPG4, and PLOD1 were identified to construct the anoikis-related model, and the risk score of each BLCA patient was calculated based on the formula: Risk score = 0.2554*expression of EHBP1 expression + 0.2014*expression of CSPG4 + 0.4737*expression of PLOD1 (**Supplementary Figure S2C**). According to the Sankey diagram, there was a correlation between anoikis subgroups and gene clusters, as well as risk scores. Moreover, we found a significant difference in the risk score of anoikis subgroups. The previous analysis demonstrated the more prolonged OS in the ARGcluster A, and the model demonstrated the lower risk scores in the ARGcluster A, which further showed the excellent and reliable performance of anoikis-related model in BLCA (**Supplementary Figure S3**).

**Figure 5:**
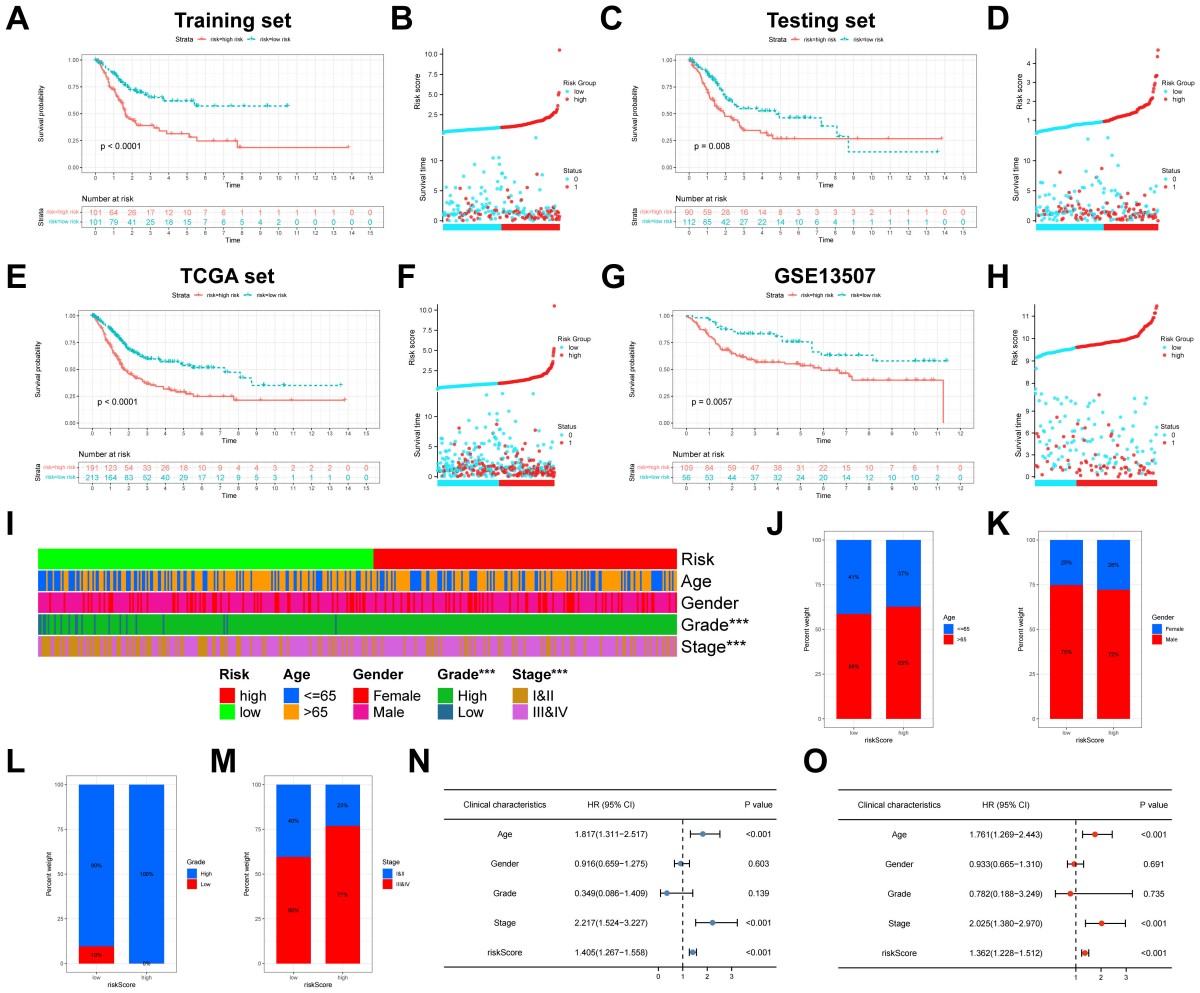
Construction and validation of anoikis-related model in BLCA. **(A)** Kaplan-Meier analysis of the training set. **(B)** The distribution of risk score and the survival status of BLCA patients in the training set. **(C)** Kaplan-Meier analysis of the testing set. **(D)** The distribution of risk score and the survival status of BLCA patients in the testing set. **(E)** Kaplan-Meier analysis of the TCGA set. **(F)** The distribution of risk score and the survival status of BLCA patients in the TCGA set. **(G)** Kaplan-Meier analysis of the GSE13507. **(H)** The distribution of risk score and the survival status of BLCA patients in the GSE13507. **(I)** The heatmap showed the relevance of anoikis-related model and clinicopathological characteristics. **(J-M)** The differences in age, gender, grade, and stage between the high- and low-risk groups. **(N)** The univariate Cox analyses of risk score and clinicopathological variables with OS. **(O)** The multivariate Cox analyses of risk score and clinicopathological variables with OS. BLCA, Bladder cancer; OS, overall survival. ***P < 0.001.

Next, in accordance with the median risk score, BLCA patients were divided into high- and low-risk groups. As shown in **Figure 5A**, KM survival analysis indicated that patients in the high-risk group had worse OS than those in the low-risk group (*P* < 0.0001). The distribution plot of the risk score uncovered that the high-risk group had worse survival status and shorter survival time (**Figure 5B**). The AUCs of 1-, 3-, and 5-years were 0.67, 0.70, and 0.67, respectively (**Supplementary Figure S4A**). To validate the anoikis-related model in BLCA, the testing set and the entire set (TCGA) were utilized internally and GSE13507 was utilized externally. Similar results were also obtained in our study, which further indicated that the anoikis-related model had the excellent power to predict prognosis of BLCA (**Figures 5C-H**; **Supplementary Figures S4B-D**).

**Figure 6:**
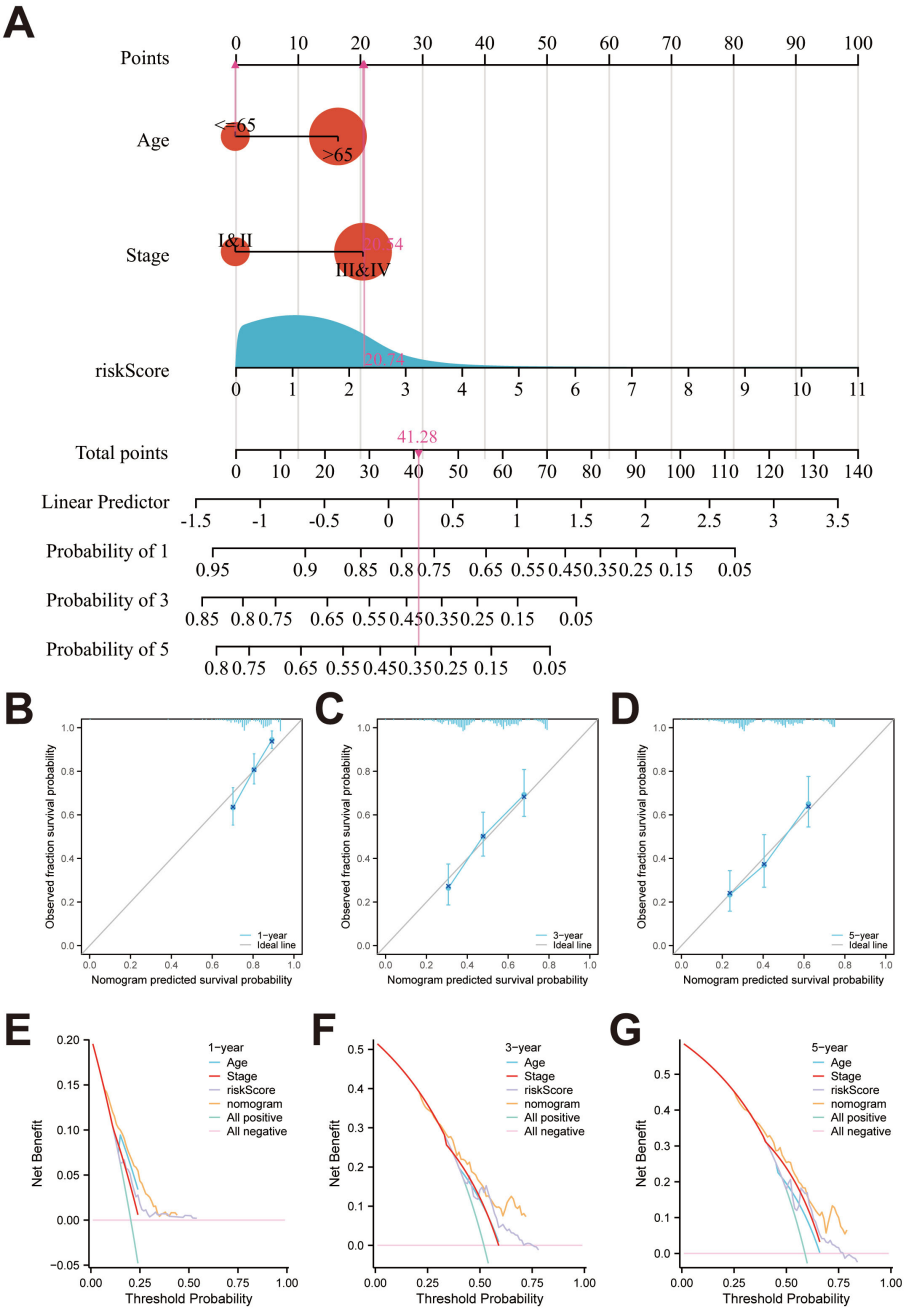
Identification of a nomogram for predicting OS in BLCA. **(A)** The nomogram for predicting 1-year, 3-year, and 5-year survival rates for BLCA patients. **(B-D)** Calibration curves of nomogram. **(E-G)** The DCA of the nomogram, risk score, age and stage.

Further, the heatmap depicted the relevance of anoikis-related model and clinicopathological characteristics, which suggested that grade and stage were significantly relevant to the model (both *P* < 0.001) (**Figure 5I**). We also found that the proportions of age > 65, female, high grade, and stage III and stage IV patients in the high-risk group were significantly higher than those of the low-risk group (**Figures 5J-M**). To prove the independence of risk score, Cox regression analysis was conducted in the entire TCGA set. As shown in **Figures 5N, O**, in accordance with the results of univariate (HR = 1.405; 95%CI = 1.267-1.558; *P* < 0.001) and multivariate (HR = 1.362; 95%CI = 1.228-1.512; *P* < 0.001) analyses, our risk score could be independent predictor in BLCA. In addition, to make the anoikis-related model easy to employ in clinic, the nomogram was created by integrated age, stage and the riskScore (**Figure 6A**). Surprisingly, the calibration plots exhibited great consistency between the predicted and actual 1-, 3-, and 5-year OS (**Figures 6B-D**). Subsequently, the clinical benefits of age, stage, riskScore and nomogram were assessed, and the DCA illustrated that the nomogram led to better benefit than other factors (**Figures 6E-G**).

### The anoikis-related model significantly associated with immune status and pathways

**Figure 7:**
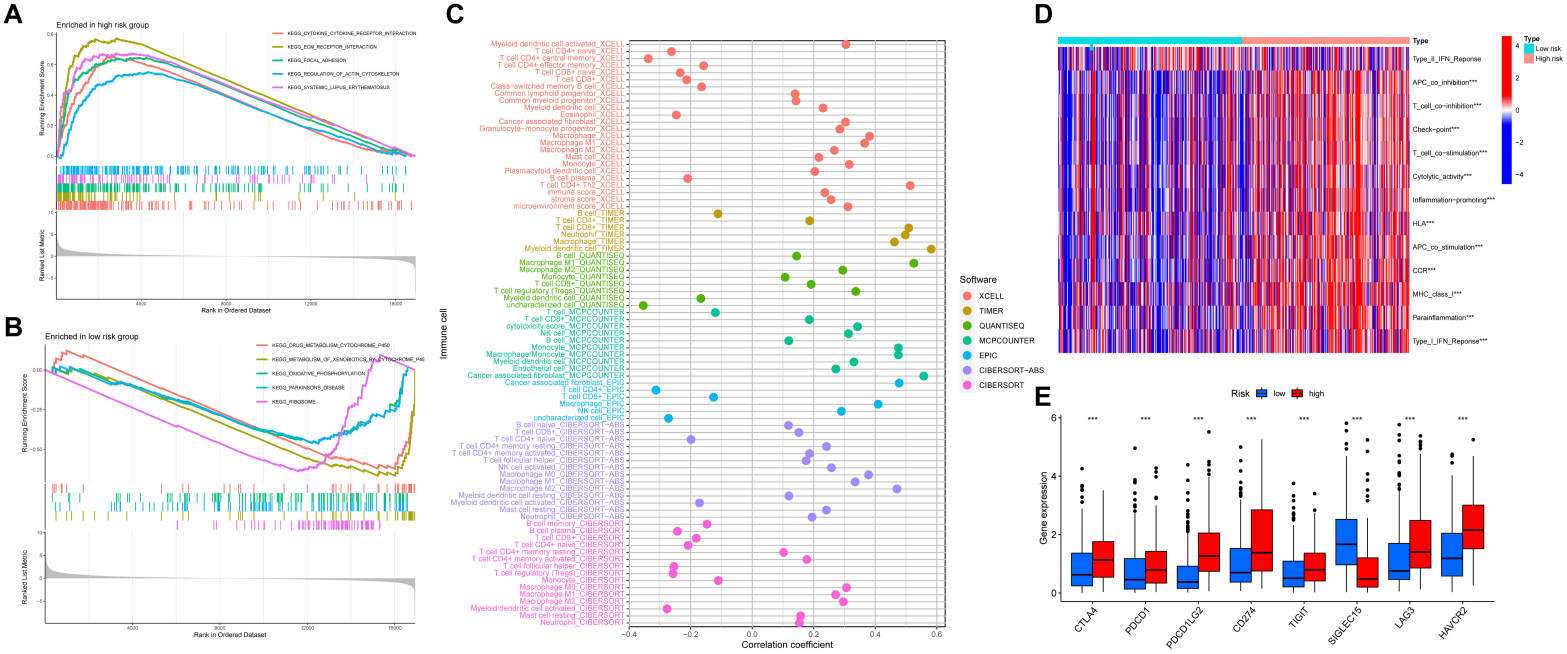
The relationship between the anoikis-related model and the immune status. **(A, B)** GSEA indicates the functional pathways in the high- and low-risk groups. **(C)** The Spearman correlation analysis of immune components and the risk score based on different algorithms. **(D)** The heatmap showing the association with the risk-scores and immune-related functions. **(E)** The expression levels of immune checkpoints between high- and low-risk groups. ***P<0.001.

The results of GSEA uncovered that the top five pathways were cytokine receptor interaction, ECM receptor interaction, focal adhesion, regulation of actin cytoskeleton, and systemic lupus erythematosus in the high-risk group, and drug metabolism cytochrome P450, metabolism of xenobiotics by cytochrome 450, oxidative phosphorylation, Parkinsons disease, and ribosome in the low-risk group (**Figures 7A, B**). To investigate the correlation between immune cell infiltration and risk score, CIBERSORT, CIBERSORT-ABS, EPIC, MCPCOUNTER, QUANTISEQ, TIMER, and XCELL were applied. For instance, using the CIBERSORT algorithm, we found that the risk score was negatively correlated with memory B cells, plasma B cells, CD8+ T cells, naive CD4+ T cells, activated myeloid dendritic cells, follicular helper T cells, regulatory T cells (Tregs), and monocytes. In contrast, it was positively correlated with resting and activated memory CD4+ T cells, M0, M1, and M2 macrophages, resting mast cells, and neutrophils (**Figure 7C**, **Supplementary Table S12**). Additionally, the relationship between 13 immune-related pathways and the risk score was explored by ssGSEA. We found that except the type II IFN response, other pathways, such as type I IFN response, parainflammation, inflammation promoting, cytolytic activity and so on were all improved in the high-risk group (**Figure 7D**). Furthermore, the expression of immune checkpoints was analyzed, and **Figure 7E** illustrated that CD274, HAVCR2, PDCD1LG2, TIGIT, PDCD1, LAG3, and CTLA4 were highly expressed in the high-risk group, whereas SIGLEC15 was lowly expressed. The distribution of somatic mutations between the high and low risk groups were also analyzed. The top ten mutated genes in two risk groups were TP53, TTN, KMT2D, MUC16, ARID1A, KDM6A, PIK3CA, SYNE1, KMT2C, and RYR2 (**Supplementary Figure S5**).

### Analyses of immunotherapy response and chemotherapeutic drugs

**Figure 8:**
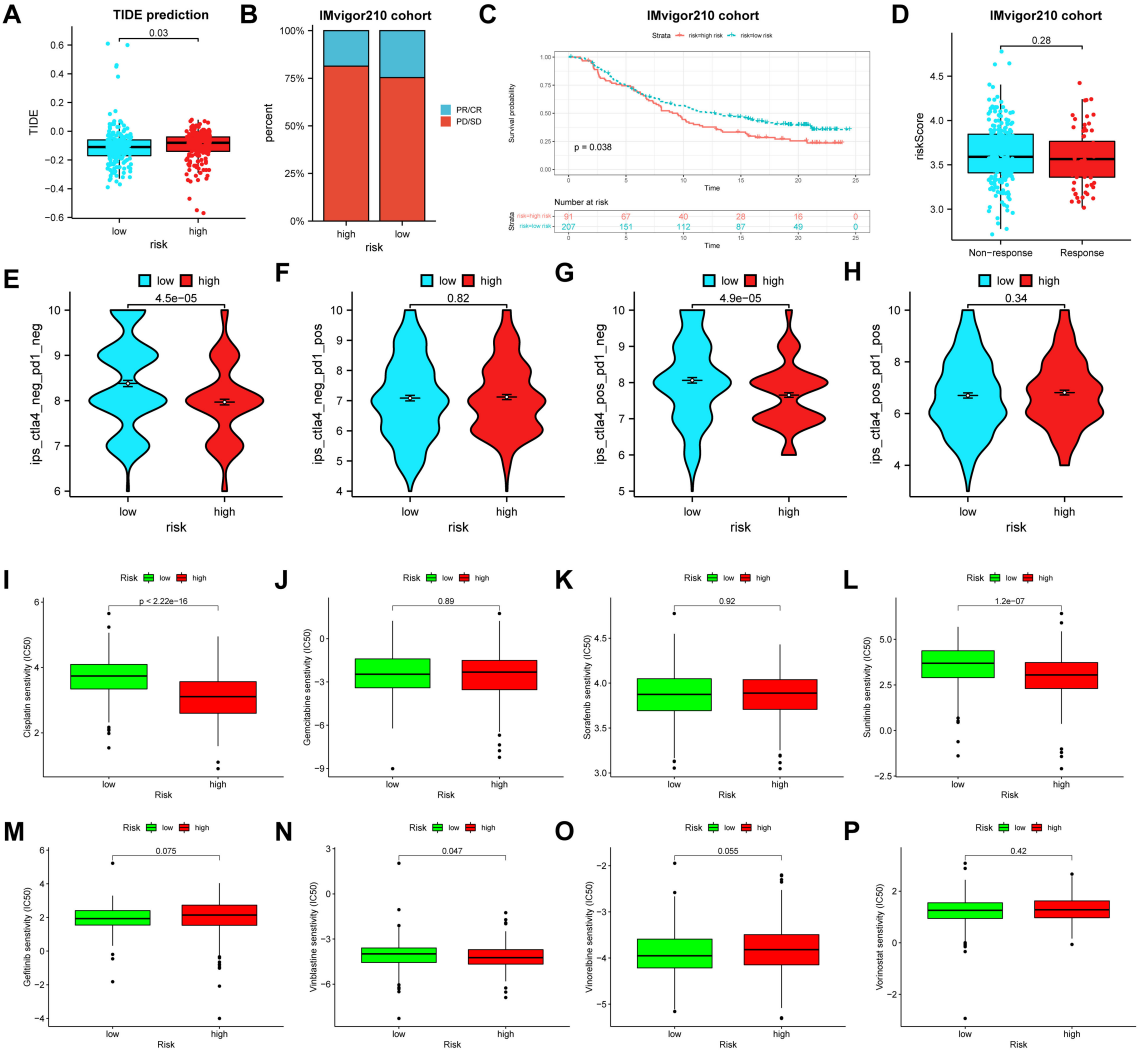
The relationships between the risk score and the tumor immunotherapy and chemotherapeutic sensitivity. **(A)** The TIDE scores between the high- and low-risk groups. **(B)** The proportion of BLCA patients with response to immunotherapy in two risk groups in the IMvigor210 cohort. **(C)** Survival analyses for BLCA patients with high or low risk score in the IMvigor210 cohort. **(D)** Differences in risk scores between the responders and non-responders in the IMvigor210 cohort. **(E-H)** Differences in the IPS between high- and low-risk groups stratified by CTLA4 and PD-1. **(I-P)** Relationships between the risk score and the chemotherapeutic sensitivity. TIDE, tumor immune dysfunction and exclusion; BLCA, bladder cancer. IPS, immunophenoscore.

TIDE is a newly predictor for assessing the response of tumor immunotherapy. In our study, the results indicated that BLCA patients in the high-risk group had higher TIDE scores than those in the low-risk group, suggesting a greater likelihood of tumor immune escape in the high-risk group (*P* = 0.03). To further estimate the prediction of immunotherapy response of our model, the immunotherapy cohort (IMvigor210 cohort) was utilized. As shown in **Figure 8A**, the frequency of partial response (PR)/complete response (CR) was higher in the low-risk group than that in the high-risk group (**Figure 8B**). Moreover, the survival rate demonstrated a significant difference between two risk groups in the IMvigor210 cohort (*P* = 0.038) (**Figure 8C**). However, the risk scores between the immunotherapy-responsive group and the immunotherapy non-responsive group had no statistical difference (*P* = 0.28) (**Figure 8D**). IPS is also a biomarker to assess the immunotherapy response of anti-PD1 and anti-CTLA4 therapies. The results demonstrated that the group of the low-risk group had a significantly higher IPS (ips_ctla4_neg_pd1_neg and ips_ctla4_pos_pd1_neg) compared to the high-risk group, suggesting that BLCA patients with the low-risk had more sensitivity to immunotherapy. However, there was no significant difference between the high-risk and low-risk groups in ips_ctla4_neg_pd1_pos score, and ips_ctla4_pos_pd1_pos score (**Figures 8E-H**).

To evaluate whether the risk score and chemotherapeutic efficacy were associated with bladder cancer (BLCA) treatment, we examined the relationship between the risk score and eight common chemotherapeutic agents, including gefitinib, sunitinib, cisplatin, vinorelbine, vinblastine, gemcitabine, vorinostat, and sorafenib. The results showed that the IC50 values for cisplatin (*P* < 0.001), sunitinib (*P* < 0.001), and vinblastine (*P* = 0.047) were significantly lower in the high-risk group compared to the low-risk group. However, other chemotherapeutic agents had no significant differences (**Figures 8I-P**).

### Verification of the crucial genes in the anoikis-related model

**Figure 9:**
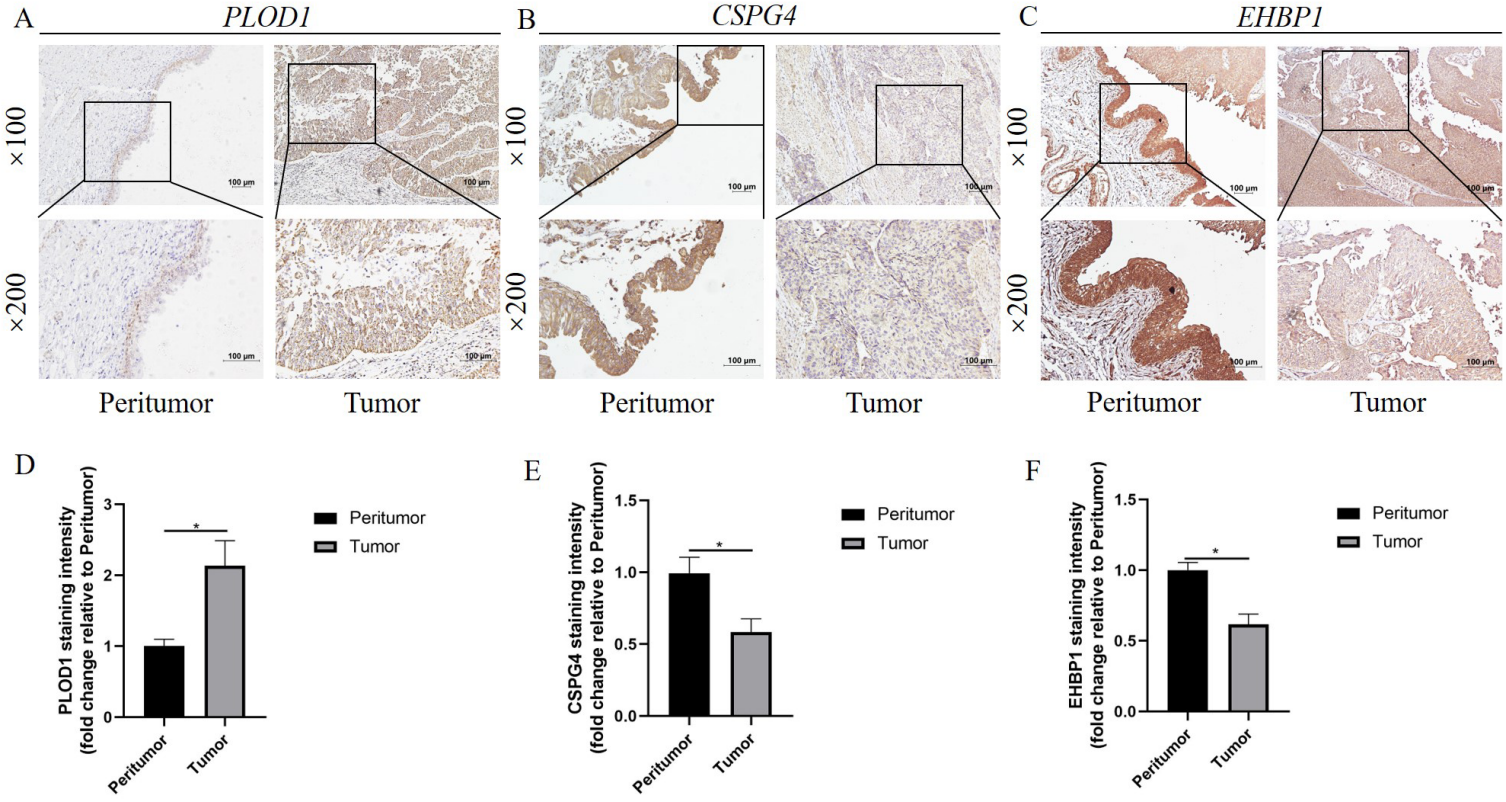
Verification of the three crucial genes in the anoikis-related model. **(A-C)** Representative images of immunohistochemistry staining for **(A)** PLOD1, **(B)** CSPG4, and **(C)** EHBP1 in tumor and peritumor tissue. **(D-F)** The staining intensity of **(D)** PLOD1, **(E)** CSPG4, and **(F)** EHBP1 in tumor and peritumor tissue. *P<0.05.

**Figure 10:**
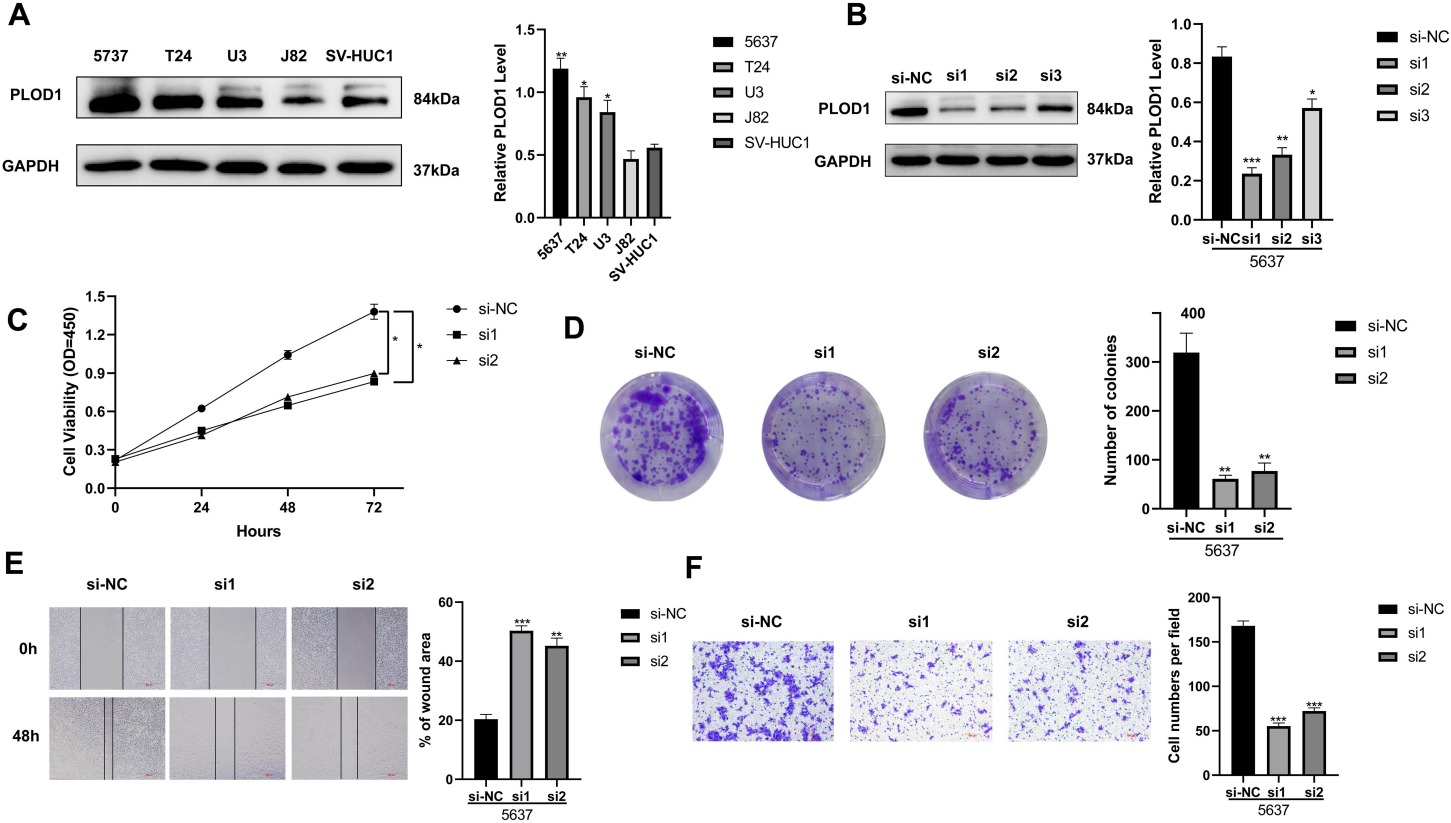
Functional experiments were performed to explore the biological significance of PLOD1. **(A)** The protein levels of PLOD1 were analyzed by the western blot in 5637, T24, U3, J82, and SV-HUC1 cells. **(B)** PLOD1 knockdown efficiency was evaluated by the western blot. **(C, D)** The CCK-8 and colony formation assays demonstrated that down-regulation of PLOD1 inhibited cell proliferation. **(E)** Wound-healing assays detected the effects of knockdown of PLOD1 on cell migration. **(F)** The effect of knockdown of PLOD1 on BLCA cell migration based on transwell assays. *P < 0.05, **P < 0.01, ***P < 0.001.

In our study, three crucial genes (PLOD1, EHBP, and CSPG4) were measured by immunohistochemistry in BLCA and adjacent tissues. The protein level of PLOD1 was higher in BLCA than in the paracancerous tissue, whereas the protein levels of EHBP and CSPG4 were significantly decreased in BLCA (**Figures 9A-F**). Next, PLOD1 was chosen for further functional validation. Western blotting indicated that protein expressions of PLOD1 were upregulated in 5637 (*P* < 0.01), T24 (*P* < 0.05), and U3 (*P* < 0.05) cells than in the SV-HUC1 cells (**Figure 10A**). Then, we knocked down PLOD1 in 5637 cells by siRNA, and western blotting confirmed the knockdown efficiency of PLOD1 (**Figure 10B**). Besides, siRNA1 and siRNA2 were chosen for further functional experiments. CCK-8 and colony formation assays demonstrated that downregulation of PLOD1 suppressed the proliferative ability of the 5637 cells (**Figures 10C, D**). Moreover, wound healing and transwell assays indicated that knockdown of PLOD1 inhibited the migration of 5637 cells (**Figures 10E, F**). In all, downregulation of PLOD1 in 5637 cells could attenuate cell proliferation and migration.

## Discussion

As a commonly occurring urinary tumor, BLCA originates primarily from the urothelium (39). Treatment for bladder cancer has, however, made limited progress. It is common to remove the tumor through the transurethral resection of bladder tumor (TURBT) in NMIBC, but there is a high chance of recurrence, with a 31-78% recurrence risk after 5 years (40). The five-year survival rate for radical cystectomy in MIBC is only 50% (41). Therefore, identifying biomarkers for BLCA is crucial for developing new treatment strategies, many studies have confirmed this. For example, basic helix-loop-helix ARNT like 2 (ARNTL2) facilitates the progression of BLCA via activating ENO1-mediated glycolysis in a SLC31A1-independent and -dependent manner (42). Moreover, studies have clarified that the relationship between the tumor microenvironment (TME) and cellular diversity in bladder cancer (BLCA) progression. Potential biomarkers were predicted by RNA sequencing, and prognosis models of BLCA were constructed to improve prognosis accuracy (43). In this context, in-depth analysis of public data has become a valuable resource for guiding research (44–46).

When ECM attachment is lacking or when cells do not adhere to appropriate locations, anoikis occurs (47). The study has reported that anoikis evasion facilitates metastasis and invasion of tumors (48). Evidence has shown that anoikis plays a vital role in mechanisms of progression of tumors, such as head and neck squamous cell carcinoma, breast cancer, gliomas and hepatocellular carcinoma (49–52). However, a direct link between anoikis and BLCA has not yet been established. Therefore, we have made a number of efforts to investigate the relationship between anoikis and prognosis and treatments in BLCA.

In our research, we presented a comprehensive view of the differential expression of ARGs between tumors and normal tissues in BLCA, as well as the implications for altered immune function. Subsequently, 17 candidate apoptotic-related genes (ARGs) were identified, and potential anoikis subgroups were distinguished based on the expression of these candidate ARGs. Our analysis demonstrated that ARGcluster A had better survival, lower levels of immune infiltration, lower expression levels of HLA genes, lower expression levels of TIGIT, PDCD1LG2, PDCD1, LAG3, HAVCR2, CTLA4, and CD274, and lower ESTIMATE scores, immune scores, and stromal scores than ARGcluster B. The ARGcluster B had lower tumor purity than ARGcluster A. The association between low tumor purity and poor prognosis has been well-documented in previous studies (53, 54). Taken together, ARGcluster B exhibited a stronger immunosuppressive TME. The tumor cells within the immunosuppressive TME were capable of evading the immune cells and were highly malignant, which led to a shorter OS (55). In our study, BLCA patients in the ARGcluster B had a poorer OS, which was consistent with this observation. The complex molecular events through which anoikis promotes metastasis involve a combination of pro-metastatic properties acquired by cancer cells and a tumor microenvironment that facilitates or supports metastasis (56).

Moreover, 446 anoikis phenotype-associated DEGs were obtained, and biological pathways, such as ECM-receptor interaction, cytokine-cytokine receptor interaction, and protein digestion and absorption, were explored based on these DEGs. We also distinguished the gene subtypes via the consensus clustering analysis. The geneCluster C group had the worst OS among three clusters, while the geneCluster A group had the best. Besides, most of the expression of candidate ARGs significantly differed among three clusters, indicating that our gene subtypes were closely related to the anoikis phenotypes.

Then, 404 BLCA patients were randomly divided into the training set (n = 202) and the testing set (n = 202). Next, the effective and robust anoikis-related model in BLCA was built via the univariate Cox, LASSO and multivariate Cox analyses, and its predictive power was revealed. In our study, a robust anoikis-related model was developed based on the expression levels of three genes: EHBP1, CSPG4, and PLOD1.The presence of these three genes has been linked to a variety of malignant tumors. For example, a genome-wide association study has linked EHBP1 to aggressive prostate cancer (57). Ghalali et al. observed that statin-induced P2X7 signaling is linked to aggressive prostate cancer via EHBP1. When P2X7 signaling was activated, EHBP1 translocation was rapid, and EHBP1 knockdown prevented both atorvastatin-induced inhibitions of invasion and nuclear depletion of pAkt (58). The CSPG4 gene had been described as a potential target for cancer immunotherapy, and it was known to influence various immune cell subsets, indicating a potential role in immunotherapy efficacy (59). There is a strong correlation between CSPG4 expression and poor prognosis in aggressive thyroid cancers. An enormous number of CSPG4 peptides eluted by HLA-DQ were identified in ATC, indicating the potential of CSPG4 as an immunotherapeutic target (60). A high level of PLOD1 expression has been documented in malignant tumors, such as BLCA, gastric cancer, glioblastoma, colorectal cancer, and esophageal squamous cell carcinoma. Evidence suggests that PLOD1 overexpression may contribute to increased invasiveness and the mesenchymal subtype (MES) of glioblastoma, indicating that PLOD1 could serve as a potential treatment target for mesenchymal glioblastoma, and possibly for all types of glioblastoma. Evidence suggests that PLOD1 overexpression may contribute to increased invasiveness and the mesenchymal subtype (MES) of glioblastoma, indicating that PLOD1 could serve as a potential treatment target for mesenchymal glioblastoma, and possibly for all types of glioblastoma (61). Chen et al. found that genes in the PLOD family were involved in immune responses and tumor-infiltrating immune cells in BLCA (62). Based on these results, these three genes might be utilized in the diagnosis and treatment of tumors.

Kaplan-Meier survival analysis showed that BLCA patients in the high-risk group had significantly poorer overall survival (OS) compared to those in the low-risk group. Similar results were also confirmed in the three validation datasets (the testing set, the entire set, and GSE13507). Our risk score also had a significant association with the clinicopathological characteristics (tumor grade and tumor stage). Moreover, the anoikis-related model was proved to be independent predictor in BLCA in accordance with the results of Cox regression analyses. The nomogram is widely utilized as a survival prediction tool in various tumors (63). Therefore, the nomogram was developed by integrating age, stage and the risk score to facilitate the use of anoikis-related models in clinics. The calibration plots indicated that the nomogram model was highly accurate at predicting OS of patients, and DCA illustrated that the nomogram model yielded a better benefit in BLCA. Our new nomogram may help clinician predict survival status of BLCA patients, improve risk stratification, and provide more personalized treatment than previously possible. Thanks again for your sincere help and reminder. In all, our anoikis-related model provided excellent predictions of BLCA patient prognoses.

The relationships between the anoikis-related model and immune status and pathways were also explored. A variety of algorithms were utilized to investigate associations with the immune cell infiltration and risk score. We found that some immune cells, such as B cell memory, myeloid dendritic cell activated, T cell follicular helper, monocyte, and myeloid dendritic cell activated were negatively correlated with our riskScore. However, other immune cells, such as T cell CD4+ memory resting, T cell CD4+ memory activated, macrophage M0, mast cell resting, and so on were positively correlated with our riskScore. The results of ssGSEA demonstrated that our risk score also had significant association with 13 immune-related pathways. A common form of intravesical therapy is BCG, and several mechanisms are thought to trigger local immune response following intravesical BCG administration, such as increased levels of urinary cytokine, elevated expression of interferon gamma, and inhibition of tumor growth (64, 65). Recently, the effects of immunotherapy in BLCA have been expanding. The use of ICIs has grown rapidly due to the approval of second-line therapy for BLCA patients have failed platinum-based chemotherapy before (66). Immunotherapy is increasingly being used to treat advanced bladder cancer (BLCA), particularly through the use of PD-L1 inhibitors, which suppress immune evasion by blocking the interaction between PD-1 and PD-L1 (67). It is widely used to predict disease outcomes in patients treated with ICIs based on TIDE score, which measures tumor immune escape at different levels of cytotoxic T lymphocytes (68, 69). Our study demonstrated that the TIDE scores of BLCA patients in the high-risk group were higher than those in the low-risk group. IPS is another biomarker to assess the immunotherapy response. According to the results, in comparison with the high-risk group, the low-risk group had a significantly higher IPS. IPS scores that are high are associated with greater immunogenicity, whereas TIDE scores that are high are associated with more likely tumor immune escape (69, 70). As a result, patients with high IPS scores and low TIDE scores have a better response to ICIs. In our study, low-risk BLCA patients had lower TIDE scores and higher IPS scores, indicating that low-risk patients were highly immunogenic, and were more sensitive to ICIs treatment. Moreover, we examined the difference in sensitivity between high-risk and low-risk groups for conventional chemotherapy drugs. Patients with higher risk scores reacted more strongly to gemcitabine, vincristine, and sorafenib.

Our study also has some shortcomings. First, in light of the preliminary validation of the bioinformatics analysis in BLCA, additional validation is required in a large cohort. Next, we investigated the biological functions of PLOD1 in BLCA, and further cell and animal experiments are needed to elucidate the underlying mechanisms of PLOD1’s role. Nevertheless, A comprehensive study was conducted on the prognostic and immunological significance of anoikis in BLCA.

## Conclusions

In summary, our study illustrated the landscape of candidate ARGs in BLCA. Two definite anoikis subgroups were identified, and ARG cluster B was characterized by an immunosuppressive microenvironment, and worse OS. Moreover, the anoikis-related model was constructed to predict prognosis, and the relationships between the risk score and clinical characteristics, immune cell infiltration, immunotherapy response, and antitumor drug sensitivity were investigated, which might help to understand the tumor features and guide individual immunotherapy strategies. Last but not least, the knockdown of PLOD1 could suppress proliferation and invasion abilities in BLCA cell lines.

## Data Availability

The original contributions presented in the study are included in the article/**Supplementary Materials**. Further inquiries can be directed to the corresponding authors.

## Supplementary material

Supplementary Figure 1 Consensus clustering analysis performed to classify patients into different gene subtypes. **(A)** Three subgroups (k = 3) and their correlation area defined by consensus matrix heatmap. **(B)** The consensus clustering CDF. **(C)** The analysis of the variation in area under the CDF region. CDF, cumulative distribution function.

Supplementary Figure 2
**(A)** The forest plot of the univariate Cox in the training set. **(B)** The cross-validation fit plot of LASSO Cox analysis in the training set. **(C)** The coefficients of three genes measured by the multivariate Cox in the training set.

Supplementary Figure 3
**(A)** Sankey plot indicated the subtype distributions in risk groups with different risk scores and survival status. **(B)** Differences in risk scores between anoikis subtypes.

Supplementary Figure 4 ROC curves for predicting the 1-, 3-, and 5-year ROC curves in the **(A)** training, **(B)** testing, **(C)** TCGA, and **(D)** GSE13507 sets. ROC, receiver operating characteristic.

Supplementary Figure 5 The waterfall plot of somatic mutation features established with low- and high-risk groups.

Supplementary Figure 6 Original Western blot images of **Figure 10A**.

Supplementary Figure 7 Original Western blot images of **Figure 10B**.

## References

[1] Kamat AM Hahn NM Efstathiou JA Lerner SP Malmström PU Choi W. Bladder cancer. Lancet. (2016) 388:2796–810. doi: 10.1016/S0140-6736(16)30512-8 27345655

[2] Lenis AT Lec PM Chamie K Mshs MD. Bladder cancer: A review. Jama. (2020) 324:1980–91. doi: 10.1001/jama.2020.17598 33201207

[3] Siegel RL Miller KD Jemal A. Cancer statistics, 2017. CA Cancer J Clin. (2017) 67:7–30. doi: 10.3322/caac.21387 28055103

[4] Sung H Ferlay J Siegel RL Laversanne M Soerjomataram I Jemal A. Global cancer statistics 2020: GLOBOCAN estimates of incidence and mortality worldwide for 36 cancers in 185 countries. CA Cancer J Clin. (2021) 71:209–49. doi: 10.3322/caac.21660 33538338

[5] Slovacek H Zhuo J Taylor JM. Approaches to non-muscle-invasive bladder cancer. Curr Oncol Rep. (2021) 23:105. doi: 10.1007/s11912-021-01091-1 34269918

[6] Wang Y Zhu H Zhang L He J Bo J Wang J. Common immunological and prognostic features of lung and bladder cancer via smoking-related genes: PRR11 gene as potential immunotherapeutic target. J Cell Mol Med. (2024) 28:e18384. doi: 10.1111/jcmm.v28.10 38760964 PMC11101993

[7] Witjes JA Bruins HM Cathomas R Compérat EM Cowan NC Gakis G. European association of urology guidelines on muscle-invasive and metastatic bladder cancer: summary of the 2020 guidelines. Eur Urol. (2021) 79:82–104. doi: 10.1016/j.eururo.2020.03.055 32360052

[8] Lopez-Beltran A Cimadamore A Blanca A Massari F Vau N Scarpelli M. Immune checkpoint inhibitors for the treatment of bladder cancer. Cancers (Basel). (2021) 13:131. doi: 10.3390/cancers13010131 33401585 PMC7795541

[9] Balar AV Galsky MD Rosenberg JE Powles T Petrylak DP Bellmunt J. Atezolizumab as first-line treatment in cisplatin-ineligible patients with locally advanced and metastatic urothelial carcinoma: a single-arm, multicentre, phase 2 trial. Lancet. (2017) 389:67–76. doi: 10.1016/S0140-6736(16)32455-2 27939400 PMC5568632

[10] Wang Y Wang J Zhang L He J Ji B Wang J. Unveiling the role of YARS1 in bladder cancer: A prognostic biomarker and therapeutic target. J Cell Mol Med. (2024) 28:1–20. doi: 10.1111/jcmm.18213 PMC10951887 38506098

[11] Stenehjem DD Tran D Nkrumah MA Gupta S. PD1/PDL1 inhibitors for the treatment of advanced urothelial bladder cancer. Onco Targets Ther. (2018) 11:5973–89. doi: 10.2147/OTT.S135157 PMC6157986 30275703

[12] Wang Y Wang J Liu Y Wang X Ren M. Multidimensional pan-cancer analysis of HSPA5 and its validation in the prognostic value of bladder cancer. Heliyon. (2024) 10:e27184. doi: 10.1016/j.heliyon.2024.e27184 38496902 PMC10944199

[13] Chiarugi P Giannoni E. Anoikis: a necessary death program for anchorage-dependent cells. Biochem Pharmacol. (2008) 76:1352–64. doi: 10.1016/j.bcp.2008.07.023 18708031

[14] Frisch SM Ruoslahti E. Integrins and anoikis. Curr Opin Cell Biol. (1997) 9:701–6. doi: 10.1016/S0955-0674(97)80124-X 9330874

[15] Kim YN Koo KH Sung JY Yun UJ Kim H. Anoikis resistance: an essential prerequisite for tumor metastasis. Int J Cell Biol. (2012) 2012:306879. doi: 10.1155/2012/306879 22505926 PMC3296207

[16] Adeshakin FO Adeshakin AO Afolabi LO Yan D Zhang G Wan X. Mechanisms for modulating anoikis resistance in cancer and the relevance of metabolic reprogramming. Front Oncol. (2021) 11:626577. doi: 10.3389/fonc.2021.626577 33854965 PMC8039382

[17] Kakavandi E Shahbahrami R Goudarzi H Eslami G Faghihloo E. Anoikis resistance and oncoviruses. J Cell Biochem. (2018) 119:2484–91. doi: 10.1002/jcb.v119.3 28836703

[18] Yu Y Song Y Cheng L Chen L Liu B Lu D. CircCEMIP promotes anoikis-resistance by enhancing protective autophagy in prostate cancer cells. J Exp Clin Cancer Res. (2022) 41:188. doi: 10.1186/s13046-022-02381-7 35655258 PMC9161511

[19] Zhou X Li L Guo X Zhang C Du Y Li T. HBXIP induces anoikis resistance by forming a reciprocal feedback loop with Nrf2 to maintain redox homeostasis and stabilize Prdx1 in breast cancer. NPJ Breast Cancer. (2022) 8:7. doi: 10.1038/s41523-021-00374-x 35027562 PMC8758767

[20] Mason JA Davison-Versagli CA Leliaert AK Pape DJ McCallister C Zuo J. Oncogenic Ras differentially regulates metabolism and anoikis in extracellular matrix-detached cells. Cell Death Differ. (2016) 23:1271–82. doi: 10.1038/cdd.2016.15 PMC4947665 26915296

[21] Song J Liu Y Liu F Zhang L Li G Yuan C. The 14-3-3σ protein promotes HCC anoikis resistance by inhibiting EGFR degradation and thereby activating the EGFR-dependent ERK1/2 signaling pathway. Theranostics. (2021) 11:996–1015. doi: 10.7150/thno.51646 33391517 PMC7738881

[22] Sharma R Gogoi G Saikia S Sharma A Kalita DJ Sarma A. BMP4 enhances anoikis resistance and chemoresistance of breast cancer cells through canonical BMP signaling. J Cell Commun Signal. (2022) 16:191–205. doi: 10.1007/s12079-021-00649-9 34608584 PMC8891411

[23] Moro L Arbini AA Yao JL di Sant'Agnese PA Marra E Greco M. Mitochondrial DNA depletion in prostate epithelial cells promotes anoikis resistance and invasion through activation of PI3K/Akt2. Cell Death Differ. (2009) 16:571–83. doi: 10.1038/cdd.2008.178 19079138

[24] Pang ZQ Wang JS Wang JF Wang YX Ji B Xu YD. JAM3: A prognostic biomarker for bladder cancer via epithelial-mesenchymal transition regulation. Biomol BioMed. (2024) 24:897–911. doi: 10.17305/bb.2024.9979 38400838 PMC11293228

[25] Chen S Gu J Zhang Q Hu Y Ge Y. Development of biomarker signatures associated with anoikis to predict prognosis in endometrial carcinoma patients. J Oncol. (2021) 2021:3375297. doi: 10.1155/2021/3375297 34992654 PMC8727165

[26] Zhang YY Li XW Li XD Zhou TT Chen C Liu JW. Comprehensive analysis of anoikis-related long non-coding RNA immune infiltration in patients with bladder cancer and immunotherapy. Front Immunol. (2022) 13:1055304. doi: 10.3389/fimmu.2022.1055304 36505486 PMC9732092

[27] Wilkerson MD Hayes DN. ConsensusClusterPlus: a class discovery tool with confidence assessments and item tracking. Bioinformatics. (2010) 26:1572–3. doi: 10.1093/bioinformatics/btq170 PMC2881355 20427518

[28] Wang Y Zhu H Xu H Qiu Y Zhu Y Wang X. Senescence-related gene c-Myc affects bladder cancer cell senescence by interacting with HSP90B1 to regulate cisplatin sensitivity. Aging (Albany NY). (2023) 15:7408–23. doi: 10.18632/aging.204863 PMC10457043 37433010

[29] Vickers AJ Cronin AM Elkin EB Gonen M. Extensions to decision curve analysis, a novel method for evaluating diagnostic tests, prediction models and molecular markers. BMC Med Inform Decis Mak. (2008) 8:53. doi: 10.1186/1472-6947-8-53 19036144 PMC2611975

[30] Wang Y Zhu H Wang X. Prognosis and immune infiltration analysis of endoplasmic reticulum stress-related genes in bladder urothelial carcinoma. Front Genet. (2022) 13:965100. doi: 10.3389/fgene.2022.965100 36186448 PMC9520708

[31] Wu J Li L Zhang H Zhao Y Zhang H Wu S. A risk model developed based on tumor microenvironment predicts overall survival and associates with tumor immunity of patients with lung adenocarcinoma. Oncogene. (2021) 40:4413–24. doi: 10.1038/s41388-021-01853-y 34108619

[32] Charoentong P Finotello F Angelova M Mayer C Efremova M Rieder D. Pan-cancer immunogenomic analyses reveal genotype-immunophenotype relationships and predictors of response to checkpoint blockade. Cell Rep. (2017) 18:248–62. doi: 10.1016/j.celrep.2016.12.019 28052254

[33] Jiang P Gu S Pan D Fu J Sahu A Hu X. Signatures of T cell dysfunction and exclusion predict cancer immunotherapy response. Nat Med. (2018) 24:1550–8. doi: 10.1038/s41591-018-0136-1 PMC6487502 30127393

[34] Mariathasan S Turley SJ Nickles D Castiglioni A Yuen K Wang Y. TGFβ attenuates tumour response to PD-L1 blockade by contributing to exclusion of T cells. Nature. (2018) 554:544–8. doi: 10.1038/nature25501 PMC6028240 29443960

[35] Wang Y Ji B Zhang L Wang J He J Ding B. Identification of metastasis-related genes for predicting prostate cancer diagnosis, metastasis and immunotherapy drug candidates using machine learning approaches. Biol Direct. (2024) 19:50. doi: 10.1186/s13062-024-00494-x 38918844 PMC11197330

[36] Geeleher P Cox NJ Huang RS. Clinical drug response can be predicted using baseline gene expression levels and in *vitro* drug sensitivity in cell lines. Genome Biol. (2014) 15:R47. doi: 10.1186/gb-2014-15-3-r47 24580837 PMC4054092

[37] Yang W Soares J Greninger P Edelman EJ Lightfoot H Forbes S. Genomics of Drug Sensitivity in Cancer (GDSC): a resource for therapeutic biomarker discovery in cancer cells. Nucleic Acids Res. (2013) 41:D955–61. doi: 10.1093/nar/gks1111 PMC3531057 23180760

[38] Chen Y Zhang Y Tan Y Liu Z. Clinical significance of SPARC in esophageal squamous cell carcinoma. Biochem Biophys Res Commun. (2017) 492:184–91. doi: 10.1016/j.bbrc.2017.08.043 28818666

[39] Jemal A Bray F Center MM Ferlay J Ward E Forman D. Global cancer statistics. CA Cancer J Clin. (2011) 61:69–90. doi: 10.3322/caac.20107 21296855

[40] Teoh JY Kamat AM Black PC Grivas P Shariat SF Babjuk M. Recurrence mechanisms of non-muscle-invasive bladder cancer - a clinical perspective. Nat Rev Urol. (2022) 19:280–94. doi: 10.1038/s41585-022-00578-1 35361927

[41] Vlaming M Kiemeney L van der Heijden AG. Survival after radical cystectomy: Progressive versus *De novo* muscle invasive bladder cancer. Cancer Treat Res Commun. (2020) 25:100264. doi: 10.1016/j.ctarc.2020.100264 33316558

[42] Wang J Ren J Tu X Yuan H Ye Z Wang X. ARNTL2 facilitates bladder cancer progression through potentiating ENO1-mediated glycolysis in a SLC31A1-independent and -dependent manner. Life Sci. (2024) 355:122974. doi: 10.1016/j.lfs.2024.122974 39147318

[43] Safder I Valentine H Uzzo N Sfakianos J Uzzo R Gupta S. Identification and validation of prognostic model for tumor microenvironment-associated genes in bladder cancer based on single-cell RNA sequencing data sets. JCO Precis Oncol. (2024) 8:e2300661. doi: 10.1200/PO.23.00661 39151107 PMC11371085

[44] Luan JC Zeng TY Zhang QJ Xia DR Cong R Yao LY. A novel signature constructed by ferroptosis-associated genes (FAGs) for the prediction of prognosis in bladder urothelial carcinoma (BLCA) and associated with immune infiltration. Cancer Cell Int. (2021) 21:414. doi: 10.1186/s12935-021-02096-3 34362387 PMC8349026

[45] Zhang D Xu X Wei Y Chen X Li G Lu Z. Prognostic role of DNA damage response genes mutations and their association with the sensitivity of olaparib in prostate cancer patients. Cancer Control. (2022) 29:10732748221129451. doi: 10.1177/10732748221129451 36283420 PMC9608002

[46] Liu Y Wang J Li L Qin H Wei Y Zhang X. AC010973.2 promotes cell proliferation and is one of six stemness-related genes that predict overall survival of renal clear cell carcinoma. Sci Rep. (2022) 12:4272. doi: 10.1038/s41598-022-07070-1 35277527 PMC8917182

[47] Bakir B Chiarella AM Pitarresi JR Rustgi AK. EMT, MET, plasticity, and tumor metastasis. Trends Cell Biol. (2020) 30:764–76. doi: 10.1016/j.tcb.2020.07.003 PMC7647095 32800658

[48] Di Micco R Krizhanovsky V Baker D d'Adda di Fagagna F. Cellular senescence in ageing: from mechanisms to therapeutic opportunities. Nat Rev Mol Cell Biol. (2021) 22:75–95. doi: 10.1038/s41580-020-00314-w 33328614 PMC8344376

[49] Surette A Yoo BH Younis T Matheson K Rameh T Snowdon J. Tumor levels of the mediators of ErbB2-driven anoikis resistance correlate with breast cancer relapse in patients receiving trastuzumab-based therapies. Breast Cancer Res Treat. (2021) 187:743–58. doi: 10.1007/s10549-021-06164-0 33728523

[50] Chi H Jiang P Xu K Zhao Y Song B Peng G. A novel anoikis-related gene signature predicts prognosis in patients with head and neck squamous cell carcinoma and reveals immune infiltration. Front Genet. (2022) 13:984273. doi: 10.3389/fgene.2022.984273 36092898 PMC9459093

[51] Wang S Lv Y Zhou Y Ling J Wang H Gu D. Acidic extracellular pH induces autophagy to promote anoikis resistance of hepatocellular carcinoma cells via downregulation of miR-3663-3p. J Cancer. (2021) 12:3418–26. doi: 10.7150/jca.51849 PMC8120191 33995620

[52] Zhao S Chi H Ji W He Q Lai G Peng G. A bioinformatics-based analysis of an anoikis-related gene signature predicts the prognosis of patients with low-grade gliomas. Brain Sci. (2022) 12:1349. doi: 10.3390/brainsci12101349 36291283 PMC9599312

[53] Mao Y Feng Q Zheng P Yang L Liu T Xu Y. Low tumor purity is associated with poor prognosis, heavy mutation burden, and intense immune phenotype in colon cancer. Cancer Manag Res. (2018) 10:3569–77. doi: 10.2147/CMAR.S171855 PMC6149864 30271205

[54] Zhang C Cheng W Ren X Wang Z Liu X Li G. Tumor purity as an underlying key factor in glioma. Clin Cancer Res. (2017) 23:6279–91. doi: 10.1158/1078-0432.CCR-16-2598 28754819

[55] Lei X Lei Y Li JK Du WX Li RG Yang J. Immune cells within the tumor microenvironment: Biological functions and roles in cancer immunotherapy. Cancer Lett. (2020) 470:126–33. doi: 10.1016/j.canlet.2019.11.009 31730903

[56] Zhou S Xu H Duan Y Tang Q Huang H Bi F. Survival mechanisms of circulating tumor cells and their implications for cancer treatment. Cancer Metastasis Rev. (2024) 43:941–57. doi: 10.1007/s10555-024-10178-7 38436892

[57] Gudmundsson J Sulem P Rafnar T Bergthorsson JT Manolescu A Gudbjartsson D. Common sequence variants on 2p15 and Xp11.22 confer susceptibility to prostate cancer. Nat Genet. (2008) 40:281–3. doi: 10.1038/ng.89 PMC3598012 18264098

[58] Ghalali A Wiklund F Zheng H Stenius U Högberg J. Atorvastatin prevents ATP-driven invasiveness via P2X7 and EHBP1 signaling in PTEN-expressing prostate cancer cells. Carcinogenesis. (2014) 35:1547–55. doi: 10.1093/carcin/bgu019 24451147

[59] Ilieva KM Cheung A Mele S Chiaruttini G Crescioli S Griffin M. Chondroitin sulfate proteoglycan 4 and its potential as an antibody immunotherapy target across different tumor types. Front Immunol. (2017) 8:1911. doi: 10.3389/fimmu.2017.01911 29375561 PMC5767725

[60] Egan CE Stefanova D Ahmed A Raja VJ Thiesmeyer JW Chen KJ. CSPG4 is a potential therapeutic target in anaplastic thyroid. Cancer Thyroid. (2021) 31:1481–93. doi: 10.1089/thy.2021.0067 PMC8917884 34078123

[61] Wang Z Shi Y Ying C Jiang Y Hu J. Hypoxia-induced PLOD1 overexpression contributes to the Malignant phenotype of glioblastoma via NF-κB signaling. Oncogene. (2021) 40:1458–75. doi: 10.1038/s41388-020-01635-y PMC7906902 33420370

[62] Chen R Jiang M Hu B Fu B Sun T. Comprehensive analysis of the expression, prognosis, and biological significance of PLOD family in bladder cancer. Int J Gen Med. (2023) 16:707–22. doi: 10.2147/IJGM.S399875 PMC9975538 36872941

[63] Balachandran VP Gonen M Smith JJ DeMatteo RP. Nomograms in oncology: more than meets the eye. Lancet Oncol. (2015) 16:e173–80. doi: 10.1016/S1470-2045(14)71116-7 PMC4465353 25846097

[64] Böhle A Brandau S. Immune mechanisms in bacillus Calmette-Guerin immunotherapy for superficial bladder cancer. J Urol. (2003) 170:964–9. doi: 10.1097/01.ju.0000073852.24341.4a 12913751

[65] Redelman-Sidi G Glickman MS Bochner BH. The mechanism of action of BCG therapy for bladder cancer–a current perspective. Nat Rev Urol. (2014) 11:153–62. doi: 10.1038/nrurol.2014.15 24492433

[66] Rhea LP Mendez-Marti S Kim D Aragon-Ching JB. Role of immunotherapy in bladder cancer Cancer. Treat Res Commun. (2021) 26:100296. doi: 10.1016/j.ctarc.2020.100296 33421822

[67] Ou SL Luo J Wei H Qin XL Du SY Wang S. Safety and efficacy of programmed cell death 1 and programmed death ligand-1 inhibitors in the treatment of cancer: an overview of systematic reviews. Front Immunol. (2022) 13:953761. doi: 10.3389/fimmu.2022.953761 35911744 PMC9326177

[68] Sato J Kitano S Motoi N Ino Y Yamamoto N Watanabe S. CD20(+) tumor-infiltrating immune cells and CD204(+) M2 macrophages are associated with prognosis in thymic carcinoma. Cancer Sci. (2020) 111:1921–32. doi: 10.1111/cas.v111.6 PMC7293080 32277550

[69] Thorsson V Gibbs DL Brown SD Wolf D Bortone DS Ou Yang TH. The immune landscape of cancer. Immunity. (2019) 51:411–2. doi: 10.1016/j.immuni.2019.08.004 31433971

[70] Zeng C Liu Y He R Lu X Dai Y Qi G. Identification and validation of a novel cellular senescence-related lncRNA prognostic signature for predicting immunotherapy response in stomach adenocarcinoma. Front Genet. (2022) 13:935056. doi: 10.3389/fgene.2022.935056 36092903 PMC9453157

