# Supplementary figures and images for: Identification of anoikis-related molecular patterns and the novel risk model to predict prognosis, tumor microenvironment infiltration and immunotherapy response in bladder cancer
Source: Front Immunol. 2024 Nov 27;15:1491808. doi: 10.3389/fimmu.2024.1491808 (PMC11631915; doi:10.3389/fimmu.2024.1491808)

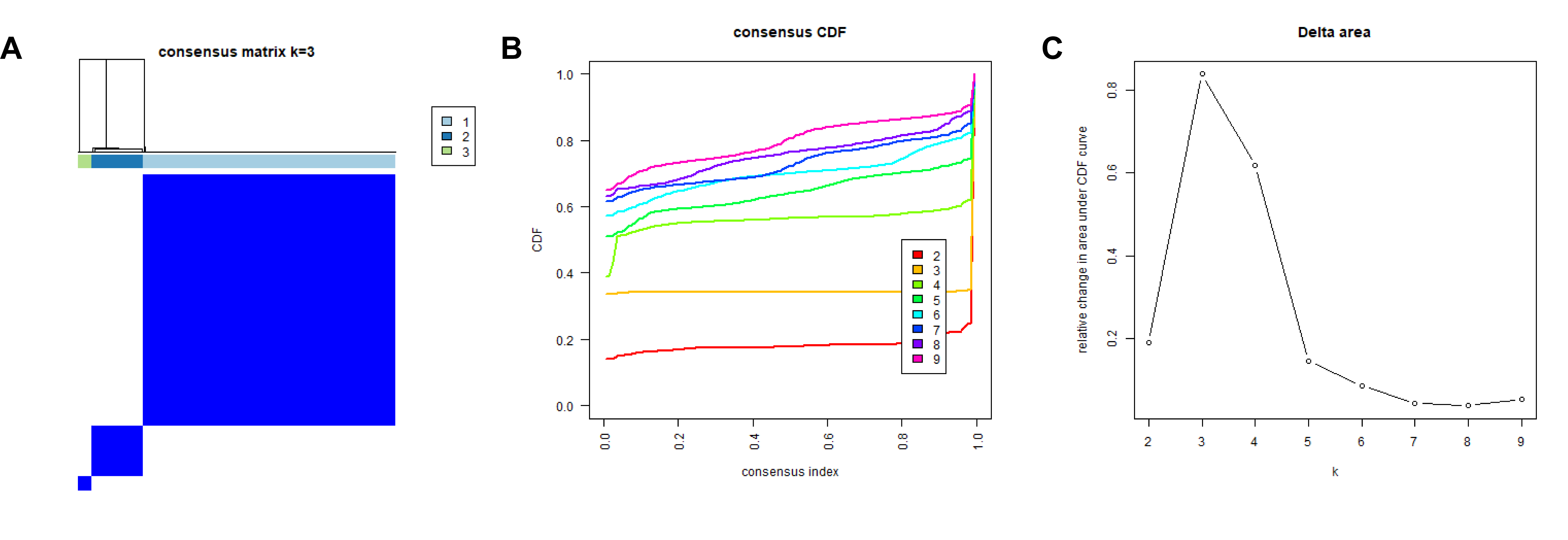

Supplement: Supplementary Figure 1 — Consensus clustering analysis performed to classify patients into different gene subtypes. (A) Three subgroups (k = 3) and their correlation area defined by consensus matrix heatmap. (B) The consensus clustering CDF. (C) The analysis of the variation in area under the CDF region. CDF, cumulative distribution function. [file Image1.jpeg]

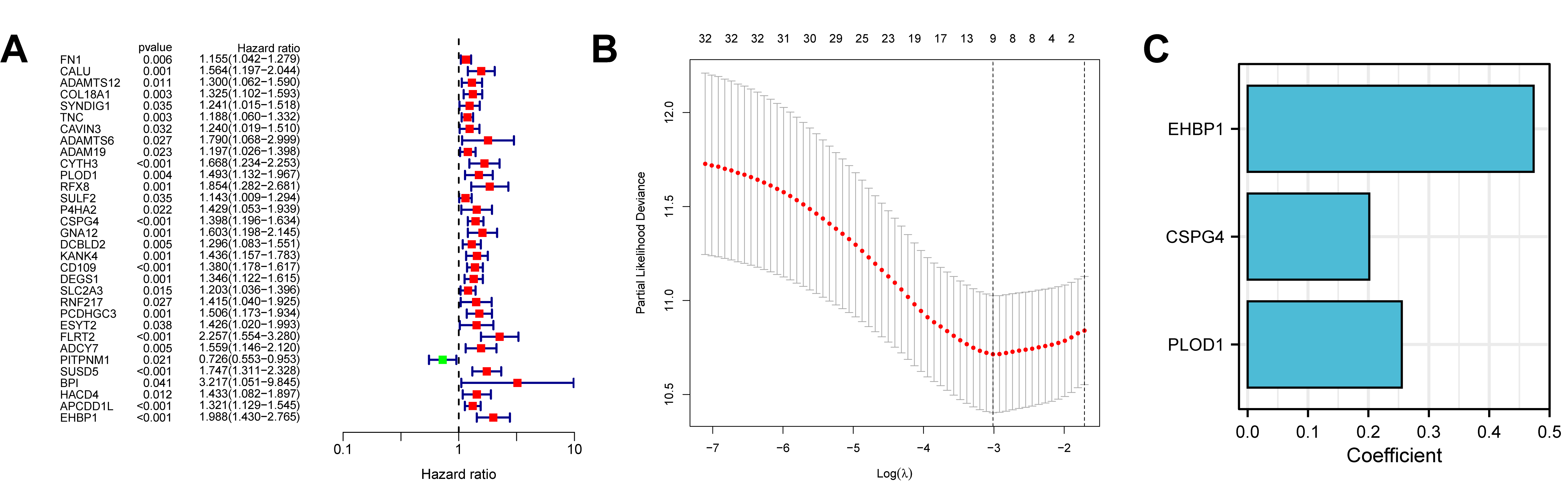

Supplement: Supplementary Figure 2 — (A) The forest plot of the univariate Cox in the training set. (B) The cross-validation fit plot of LASSO Cox analysis in the training set. (C) The coefficients of three genes measured by the multivariate Cox in the training set. [file Image2.jpeg]

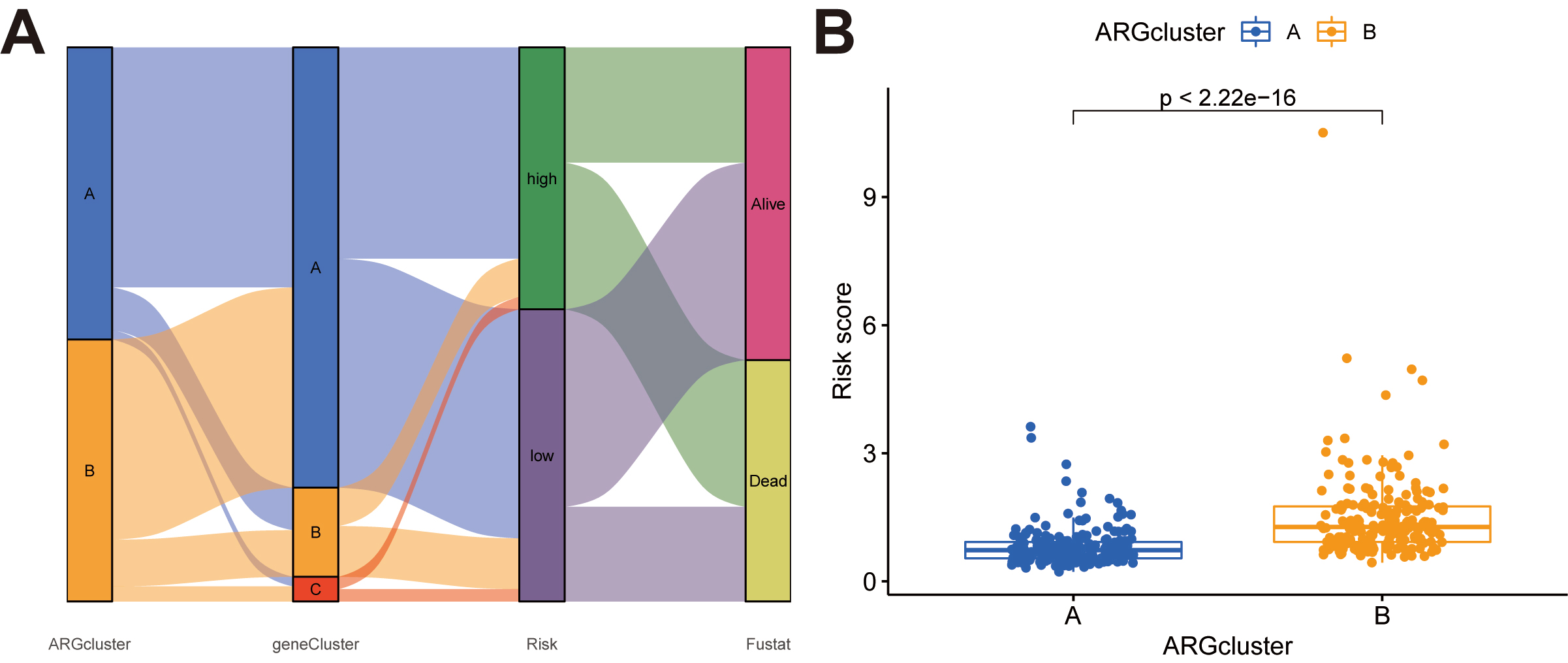

Supplement: Supplementary Figure 3 — (A) Sankey plot indicated the subtype distributions in risk groups with different risk scores and survival status. (B) Differences in risk scores between anoikis subtypes. [file Image3.jpeg]

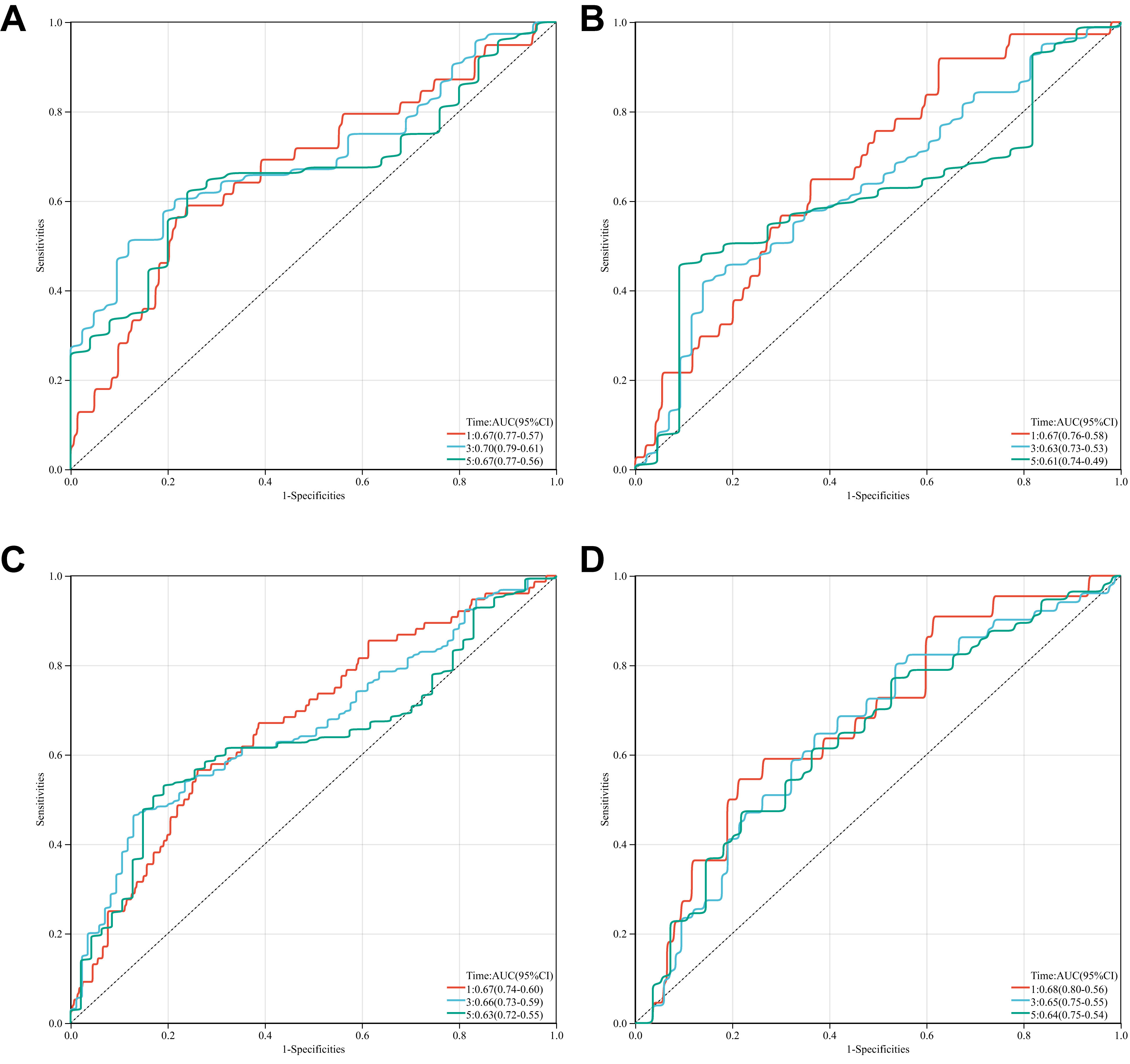

Supplement: Supplementary Figure 4 — ROC curves for predicting the 1-, 3-, and 5-year ROC curves in the (A) training, (B) testing, (C) TCGA, and (D) GSE13507 sets. ROC, receiver operating characteristic. [file Image4.jpeg]

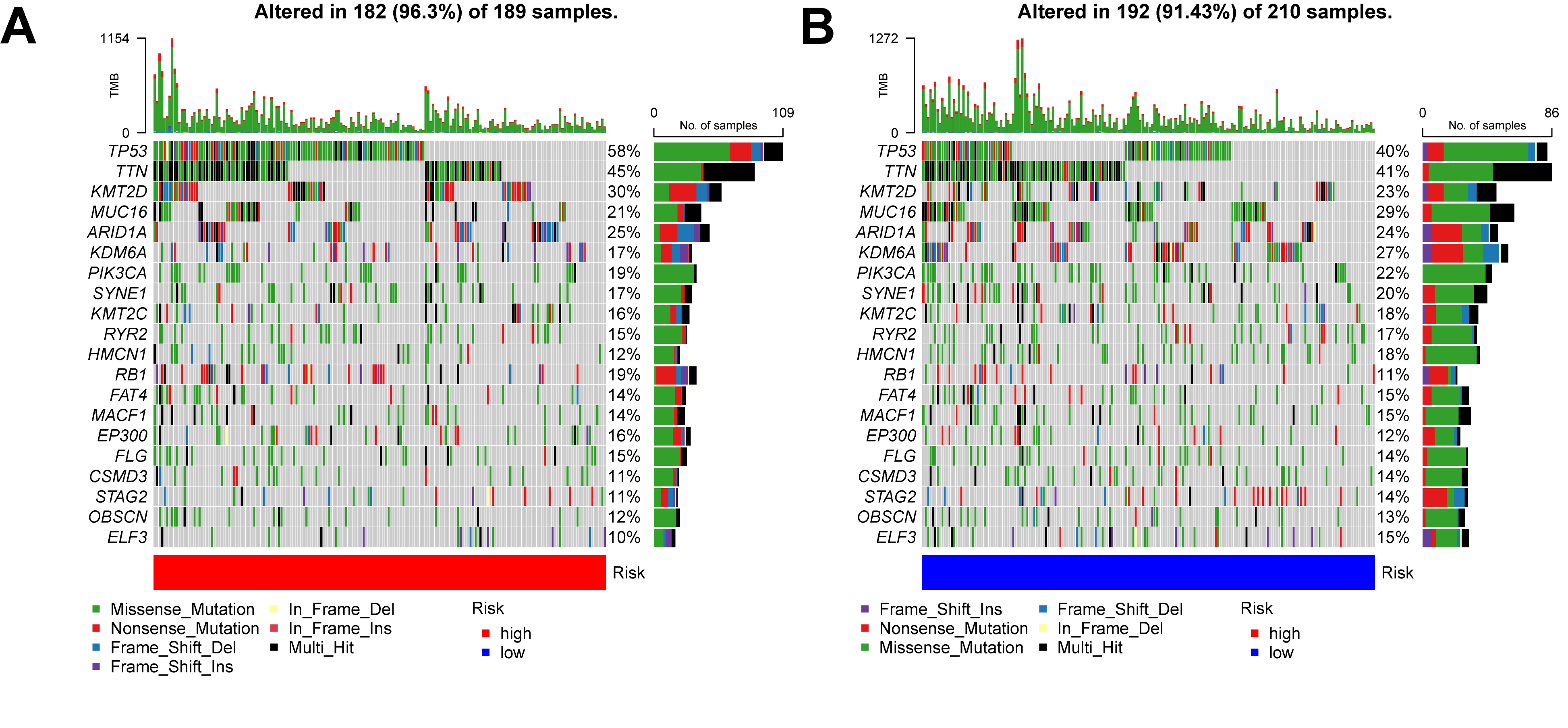

Supplement: Supplementary Figure 5 — The waterfall plot of somatic mutation features established with low- and high-risk groups. [file Image5.jpeg]

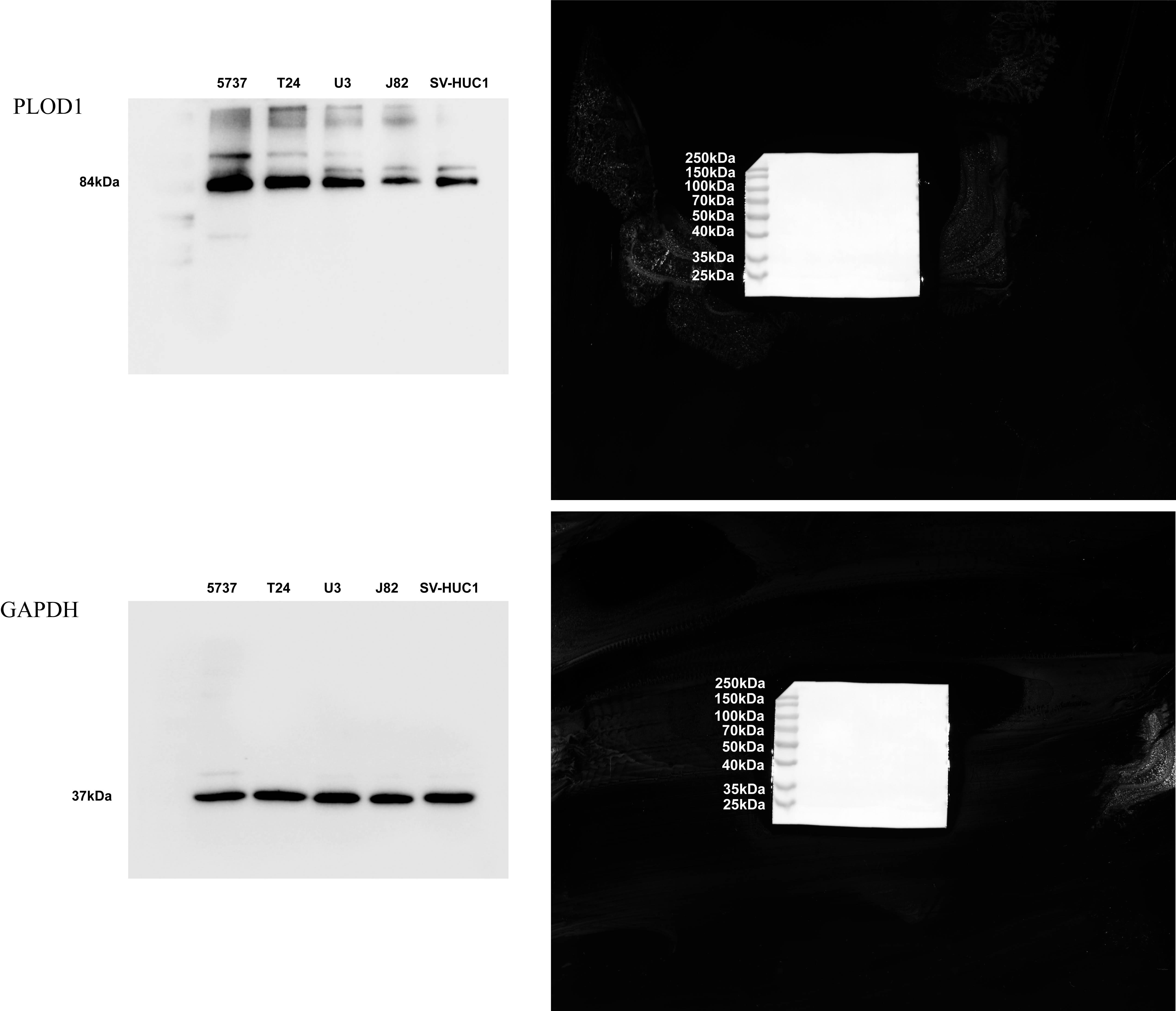

Supplement: Supplementary Figure 6 — Original Western blot images of Figure 10A . [file Image6.png]

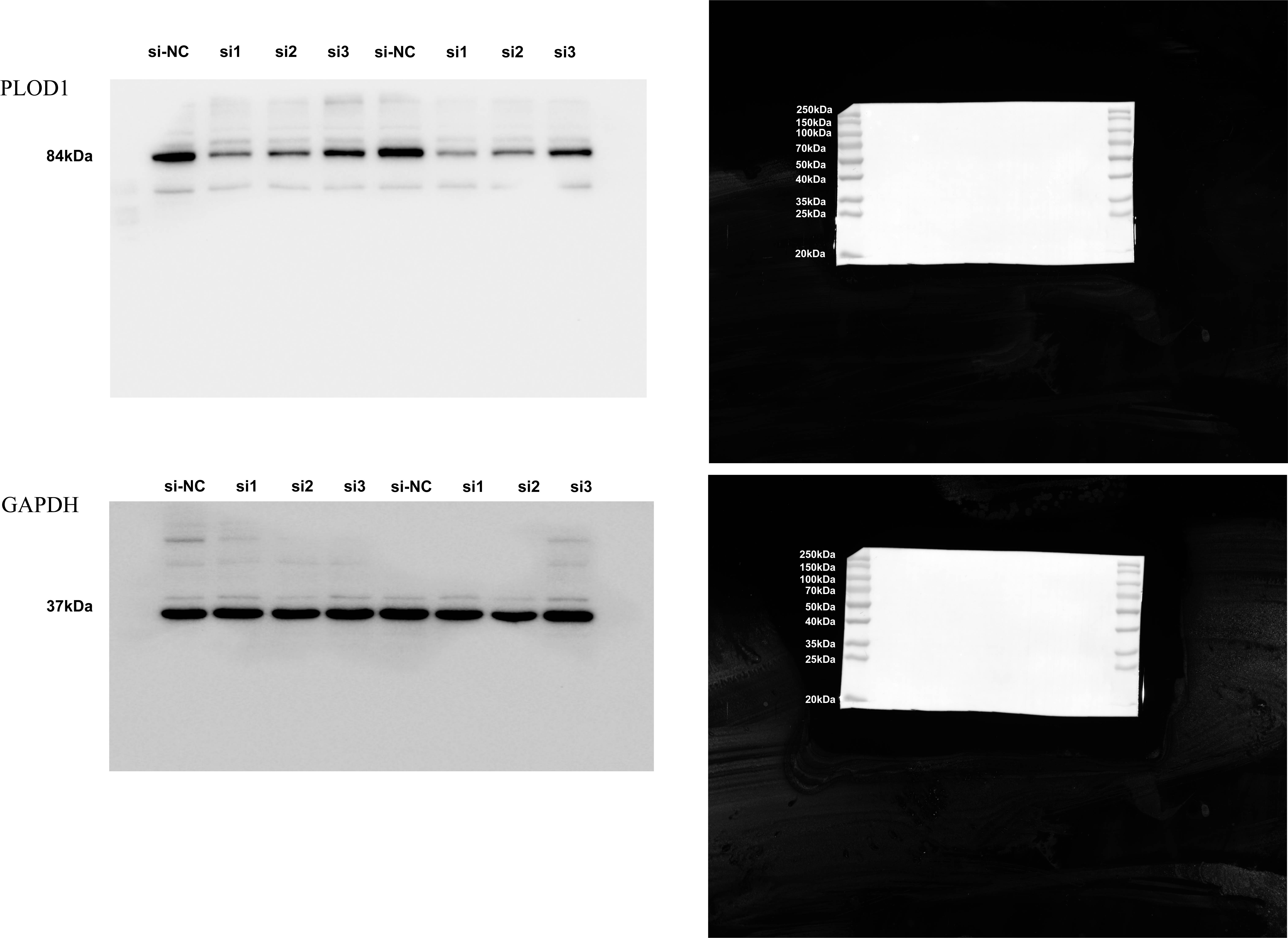

Supplement: Supplementary Figure 7 — Original Western blot images of Figure 10B . [file Image7.png]
