# Supplementary material for: Identification of anoikis-related molecular patterns and the novel risk model to predict prognosis, tumor microenvironment infiltration and immunotherapy response in bladder cancer
Source: Front Immunol. 2024 Nov 27;15:1491808. doi: 10.3389/fimmu.2024.1491808 (PMC11631915; doi:10.3389/fimmu.2024.1491808)
Supplement: Supplementary file 8 [file Table1.docx]

**Table S1: A total of 434 anoikis-related genes (ARGs).**

ABHD2

ABHD4

ABL1

ACP1

ACTG1

ADAMTSL1

ADCY10

AFAP1L1

AFP

AKT1

AKT2

AKT3

ANGPTL2

ANGPTL4

ANKRD13C

ANXA2

ANXA5

APOBEC3G

AR

ARHGDIA

ARHGDIB

ARHGEF7

ATF2

ATF4

BAD

BAG1

BAG4

BAK1

BAX

BCAR1

BCL2

BCL2L1

BCL2L11

BCL2L15

BCL2L2

BDNF

BID

BIN1

BIRC3

BIRC5

BMF

BMP6

BNIP3

BNIP3L

BRAF

BRCA2

BRMS1

BSG

BST2

BUB1

BUB3

CALR

CASP10

CASP2

CASP3

CASP6

CASP8

CASP9

CAV1

CBL

CCAR2

CCDC178

CCDC80

CCN1

CCN2

CCN6

CCND1

CCR7

CD151

CD24

CD36

CD44

CD63

CDC25C

CDC42

CDCP1

CDH1

CDH2

CDH3

CDK1

CDK11A

CDK11B

CDKN1A

CDKN1B

CDKN2A

CDKN3

CEACAM1

CEACAM3

CEACAM4

CEACAM5

CEACAM6

CEACAM8

CEBPB

CEMIP

CFLAR

CHEK2

CLDN1

CLU

COL13A1

COL4A2

CPEB2

CPT1A

CRABP2

CRYAB

CRYBA1

CSK

CSNK2A1

CSPG4

CTBP1

CTNNA1

CTNNB1

CTNND1

CTTN

CXCL12

CXCL8

CXCR4

CYCS

DAP3

DAPK1

DAPK2

DLG1

DOCK1

DYNLL2

E2F1

EDA2R

EDAR

EDIL3

EEF1A1

EEF2K

EGF

EGFR

EHMT2

EIF2AK3

ELANE

ELK1

ENDOG

EPHA2

EPHB6

ERBB2

ETV4

EZH2

EZR

F10

F3

FADD

FAS

FASLG

FASN

FBLIM1

FBXW7-AS1

FER

FGF2

FN1

FOXA1

FOXC2

FOXO3

GDF2

GKN1

GLI2

GLO1

GLUD1

GNE

GRHL2

GSK3B

HAVCR2

HGF

HIF1A

HK2

HMCN1

HMGA1

HMOX1

HOTAIR

HRAS

HSP90B1

HSPB1

HTRA1

ID2

IFI27

IGF1

IGF1R

IKBKG

IL6

ILK

INHBB

IQGAP1

IRF6

ITGA2

ITGA3

ITGA4

ITGA5

ITGA6

ITGA8

ITGAV

ITGB1

ITGB3

ITGB4

ITGB5

ITPRIP

JUP

KDM3A

KDR

KL

KLF12

KRAS

KRT14

LAMA3

LAMB3

LAMC2

LATS1

LDHA

LGALS1

LGALS3

LMO3

LPAR1

LRP1

LTB4R2

LTF

MAD2L1

MALAT1

MAOA

MAP2K1

MAP2K2

MAP3K1

MAP3K7

MAPK1

MAPK11

MAPK3

MAPK8

MAVS

MCL1

MDM2

MET

MGAT5

MIR107

MIR10A

MIR124-1

MIR141

MIR145

MIR181A1

MIR1827

MIR200A

MIR200B

MIR200C

MIR204

MIR21

MIR26A1

MIR30B

MIR30C1

MIR363

MIR525

MIR630

MMP11

MMP13

MMP2

MMP9

MNX1

MSLN

MTA1

MTDH

MTOR

MUC1

MYBBP1A

MYC

MYH9

MYO5A

NDRG1

NFE2L2

NGF

NKX2-1

NOTCH1

NOTCH3

NOX4

NQO1

NRAS

NTF3

NTRK1

NTRK2

NTRK3

OCLN

OGT

OLFM3

ONECUT1

PAK1

PAK2

PAK3

PAK4

PARP1

PBK

PCNA

PDCD4

PDCD6IP

PDGFRB

PDK4

PDPK1

PHLDA2

PIK3C2B

PIK3CA

PIK3CB

PIK3CG

PIK3R1

PIK3R2

PIK3R3

PIN1

PIP5K1C

PITPNC1

PLAT

PLAU

PLAUR

PLG

PLK1

PPARG

PPP1R13B

PPP2CA

PPP2R1A

PPP2R2D

PPP2R5A

PRDX4

PRKACA

PRKCA

PRKCI

PRKCQ

PRKD1

PRPF4B

PTEN

PTGS2

PTHLH

PTK2

PTK2B

PTK6

PTPN1

PTPN11

PTRH2

PXN

PYCARD

QSOX1

RAC1

RAC3

RACK1

RAD9A

RAF1

RANBP9

RB1

RBFOX2

RBL2

RELA

RHOA

RHOB

RHOC

RHOG

RHOQ

RIPK1

ROCK1

RPS6KA3

RPS6KB1

S100A4

S100A7

SATB1

SCRIB

SDCBP

SERPINA1

SERPINB1

SERPINE1

SESN1

SESN2

SESN3

SETD2

SFN

SFRP1

SH3GLB1

SHC1

SIK1

SIK2

SIRPA

SIRT1

SIRT3

SIRT6

SKP2

SLC2A1

SLC39A6

SLCO1B3

SMAD4

SMAD7

SMARCE1

SNAI2

SOD2

SP1

SPHK1

SPIB

SPINK1

SPP1

SRC

SRSF3

STAT3

STK11

STK38

TAGLN

TDGF1

TFDP1

TGFB1

THBS1

TIAM1

TIMP1

TLE1

TLE5

TLN1

TLR3

TNC

TNFRSF10B

TNFRSF12A

TNFRSF1A

TNFSF10

TP53

TP63

TP73

TPM1

TRAF2

TRIM31

TSC2

TSG101

TUBB3

TWIST1

UBE2C

VEGFA

VPS37A

VTN

XAF1

XIAP

YAP1

YWHAZ

ZBTB7A

ZEB1

ZEB2

ZNF304

ZNF32
