# Supplementary material for: Identification of anoikis-related molecular patterns and the novel risk model to predict prognosis, tumor microenvironment infiltration and immunotherapy response in bladder cancer
Source: Front Immunol. 2024 Nov 27;15:1491808. doi: 10.3389/fimmu.2024.1491808 (PMC11631915; doi:10.3389/fimmu.2024.1491808)
Supplement: Supplementary file 9 [file Table2.docx]

**Table S2: The relevant sequences of siRNA PLOD1.**

| Name | Sequence(5’-3’) |
| --- | --- |
| PLOD1-142(F) | GCUCAGUUCUUCAACUACAAGTT |
| PLOD1-142(R) | CUUGUAGUUGAAGAACUGAGCTT |
| PLOD1-867(F) | GGUCGGCGUGUUCAUCGAACATT |
| PLOD1-867(R) | UGUUCGAUGAACACGCCGACCTT |
| PLOD1-1023(F) | GCGAGUACCAGUCUGUGAAGCTT |
| PLOD1-1023(R) | GCGAGUACCAGUCUGUGAAGCTT |
