# Supplementary material for: Identification of anoikis-related molecular patterns and the novel risk model to predict prognosis, tumor microenvironment infiltration and immunotherapy response in bladder cancer
Source: Front Immunol. 2024 Nov 27;15:1491808. doi: 10.3389/fimmu.2024.1491808 (PMC11631915; doi:10.3389/fimmu.2024.1491808)
Supplement: Supplementary file 10 [file Table3.docx]

**Table S3: The differentially expressed anoikis-related genes (ARGs) in bladder cancer in the TCGA cohort.**

| gene | logFC | P value | FDR |
| --- | --- | --- | --- |
| \| ABL1 \| \| --- \| \| ADAMTSL1 \| \| AKT3 \| \| ANGPTL2 \| \| AR \| \| BAX \| \| BCL2 \| \| BCL2L1 \| \| BCL2L2 \| \| BDNF \| \| BID \| \| BIN1 \| \| BIRC5 \| \| BMF \| \| BRCA2 \| \| BRMS1 \| \| BST2 \| \| BUB1 \| \| CALR \| \| CASP2 \| \| CASP6 \| \| CAV1 \| \| CCDC178 \| \| CCDC80 \| \| CCN1 \| \| CCN2 \| \| CCN6 \| \| CD24 \| \| CDC25C \| \| CDCP1 \| \| CDH3 \| \| CDK1 \| \| CDKN1A \| \| CDKN2A \| \| CDKN3 \| \| CEMIP \| \| CHEK2 \| \| CLU \| \| CPEB2 \| \| CRYAB \| \| CRYBA1 \| \| CSPG4 \| \| CXCL12 \| \| CXCR4 \| \| E2F1 \| \| ELANE \| \| ERBB2 \| \| ETV4 \| \| EZH2 \| \| F10 \| \| FADD \| \| FASN \| \| FGF2 \| \| GKN1 \| \| GLI2 \| \| GRHL2 \| \| HMGA1 \| \| HMOX1 \| \| IFI27 \| \| IGF1 \| \| IL6 \| \| ILK \| \| ITGA3 \| \| ITGA5 \| \| ITGA8 \| \| ITGB3 \| \| ITGB4 \| \| ITPRIP \| \| KRT14 \| \| LAMC2 \| \| LMO3 \| \| LPAR1 \| \| MAD2L1 \| \| MET \| \| MIR145 \| \| MMP11 \| \| MMP13 \| \| MMP9 \| \| MNX1 \| \| MSLN \| \| MUC1 \| \| MYC \| \| NGF \| \| NKX2-1 \| \| NTF3 \| \| NTRK3 \| \| ONECUT1 \| \| PBK \| \| PCNA \| \| PDK4 \| \| PIK3R2 \| \| PLAU \| \| PLK1 \| \| PRDX4 \| \| PTGS2 \| \| PYCARD \| \| RAC3 \| \| RAD9A \| \| RHOB \| \| S100A7 \| \| SERPINA1 \| \| SFRP1 \| \| SIK1 \| \| SKP2 \| \| SPHK1 \| \| SPP1 \| \| TAGLN \| \| TFDP1 \| \| THBS1 \| \| TLN1 \| \| TP63 \| \| TP73 \| \| TPM1 \| \| TRAF2 \| \| TUBB3 \| \| UBE2C \| \| XAF1 \| \| ZEB1 \| \| ZEB2 \| | \| -1.333328098 \| \| --- \| \| -1.30910806 \| \| -1.568153745 \| \| -1.294299282 \| \| -1.068373464 \| \| 1.005870585 \| \| -1.29042716 \| \| 1.061039771 \| \| -1.155764176 \| \| -1.793329059 \| \| 1.346987413 \| \| -1.909972585 \| \| 2.089455787 \| \| 1.019198178 \| \| 1.964087871 \| \| 1.012630135 \| \| 1.136398148 \| \| 1.964898992 \| \| 1.19719996 \| \| 1.351295317 \| \| 1.192871038 \| \| -1.216743577 \| \| -2.122879827 \| \| -2.474150577 \| \| -2.742627642 \| \| -2.731396165 \| \| 1.333222066 \| \| 1.353878185 \| \| 2.345649746 \| \| 1.306670936 \| \| 2.800001922 \| \| 2.515800195 \| \| -1.339880731 \| \| 4.229752938 \| \| 1.806275312 \| \| 1.921908501 \| \| 1.038339179 \| \| -1.578541625 \| \| -1.514938742 \| \| -3.340580973 \| \| 1.37183265 \| \| -1.555514251 \| \| -1.783518072 \| \| -1.269925455 \| \| 2.001023648 \| \| -3.601037915 \| \| 1.440193646 \| \| 2.671079818 \| \| 1.963715056 \| \| -3.614808961 \| \| 1.409831656 \| \| 1.653621687 \| \| -2.42393496 \| \| -1.034568726 \| \| -1.159638698 \| \| 1.051545725 \| \| 1.30359533 \| \| 1.224461714 \| \| 2.202757353 \| \| -1.126783916 \| \| -3.225865284 \| \| -1.050283115 \| \| 1.231245125 \| \| -2.142704782 \| \| -3.212714836 \| \| -1.590946952 \| \| 1.069685326 \| \| -1.050686925 \| \| 5.242022448 \| \| 2.866660093 \| \| -3.368785854 \| \| -1.396350351 \| \| 1.955618906 \| \| 1.003957654 \| \| -3.508195439 \| \| 5.514908179 \| \| 3.899186325 \| \| 3.116508083 \| \| 3.429812499 \| \| 3.607267033 \| \| 1.610530182 \| \| -1.393397897 \| \| -1.128989227 \| \| 4.350728756 \| \| -2.26302284 \| \| -3.789468328 \| \| 3.266028491 \| \| 1.865233611 \| \| 1.489187867 \| \| -3.328261646 \| \| 1.09328167 \| \| 2.2680726 \| \| 2.39059031 \| \| 1.025074365 \| \| -2.084266513 \| \| 1.009989632 \| \| 2.869142509 \| \| 1.244365416 \| \| -1.927895972 \| \| 2.569374644 \| \| 2.85685674 \| \| -2.675494081 \| \| -1.46497923 \| \| 1.957975354 \| \| 1.5195971 \| \| 4.584874204 \| \| -2.991659774 \| \| 1.073429248 \| \| -2.350905711 \| \| -1.722658066 \| \| 1.012623776 \| \| 1.972370262 \| \| -3.183488958 \| \| 1.112883652 \| \| 2.411577984 \| \| 2.743434345 \| \| 1.30985987 \| \| -2.552201115 \| \| -1.67711826 \| | 5.86E-06  2.19E-07  5.81E-06  3.13E-05  5.17E-06  9.22E-09  1.08E-09  3.46E-07  1.09E-06  5.32E-09  8.96E-10  1.87E-08  8.82E-09  0.001782893  8.39E-09  1.53E-08  0.018119611  4.54E-09  1.36E-10  6.33E-11  4.70E-09  2.11E-05  7.53E-11  3.85E-08  3.49E-11  2.16E-09  0.018534633  0.012395563  4.27E-10  6.75E-05  2.10E-05  5.11E-11  0.000276282  0.002514958  6.96E-08  0.000369121  1.69E-07  1.48E-08  7.46E-09  1.94E-10  0.006329337  0.000219907  2.21E-09  0.002063942  1.97E-08  2.27E-12  0.005288862  9.02E-09  1.77E-09  7.37E-13  1.54E-07  8.43E-09  2.36E-10  0.02907322  3.95E-06  9.10E-05  6.70E-06  0.022702993  0.00384489  6.36E-07  2.97E-08  0.002610229  0.004155287  7.13E-06  2.47E-12  1.36E-05  0.000749037  3.84E-05  1.59E-05  5.80E-05  8.92E-09  8.55E-10  3.01E-09  0.004409335  7.34E-09  2.15E-11  0.000201126  0.002538534  4.09E-08  0.021709704  0.02867135  2.67E-05  6.06E-07  0.017235583  1.99E-10  1.10E-11  0.003677155  1.95E-07  8.62E-09  8.30E-12  0.00541267  7.19E-06  5.09E-10  3.07E-07  6.62E-07  0.000358668  3.63E-11  1.04E-07  3.07E-11  0.007284885  0.001078334  1.29E-09  0.000736316  1.20E-10  0.015614922  2.91E-08  6.16E-09  2.12E-07  2.52E-05  5.31E-05  0.009567008  0.000205685  6.95E-07  5.89E-09  1.35E-06  5.65E-11  0.026227554  2.12E-07  1.82E-08 | \| 2.30E-05 \| \| --- \| \| 1.20E-06 \| \| 2.30E-05 \| \| 0.000100783 \| \| 2.10E-05 \| \| 7.40E-08 \| \| 1.63E-08 \| \| 1.84E-06 \| \| 5.06E-06 \| \| 5.65E-08 \| \| 1.41E-08 \| \| 1.35E-07 \| \| 7.40E-08 \| \| 0.003763885 \| \| 7.40E-08 \| \| 1.18E-07 \| \| 0.029201694 \| \| 5.29E-08 \| \| 3.26E-09 \| \| 1.91E-09 \| \| 5.30E-08 \| \| 7.06E-05 \| \| 2.09E-09 \| \| 2.57E-07 \| \| 1.45E-09 \| \| 2.75E-08 \| \| 0.029606205 \| \| 0.021008442 \| \| 8.12E-09 \| \| 0.00020294 \| \| 7.06E-05 \| \| 1.84E-09 \| \| 0.000728013 \| \| 0.005145363 \| \| 4.41E-07 \| \| 0.0009384 \| \| 9.86E-07 \| \| 1.16E-07 \| \| 7.08E-08 \| \| 4.22E-09 \| \| 0.01165488 \| \| 0.000592437 \| \| 2.75E-08 \| \| 0.004282087 \| \| 1.40E-07 \| \| 2.98E-10 \| \| 0.009996227 \| \| 7.40E-08 \| \| 2.37E-08 \| \| 2.66E-10 \| \| 9.24E-07 \| \| 7.40E-08 \| \| 4.73E-09 \| \| 0.043549511 \| \| 1.66E-05 \| \| 0.000266997 \| \| 2.60E-05 \| \| 0.035479569 \| \| 0.007422488 \| \| 3.14E-06 \| \| 2.03E-07 \| \| 0.005234959 \| \| 0.007979034 \| \| 2.73E-05 \| \| 2.98E-10 \| \| 4.85E-05 \| \| 0.001711407 \| \| 0.000121581 \| \| 5.47E-05 \| \| 0.000177394 \| \| 7.40E-08 \| \| 1.40E-08 \| \| 3.62E-08 \| \| 0.008377737 \| \| 7.08E-08 \| \| 1.29E-09 \| \| 0.000550048 \| \| 0.005148375 \| \| 2.69E-07 \| \| 0.034223594 \| \| 0.043126489 \| \| 8.77E-05 \| \| 3.04E-06 \| \| 0.028027232 \| \| 4.22E-09 \| \| 7.97E-10 \| \| 0.007175422 \| \| 1.12E-06 \| \| 7.40E-08 \| \| 7.49E-10 \| \| 0.010176948 \| \| 2.73E-05 \| \| 9.18E-09 \| \| 1.65E-06 \| \| 3.23E-06 \| \| 0.000918292 \| \| 1.45E-09 \| \| 6.50E-07 \| \| 1.45E-09 \| \| 0.013024104 \| \| 0.002388213 \| \| 1.86E-08 \| \| 0.001693057 \| \| 3.09E-09 \| \| 0.025622667 \| \| 2.02E-07 \| \| 6.18E-08 \| \| 1.18E-06 \| \| 8.35E-05 \| \| 0.000165222 \| \| 0.016765485 \| \| 0.000558287 \| \| 3.35E-06 \| \| 6.08E-08 \| \| 6.03E-06 \| \| 1.86E-09 \| \| 0.039615678 \| \| 1.18E-06 \| \| 1.35E-07 \| |
