# Supplementary material for: Identification of anoikis-related molecular patterns and the novel risk model to predict prognosis, tumor microenvironment infiltration and immunotherapy response in bladder cancer
Source: Front Immunol. 2024 Nov 27;15:1491808. doi: 10.3389/fimmu.2024.1491808 (PMC11631915; doi:10.3389/fimmu.2024.1491808)
Supplement: Supplementary file 11 [file Table4.docx]

**Table S4: 404 bladder cancer patients in the TCGA were classified into ARGcluster A (n = 213) and ARGcluster B (n = 191).**

| Sample | ARGcluster |
| --- | --- |
| \| TCGA-ZF-A9RN \| \| --- \| \| TCGA-CF-A1HR \| \| TCGA-XF-A8HH \| \| TCGA-ZF-AA4W \| \| TCGA-XF-A9SU \| \| TCGA-DK-A3IQ \| \| TCGA-FD-A6TK \| \| TCGA-DK-A1A7 \| \| TCGA-CU-A5W6 \| \| TCGA-DK-A3X1 \| \| TCGA-XF-A9ST \| \| TCGA-CF-A9FM \| \| TCGA-BT-A20V \| \| TCGA-GV-A3QF \| \| TCGA-DK-A2I4 \| \| TCGA-DK-A6B2 \| \| TCGA-DK-A1AG \| \| TCGA-XF-A9SZ \| \| TCGA-E7-A519 \| \| TCGA-2F-A9KO \| \| TCGA-ZF-A9R1 \| \| TCGA-4Z-AA7S \| \| TCGA-BL-A13J \| \| TCGA-DK-A3IK \| \| TCGA-C4-A0F1 \| \| TCGA-DK-A3IS \| \| TCGA-UY-A9PH \| \| TCGA-E5-A4TZ \| \| TCGA-DK-A3WW \| \| TCGA-K4-A5RH \| \| TCGA-ZF-AA56 \| \| TCGA-UY-A8OB \| \| TCGA-YC-A9TC \| \| TCGA-CF-A7I0 \| \| TCGA-K4-A5RJ \| \| TCGA-YF-AA3L \| \| TCGA-CF-A3MI \| \| TCGA-DK-A6B0 \| \| TCGA-DK-A6B1 \| \| TCGA-G2-AA3C \| \| TCGA-XF-AAN3 \| \| TCGA-CU-A0YO \| \| TCGA-BT-A3PJ \| \| TCGA-CF-A47Y \| \| TCGA-FD-A62O \| \| TCGA-BL-A0C8 \| \| TCGA-E7-A7DV \| \| TCGA-DK-A3WY \| \| TCGA-BT-A20N \| \| TCGA-G2-A2ES \| \| TCGA-GC-A3YS \| \| TCGA-FD-A43X \| \| TCGA-XF-A9SM \| \| TCGA-DK-A6AV \| \| TCGA-GU-A42Q \| \| TCGA-C4-A0F7 \| \| TCGA-CF-A47W \| \| TCGA-XF-AAMQ \| \| TCGA-K4-A3WS \| \| TCGA-C4-A0EZ \| \| TCGA-CF-A47S \| \| TCGA-ZF-AA5P \| \| TCGA-XF-A9SX \| \| TCGA-FD-A3B8 \| \| TCGA-CF-A27C \| \| TCGA-XF-A9T4 \| \| TCGA-HQ-A2OF \| \| TCGA-DK-AA74 \| \| TCGA-FD-A3SM \| \| TCGA-KQ-A41P \| \| TCGA-C4-A0F6 \| \| TCGA-FD-A3B5 \| \| TCGA-4Z-AA80 \| \| TCGA-FD-A3B6 \| \| TCGA-4Z-AA87 \| \| TCGA-DK-A1A6 \| \| TCGA-DK-A1AD \| \| TCGA-FD-A5BS \| \| TCGA-2F-A9KW \| \| TCGA-DK-AA6T \| \| TCGA-4Z-AA84 \| \| TCGA-FJ-A3Z9 \| \| TCGA-CU-A0YR \| \| TCGA-FD-A3B3 \| \| TCGA-FD-A43N \| \| TCGA-BL-A13I \| \| TCGA-KQ-A41S \| \| TCGA-4Z-AA86 \| \| TCGA-E7-A5KF \| \| TCGA-XF-AAN7 \| \| TCGA-DK-AA6M \| \| TCGA-CU-A3YL \| \| TCGA-ZF-A9RD \| \| TCGA-C4-A0F0 \| \| TCGA-FD-A3SR \| \| TCGA-DK-A6B5 \| \| TCGA-HQ-A5ND \| \| TCGA-DK-AA76 \| \| TCGA-CF-A47V \| \| TCGA-E7-A3Y1 \| \| TCGA-ZF-AA53 \| \| TCGA-ZF-A9R7 \| \| TCGA-GV-A40G \| \| TCGA-4Z-AA7M \| \| TCGA-BT-A2LD \| \| TCGA-4Z-AA82 \| \| TCGA-FD-A62P \| \| TCGA-DK-AA6U \| \| TCGA-FD-A3N6 \| \| TCGA-2F-A9KR \| \| TCGA-CF-A3MF \| \| TCGA-CU-A3KJ \| \| TCGA-FD-A6TC \| \| TCGA-XF-A8HB \| \| TCGA-XF-AAN1 \| \| TCGA-FJ-A871 \| \| TCGA-UY-A9PA \| \| TCGA-E7-A4IJ \| \| TCGA-XF-AAMZ \| \| TCGA-XF-AAMR \| \| TCGA-BT-A42E \| \| TCGA-K4-A6FZ \| \| TCGA-DK-A1AF \| \| TCGA-FD-A6TB \| \| TCGA-GU-AATO \| \| TCGA-BT-A20U \| \| TCGA-DK-A1AA \| \| TCGA-GV-A3JW \| \| TCGA-FD-A5BZ \| \| TCGA-ZF-A9R9 \| \| TCGA-G2-AA3F \| \| TCGA-ZF-AA5H \| \| TCGA-XF-A8HD \| \| TCGA-FD-A6TH \| \| TCGA-4Z-AA7O \| \| TCGA-DK-A6B6 \| \| TCGA-E7-A6MF \| \| TCGA-DK-AA6W \| \| TCGA-GC-A3BM \| \| TCGA-GU-A42P \| \| TCGA-GV-A3QI \| \| TCGA-XF-A9T3 \| \| TCGA-E7-A8O7 \| \| TCGA-DK-AA77 \| \| TCGA-XF-AAML \| \| TCGA-2F-A9KQ \| \| TCGA-CF-A3MH \| \| TCGA-XF-A8HC \| \| TCGA-ZF-AA4T \| \| TCGA-FD-A6TI \| \| TCGA-H4-A2HO \| \| TCGA-CF-A47X \| \| TCGA-XF-A9SP \| \| TCGA-K4-A4AC \| \| TCGA-BL-A5ZZ \| \| TCGA-S5-AA26 \| \| TCGA-E7-A677 \| \| TCGA-DK-A1AC \| \| TCGA-GU-A766 \| \| TCGA-DK-A2HX \| \| TCGA-UY-A8OC \| \| TCGA-FD-A5BR \| \| TCGA-DK-AA6S \| \| TCGA-4Z-AA7Q \| \| TCGA-HQ-A2OE \| \| TCGA-XF-AAMX \| \| TCGA-LT-A5Z6 \| \| TCGA-YC-A89H \| \| TCGA-E7-A8O8 \| \| TCGA-GC-A3OO \| \| TCGA-DK-AA75 \| \| TCGA-BT-A2LB \| \| TCGA-ZF-AA54 \| \| TCGA-FT-A3EE \| \| TCGA-FJ-A3ZF \| \| TCGA-DK-A3X2 \| \| TCGA-XF-AAME \| \| TCGA-ZF-AA4R \| \| TCGA-GV-A3JZ \| \| TCGA-DK-A2I2 \| \| TCGA-K4-A83P \| \| TCGA-4Z-AA7Y \| \| TCGA-E7-A7XN \| \| TCGA-2F-A9KP \| \| TCGA-UY-A78M \| \| TCGA-FD-A6TD \| \| TCGA-BT-A20Q \| \| TCGA-ZF-AA51 \| \| TCGA-GU-A42R \| \| TCGA-PQ-A6FI \| \| TCGA-ZF-AA4X \| \| TCGA-FD-A5BU \| \| TCGA-CF-A8HX \| \| TCGA-XF-A9SK \| \| TCGA-GD-A3OS \| \| TCGA-FD-A6TG \| \| TCGA-4Z-AA7N \| \| TCGA-GV-A3QK \| \| TCGA-K4-A5RI \| \| TCGA-GV-A40E \| \| TCGA-FD-A43Y \| \| TCGA-DK-A3IV \| \| TCGA-G2-A2EL \| \| TCGA-E7-A541 \| \| TCGA-XF-AAN2 \| \| TCGA-LC-A66R \| \| TCGA-ZF-A9RE \| \| TCGA-UY-A9PE \| \| TCGA-XF-AAMG \| \| TCGA-GU-A763 \| \| TCGA-G2-AA3D \| \| TCGA-ZF-A9R4 \| \| TCGA-E7-A97P \| \| TCGA-5N-A9KM \| \| TCGA-ZF-A9RC \| \| TCGA-MV-A51V \| \| TCGA-S5-A6DX \| \| TCGA-FJ-A3ZE \| \| TCGA-YC-A8S6 \| \| TCGA-UY-A9PF \| \| TCGA-GV-A3JV \| \| TCGA-ZF-AA5N \| \| TCGA-XF-A9T8 \| \| TCGA-5N-A9KI \| \| TCGA-CF-A3MG \| \| TCGA-GC-A4ZW \| \| TCGA-E7-A4XJ \| \| TCGA-FD-A3NA \| \| TCGA-DK-A3IT \| \| TCGA-R3-A69X \| \| TCGA-4Z-AA83 \| \| TCGA-ZF-A9R0 \| \| TCGA-GC-A3I6 \| \| TCGA-XF-A9SH \| \| TCGA-UY-A78L \| \| TCGA-XF-AAN5 \| \| TCGA-UY-A78P \| \| TCGA-CF-A8HY \| \| TCGA-CF-A9FL \| \| TCGA-E7-A6MD \| \| TCGA-CF-A9FF \| \| TCGA-XF-AAMY \| \| TCGA-G2-A3IE \| \| TCGA-GU-A767 \| \| TCGA-G2-A3IB \| \| TCGA-CF-A1HS \| \| TCGA-DK-A3IN \| \| TCGA-2F-A9KT \| \| TCGA-UY-A78N \| \| TCGA-DK-A1A5 \| \| TCGA-E7-A5KE \| \| TCGA-FD-A62S \| \| TCGA-G2-AA3B \| \| TCGA-K4-A54R \| \| TCGA-XF-A9T5 \| \| TCGA-BT-A20J \| \| TCGA-XF-AAMW \| \| TCGA-ZF-AA4V \| \| TCGA-GU-AATP \| \| TCGA-CU-A0YN \| \| TCGA-XF-AAMH \| \| TCGA-DK-A2I6 \| \| TCGA-DK-AA6Q \| \| TCGA-XF-A8HI \| \| TCGA-DK-A2I1 \| \| TCGA-E7-A7PW \| \| TCGA-XF-AAMT \| \| TCGA-BT-A20R \| \| TCGA-H4-A2HQ \| \| TCGA-FD-A43S \| \| TCGA-ZF-AA58 \| \| TCGA-DK-AA6P \| \| TCGA-XF-AAN0 \| \| TCGA-BT-A0YX \| \| TCGA-ZF-A9R5 \| \| TCGA-GV-A3JX \| \| TCGA-ZF-A9R3 \| \| TCGA-G2-A2EO \| \| TCGA-CF-A5UA \| \| TCGA-K4-A3WU \| \| TCGA-DK-AA71 \| \| TCGA-XF-A9T2 \| \| TCGA-FD-A5BY \| \| TCGA-BT-A20T \| \| TCGA-UY-A78O \| \| TCGA-4Z-AA89 \| \| TCGA-XF-A9SW \| \| TCGA-BT-A20W \| \| TCGA-FD-A3N5 \| \| TCGA-DK-A3WX \| \| TCGA-FD-A62N \| \| TCGA-HQ-A5NE \| \| TCGA-XF-A8HE \| \| TCGA-DK-AA6R \| \| TCGA-XF-A9SI \| \| TCGA-BT-A42C \| \| TCGA-KQ-A41O \| \| TCGA-YF-AA3M \| \| TCGA-ZF-AA4N \| \| TCGA-G2-A2EC \| \| TCGA-CF-A5U8 \| \| TCGA-UY-A8OD \| \| TCGA-4Z-AA7R \| \| TCGA-FD-A3B4 \| \| TCGA-FJ-A3Z7 \| \| TCGA-G2-A2EJ \| \| TCGA-SY-A9G0 \| \| TCGA-G2-A2EK \| \| TCGA-FD-A5BX \| \| TCGA-XF-AAN4 \| \| TCGA-FD-A3SO \| \| TCGA-FD-A6TA \| \| TCGA-FD-A3SS \| \| TCGA-FD-A43P \| \| TCGA-BT-A3PK \| \| TCGA-XF-A9T6 \| \| TCGA-FD-A5BV \| \| TCGA-ZF-AA4U \| \| TCGA-SY-A9G5 \| \| TCGA-KQ-A41R \| \| TCGA-GC-A6I1 \| \| TCGA-GU-A764 \| \| TCGA-G2-A2EF \| \| TCGA-FT-A61P \| \| TCGA-K4-AAQO \| \| TCGA-DK-AA6L \| \| TCGA-K4-A6MB \| \| TCGA-BT-A20P \| \| TCGA-FD-A3SJ \| \| TCGA-G2-A3VY \| \| TCGA-GC-A3WC \| \| TCGA-CU-A3QU \| \| TCGA-FD-A3SP \| \| TCGA-GC-A3RB \| \| TCGA-GU-AATQ \| \| TCGA-GC-A6I3 \| \| TCGA-GC-A3RD \| \| TCGA-GD-A3OQ \| \| TCGA-CF-A47T \| \| TCGA-ZF-AA52 \| \| TCGA-XF-A8HG \| \| TCGA-FD-A3B7 \| \| TCGA-DK-A3IL \| \| TCGA-E7-A6ME \| \| TCGA-GD-A2C5 \| \| TCGA-DK-AA6X \| \| TCGA-UY-A9PD \| \| TCGA-DK-A3IM \| \| TCGA-BT-A42F \| \| TCGA-FD-A3SL \| \| TCGA-BT-A3PH \| \| TCGA-FD-A3SQ \| \| TCGA-ZF-A9RF \| \| TCGA-DK-A1A3 \| \| TCGA-BT-A2LA \| \| TCGA-PQ-A6FN \| \| TCGA-GD-A3OP \| \| TCGA-FD-A5BT \| \| TCGA-BL-A3JM \| \| TCGA-E7-A85H \| \| TCGA-CU-A72E \| \| TCGA-FD-A3SN \| \| TCGA-GU-A762 \| \| TCGA-GV-A3QH \| \| TCGA-FD-A6TF \| \| TCGA-XF-A9SJ \| \| TCGA-ZF-A9RL \| \| TCGA-E7-A678 \| \| TCGA-GD-A76B \| \| TCGA-GV-A6ZA \| \| TCGA-FD-A5C1 \| \| TCGA-BT-A20X \| \| TCGA-K4-A3WV \| \| TCGA-FD-A5C0 \| \| TCGA-DK-A3IU \| \| TCGA-UY-A78K \| \| TCGA-DK-A1AE \| \| TCGA-LT-A8JT \| \| TCGA-BT-A20O \| \| TCGA-K4-A4AB \| \| TCGA-FD-A43U \| \| TCGA-FD-A6TE \| \| TCGA-XF-A9SV \| \| TCGA-GD-A6C6 \| \| TCGA-KQ-A41N \| \| TCGA-4Z-AA7W \| \| TCGA-KQ-A41Q \| \| TCGA-E7-A3X6 \| \| TCGA-XF-A9SL \| \| TCGA-4Z-AA81 \| \| TCGA-DK-A1AB \| \| TCGA-XF-A9T0 \| \| TCGA-GC-A3RC \| \| TCGA-XF-AAN8 \| \| TCGA-XF-A8HF \| \| TCGA-E5-A2PC \| \| TCGA-ZF-A9R2 \| \| TCGA-E5-A4U1 \| \| TCGA-BT-A0S7 \| \| TCGA-ZF-A9RM \| \| TCGA-UY-A9PB \| \| TCGA-XF-A9SY \| \| TCGA-E7-A7DU \| \| TCGA-XF-AAMJ \| | \| A \| \| --- \| \| A \| \| B \| \| B \| \| B \| \| B \| \| B \| \| A \| \| A \| \| A \| \| A \| \| A \| \| A \| \| A \| \| B \| \| B \| \| A \| \| B \| \| A \| \| B \| \| A \| \| A \| \| B \| \| A \| \| B \| \| A \| \| A \| \| A \| \| B \| \| B \| \| B \| \| B \| \| A \| \| A \| \| A \| \| A \| \| A \| \| A \| \| A \| \| B \| \| B \| \| B \| \| B \| \| A \| \| A \| \| A \| \| B \| \| B \| \| A \| \| B \| \| B \| \| A \| \| B \| \| A \| \| B \| \| B \| \| A \| \| A \| \| B \| \| A \| \| A \| \| B \| \| B \| \| B \| \| A \| \| B \| \| A \| \| B \| \| B \| \| A \| \| A \| \| B \| \| A \| \| A \| \| A \| \| A \| \| A \| \| B \| \| B \| \| A \| \| A \| \| A \| \| B \| \| B \| \| A \| \| B \| \| B \| \| B \| \| A \| \| B \| \| B \| \| A \| \| B \| \| B \| \| B \| \| A \| \| A \| \| A \| \| A \| \| A \| \| B \| \| A \| \| A \| \| A \| \| B \| \| B \| \| B \| \| A \| \| A \| \| A \| \| A \| \| A \| \| B \| \| A \| \| A \| \| B \| \| A \| \| A \| \| A \| \| B \| \| A \| \| B \| \| B \| \| B \| \| B \| \| B \| \| A \| \| A \| \| B \| \| B \| \| A \| \| B \| \| B \| \| B \| \| A \| \| A \| \| A \| \| A \| \| A \| \| A \| \| A \| \| B \| \| A \| \| A \| \| A \| \| A \| \| A \| \| A \| \| A \| \| A \| \| A \| \| A \| \| B \| \| B \| \| B \| \| A \| \| A \| \| A \| \| B \| \| B \| \| B \| \| B \| \| B \| \| A \| \| A \| \| A \| \| A \| \| A \| \| A \| \| B \| \| A \| \| B \| \| B \| \| A \| \| A \| \| A \| \| B \| \| B \| \| A \| \| B \| \| B \| \| A \| \| A \| \| A \| \| B \| \| B \| \| B \| \| A \| \| A \| \| A \| \| A \| \| B \| \| A \| \| B \| \| B \| \| B \| \| B \| \| A \| \| B \| \| B \| \| B \| \| A \| \| A \| \| A \| \| A \| \| B \| \| A \| \| A \| \| B \| \| A \| \| A \| \| A \| \| B \| \| A \| \| B \| \| A \| \| B \| \| A \| \| B \| \| A \| \| B \| \| A \| \| B \| \| B \| \| A \| \| A \| \| A \| \| B \| \| B \| \| B \| \| A \| \| B \| \| B \| \| A \| \| B \| \| A \| \| B \| \| A \| \| A \| \| B \| \| A \| \| B \| \| A \| \| B \| \| B \| \| A \| \| B \| \| A \| \| A \| \| B \| \| A \| \| B \| \| A \| \| B \| \| A \| \| B \| \| B \| \| B \| \| B \| \| B \| \| A \| \| A \| \| A \| \| A \| \| B \| \| A \| \| B \| \| B \| \| A \| \| B \| \| B \| \| A \| \| B \| \| B \| \| A \| \| A \| \| A \| \| B \| \| A \| \| B \| \| A \| \| B \| \| B \| \| B \| \| A \| \| A \| \| B \| \| A \| \| B \| \| B \| \| B \| \| B \| \| A \| \| B \| \| B \| \| A \| \| A \| \| A \| \| B \| \| A \| \| A \| \| B \| \| A \| \| B \| \| B \| \| B \| \| B \| \| A \| \| B \| \| B \| \| B \| \| B \| \| B \| \| A \| \| B \| \| A \| \| A \| \| A \| \| B \| \| A \| \| B \| \| B \| \| B \| \| B \| \| B \| \| A \| \| A \| \| A \| \| B \| \| A \| \| B \| \| A \| \| B \| \| A \| \| A \| \| A \| \| A \| \| B \| \| A \| \| B \| \| A \| \| B \| \| A \| \| A \| \| A \| \| A \| \| B \| \| B \| \| A \| \| B \| \| A \| \| B \| \| A \| \| B \| \| A \| \| B \| \| A \| \| B \| \| B \| \| A \| \| B \| \| B \| \| B \| \| A \| \| B \| \| B \| \| A \| \| A \| \| A \| \| A \| \| B \| \| B \| \| A \| \| B \| \| B \| \| A \| \| A \| \| A \| \| B \| \| B \| \| B \| \| A \| \| B \| \| A \| \| A \| \| A \| \| A \| \| A \| \| B \| \| A \| \| B \| \| A \| \| B \| \| B \| \| A \| \| A \| \| A \| \| A \| \| B \| \| A \| \| B \| \| B \| \| A \| \| B \| |
