# Supplementary material for: Identification of anoikis-related molecular patterns and the novel risk model to predict prognosis, tumor microenvironment infiltration and immunotherapy response in bladder cancer
Source: Front Immunol. 2024 Nov 27;15:1491808. doi: 10.3389/fimmu.2024.1491808 (PMC11631915; doi:10.3389/fimmu.2024.1491808)
Supplement: Supplementary file 12 [file Table5.docx]

**Table S5. Identification of anoikis phenotype-associated differentially expressed genes (DEGs).**

| gene | logFC | P value | FDR |
| --- | --- | --- | --- |
| \| MRC1 \| \| --- \| \| IBSP \| \| IL1RL1 \| \| AOX1 \| \| DSC1 \| \| IGLL5 \| \| GAS6 \| \| CHRNB2 \| \| CCL25 \| \| NEFL \| \| SERPINA9 \| \| TWIST2 \| \| PRRX1 \| \| CRTAC1 \| \| C20orf141 \| \| ADGRD1 \| \| ERN2 \| \| MT1A \| \| SPINK4 \| \| CALD1 \| \| CCDC80 \| \| KCNH4 \| \| MEDAG \| \| COL3A1 \| \| P2RX1 \| \| IL7R \| \| KRT84 \| \| WNT2 \| \| BPIFA2 \| \| OMD \| \| CTSE \| \| SYNDIG1 \| \| SFRP1 \| \| ISLR \| \| FILIP1L \| \| ARSI \| \| HSPB3 \| \| CHRDL2 \| \| GXYLT2 \| \| SPINK6 \| \| ADRB3 \| \| FPR1 \| \| CXCL5 \| \| TPM2 \| \| CEBPE \| \| HEPACAM2 \| \| SYNC \| \| MRO \| \| ITGB3 \| \| CYP4F12 \| \| MCEMP1 \| \| ADAMTS2 \| \| HAND2 \| \| SEZ6 \| \| FDCSP \| \| FPR2 \| \| SPRR2A \| \| PHGR1 \| \| NAV3 \| \| IGF1 \| \| HMGCS2 \| \| PDCD1LG2 \| \| MYL9 \| \| ADIPOQ \| \| KCNMB1 \| \| FIBIN \| \| RAMP1 \| \| TNIP3 \| \| SLC39A5 \| \| SPRR2F \| \| CXCL12 \| \| HS6ST3 \| \| LAMC2 \| \| MYO15A \| \| CR1 \| \| CCL23 \| \| SNTG1 \| \| IHH \| \| SORBS1 \| \| SMOC2 \| \| PTGIS \| \| HEPHL1 \| \| FSIP2 \| \| SGCD \| \| SMIM31 \| \| ANGPTL1 \| \| CDA \| \| KLK7 \| \| CASQ2 \| \| CYP1A2 \| \| MMP9 \| \| SYNPO2 \| \| SCARF2 \| \| ASPN \| \| GATA5 \| \| TCHHL1 \| \| ANXA10 \| \| KLHL4 \| \| IL37 \| \| CD300C \| \| FGF5 \| \| GFPT2 \| \| GAS1 \| \| LOX \| \| SCRG1 \| \| NTSR1 \| \| GPC3 \| \| SULF1 \| \| LRRC15 \| \| KRT34 \| \| TRH \| \| ADAMTS12 \| \| SERTM2 \| \| PLA2G5 \| \| MFAP5 \| \| ASB5 \| \| BAAT \| \| PRUNE2 \| \| ACTG2 \| \| ANPEP \| \| VCAN \| \| CTHRC1 \| \| LRRC19 \| \| ATP1A2 \| \| VSIG4 \| \| FN1 \| \| CCIN \| \| TRIML2 \| \| P4HA3 \| \| MRGPRF \| \| LRRC38 \| \| IL6 \| \| CYP2C9 \| \| ERVW-1 \| \| COL9A1 \| \| GPR68 \| \| NNMT \| \| COL8A2 \| \| ROS1 \| \| ATP6V0D2 \| \| COMP \| \| MSRB3 \| \| RUBCNL \| \| FLNC \| \| GZMA \| \| HMGA2 \| \| TPSD1 \| \| EMP3 \| \| PACRG \| \| BHMT2 \| \| RBFOX3 \| \| CD209 \| \| ODAPH \| \| NNAT \| \| CD300E \| \| LILRA6 \| \| TBX4 \| \| UGT2A3 \| \| CLC \| \| GALNT15 \| \| G0S2 \| \| POPDC2 \| \| HSPB6 \| \| GRM3 \| \| SPARC \| \| FCGR3A \| \| IL24 \| \| ONECUT2 \| \| SUGCT \| \| SCARA5 \| \| CHI3L1 \| \| GEM \| \| ANGPTL7 \| \| DES \| \| CYP4F8 \| \| MYH11 \| \| F13A1 \| \| TAGLN \| \| ADGRE3 \| \| CHRDL1 \| \| GREM1 \| \| CXCL13 \| \| CTSG \| \| SHISAL1 \| \| SCGB2A1 \| \| KSR2 \| \| COL16A1 \| \| TNS1 \| \| NLRP3 \| \| HAND1 \| \| CYP4F2 \| \| LAMA3 \| \| RFX8 \| \| ECRG4 \| \| KCNE4 \| \| COL11A1 \| \| SLC10A1 \| \| TCAP \| \| SPON2 \| \| ADAMTS14 \| \| RTL1 \| \| CCL21 \| \| NPHS2 \| \| CMKLR1 \| \| INSYN2B \| \| SPINK14 \| \| MYLK \| \| SFRP2 \| \| ENPP1 \| \| AARD \| \| MMP3 \| \| CSF1R \| \| LCE1E \| \| CRH \| \| MRGPRX3 \| \| SLC47A2 \| \| ETNPPL \| \| KRT85 \| \| BOC \| \| LMCD1 \| \| FCAR \| \| VIM \| \| PDZRN4 \| \| ACTL6B \| \| PCP4 \| \| TCL1A \| \| CCL24 \| \| ITGBL1 \| \| SLC13A2 \| \| LILRB5 \| \| IL31RA \| \| COL5A1 \| \| NLRP7 \| \| TFF1 \| \| ZNF469 \| \| PLN \| \| POSTN \| \| XIRP1 \| \| NRP2 \| \| ASIC5 \| \| GPC6 \| \| GLP2R \| \| CPXM1 \| \| RSPO2 \| \| BNC1 \| \| ALPK2 \| \| ACSM6 \| \| COL1A1 \| \| OGN \| \| LYVE1 \| \| CTNND2 \| \| SPP1 \| \| EPYC \| \| GABRP \| \| COL6A3 \| \| SYNM \| \| FMO1 \| \| BNC2 \| \| CDH11 \| \| XPNPEP2 \| \| FAM180A \| \| GLT8D2 \| \| CAMK2A \| \| PPBP \| \| CD248 \| \| MARCO \| \| CCER2 \| \| PLPP4 \| \| CR2 \| \| CTSK \| \| CCL7 \| \| COL6A1 \| \| PTGS1 \| \| CSDC2 \| \| KIF1A \| \| DDR2 \| \| KRT14 \| \| SH2D5 \| \| CLSTN2 \| \| HS3ST3A1 \| \| CD163 \| \| LPA \| \| ITGA5 \| \| MAMDC2 \| \| COLEC12 \| \| ADAM33 \| \| CILP \| \| CRYAB \| \| ROR2 \| \| CMA1 \| \| PDGFRB \| \| AC019117.1 \| \| MYBPH \| \| LILRB2 \| \| MFAP4 \| \| DCN \| \| SPHK1 \| \| PDPN \| \| TENM2 \| \| ADAM12 \| \| MXRA8 \| \| PI16 \| \| GPR1 \| \| F3 \| \| IL1R2 \| \| COL5A3 \| \| FAP \| \| KRT6B \| \| TGFB3 \| \| RSPO3 \| \| AHNAK2 \| \| FAM20C \| \| PDLIM3 \| \| UGT2B15 \| \| SPRR2D \| \| PSD \| \| CCL19 \| \| CIDEC \| \| ACTBL2 \| \| FNDC1 \| \| CYBB \| \| MT2A \| \| PI3 \| \| DPT \| \| AXL \| \| KIAA1755 \| \| BTBD16 \| \| KANK4 \| \| CXCR1 \| \| FGFBP2 \| \| GDF5 \| \| CELF3 \| \| CYTL1 \| \| DCSTAMP \| \| COL10A1 \| \| VWA5B2 \| \| FIBCD1 \| \| TLR8 \| \| LRTM1 \| \| FOLR3 \| \| SSTR5 \| \| KRT75 \| \| NEXN \| \| PTGFR \| \| SLC2A4 \| \| NOG \| \| FOXC2 \| \| KLHL38 \| \| FGF7 \| \| PSG4 \| \| RANBP3L \| \| FHL1 \| \| MMP11 \| \| CAVIN1 \| \| OLFML3 \| \| PODNL1 \| \| C6orf15 \| \| UGT2B28 \| \| SGCA \| \| HAS1 \| \| COL6A2 \| \| TTR \| \| BGN \| \| KLK5 \| \| SRPX \| \| CPA3 \| \| CSF3 \| \| CHRM2 \| \| S100A7A \| \| PPY \| \| KRTAP5-10 \| \| TRARG1 \| \| FBN1 \| \| IGFL3 \| \| REG4 \| \| AEBP1 \| \| TNXB \| \| EMILIN1 \| \| LGALS4 \| \| JPH2 \| \| CD70 \| \| C7 \| \| KCNH6 \| \| ART4 \| \| ACTA2 \| \| NLRP10 \| \| HPSE2 \| \| COL1A2 \| \| MEGF10 \| \| FCAMR \| \| C4B \| \| TACR2 \| \| CCN1 \| \| LEFTY2 \| \| PTX3 \| \| DACT3 \| \| RARRES2 \| \| ACTC1 \| \| CPXM2 \| \| ATOH8 \| \| KRT86 \| \| LILRA5 \| \| MCHR1 \| \| MMP2 \| \| MKX \| \| CCL8 \| \| ITGA11 \| \| GSTA2 \| \| APCDD1L \| \| FOLR2 \| \| CCN2 \| \| HSPB7 \| \| ADAM19 \| \| SPIB \| \| SULT1A2 \| \| LMOD1 \| \| LUM \| \| ANKRD1 \| \| ADAMTS16 \| \| MUC2 \| \| LEAP2 \| \| CCN4 \| \| PGM5 \| \| MN1 \| \| INHBA \| \| KRT33A \| \| TNC \| \| COL5A2 \| \| MT1M \| \| IGFL2 \| \| TNFAIP6 \| \| RPE65 \| \| CNN1 \| \| EVPLL \| \| TIMP2 \| \| TLL1 \| \| KRT81 \| \| SSC5D \| \| DPEP3 \| \| TGFBI \| \| CCN5 \| \| FOXN4 \| \| HTRA3 \| \| SFRP4 \| \| CLEC4D \| \| PRELP \| | \| 2.075619505 \| \| --- \| \| 3.430648373 \| \| 2.379346964 \| \| 2.187268739 \| \| 2.403951012 \| \| 2.139207947 \| \| 2.060657299 \| \| -2.418040575 \| \| 2.358668776 \| \| 3.949311072 \| \| 2.658386948 \| \| 2.639675717 \| \| 2.499950609 \| \| -3.848514812 \| \| 3.553350602 \| \| 2.216249614 \| \| -2.012409856 \| \| 2.828140545 \| \| -3.676160042 \| \| 2.100142536 \| \| 3.012688733 \| \| -2.061570771 \| \| 2.985837793 \| \| 2.670526521 \| \| 3.339070059 \| \| 2.007308993 \| \| 2.783020187 \| \| 2.045446376 \| \| -2.903315786 \| \| 3.247470287 \| \| -2.371860764 \| \| 2.984641083 \| \| 2.104480738 \| \| 3.006208224 \| \| 2.014946446 \| \| 2.669337915 \| \| 2.376957781 \| \| 3.56587129 \| \| 2.210152189 \| \| 2.197736521 \| \| 3.171299223 \| \| 2.408171 \| \| 2.449534479 \| \| 2.214069161 \| \| 2.178516825 \| \| -3.071718569 \| \| 2.112729496 \| \| 2.101897952 \| \| 2.071024757 \| \| -2.211395325 \| \| 2.007891618 \| \| 2.586304052 \| \| 2.412462457 \| \| -3.99751073 \| \| 5.62442025 \| \| 2.302618503 \| \| 2.035062245 \| \| -2.044747037 \| \| 2.347353644 \| \| 2.831572089 \| \| -2.545736084 \| \| 2.253930959 \| \| 2.455244379 \| \| 2.423956368 \| \| 2.659237418 \| \| 2.69174 \| \| 2.474770125 \| \| 2.016098463 \| \| -2.153702105 \| \| 2.012321158 \| \| 2.413981292 \| \| -2.280686155 \| \| 2.3742568 \| \| -2.260203589 \| \| 2.308606236 \| \| 2.200522612 \| \| -2.487733502 \| \| -3.635479638 \| \| 2.573961595 \| \| 2.050616572 \| \| 2.289087659 \| \| 2.345994665 \| \| -2.017869781 \| \| 2.669327918 \| \| -2.096716088 \| \| 3.046836542 \| \| 2.111388494 \| \| 3.440519731 \| \| 3.630099044 \| \| -3.352495517 \| \| 3.65425393 \| \| 2.894090482 \| \| 2.128685934 \| \| 2.983237535 \| \| 3.002406858 \| \| 2.588220866 \| \| -2.186711131 \| \| 2.205921255 \| \| -2.682016432 \| \| 2.078749939 \| \| 3.687832534 \| \| 2.937071259 \| \| 2.605135902 \| \| 2.193596248 \| \| 3.497898393 \| \| 3.342222239 \| \| -2.460193574 \| \| 2.679646668 \| \| 3.759254049 \| \| 3.053233408 \| \| 3.622298839 \| \| 2.530515109 \| \| 3.375264231 \| \| 2.786412108 \| \| 3.144604562 \| \| 4.110073452 \| \| -3.579388681 \| \| 2.454853535 \| \| 3.531004512 \| \| 2.073067989 \| \| 2.03004019 \| \| 2.444443308 \| \| -2.109426246 \| \| 3.806998749 \| \| 2.303737824 \| \| 2.769863398 \| \| 2.527354225 \| \| 2.033384672 \| \| 2.487249999 \| \| 2.277315327 \| \| 3.135887074 \| \| 2.607267548 \| \| -3.041078161 \| \| -2.263762408 \| \| -2.058513023 \| \| 2.346693289 \| \| 2.430550084 \| \| 2.118521124 \| \| 2.547225143 \| \| 2.267133491 \| \| 3.499481509 \| \| 2.571119963 \| \| 2.361069104 \| \| 3.387929669 \| \| 2.13443999 \| \| 2.535653378 \| \| 2.021473743 \| \| 2.212884372 \| \| -2.344396712 \| \| 2.126392505 \| \| 2.603886787 \| \| 2.304717831 \| \| 2.660963167 \| \| -2.408178907 \| \| 2.502217126 \| \| 2.095818544 \| \| 2.170655358 \| \| -2.411104464 \| \| 2.84361003 \| \| 2.342380245 \| \| 2.170868268 \| \| 2.074108658 \| \| 3.289956847 \| \| -3.054514478 \| \| 2.003039955 \| \| 2.033622882 \| \| 2.543998661 \| \| -2.004912538 \| \| 2.545235059 \| \| 2.163955447 \| \| 2.377374073 \| \| 2.20786932 \| \| 2.5918055 \| \| 4.094137163 \| \| -2.285324853 \| \| 3.458710855 \| \| 2.379336118 \| \| 2.717560058 \| \| 2.005442916 \| \| 2.15598139 \| \| 2.876221971 \| \| 2.512243693 \| \| 3.311631242 \| \| 3.043275526 \| \| -2.719985459 \| \| -2.221231967 \| \| 2.024703778 \| \| 2.061533019 \| \| 2.079425241 \| \| 2.611268475 \| \| -2.989646901 \| \| 2.321259802 \| \| 2.151871986 \| \| 2.838631472 \| \| 2.380450606 \| \| 3.41853657 \| \| -3.351970554 \| \| -2.808054385 \| \| 2.1687625 \| \| 2.10467027 \| \| 2.212252951 \| \| 2.58224901 \| \| -2.914975861 \| \| 2.141348166 \| \| 2.47591548 \| \| -2.930962005 \| \| 2.592787433 \| \| 3.546595827 \| \| 2.738108759 \| \| 2.981043601 \| \| 2.528075495 \| \| 2.071721587 \| \| -2.074947341 \| \| -2.240941824 \| \| 3.809092423 \| \| 2.102092602 \| \| -3.065773001 \| \| 4.681773477 \| \| 2.408389156 \| \| 2.091128501 \| \| 2.254392289 \| \| 2.096470182 \| \| 2.094271514 \| \| -5.358270514 \| \| 2.834833919 \| \| 2.847829025 \| \| 2.208758448 \| \| 2.164479317 \| \| -2.022515479 \| \| 2.190603441 \| \| 2.354251775 \| \| 2.603140803 \| \| 2.293712867 \| \| -2.542042974 \| \| 2.590987579 \| \| 3.44805671 \| \| 2.942448749 \| \| 3.494464887 \| \| 2.119510014 \| \| -3.159577696 \| \| 2.179379933 \| \| 2.46107711 \| \| 2.920443853 \| \| 2.808768158 \| \| 2.159527943 \| \| 2.414684821 \| \| -2.039421309 \| \| 2.690608796 \| \| 3.231600806 \| \| 3.06747297 \| \| -2.737938279 \| \| 2.044623253 \| \| 3.569494804 \| \| 2.39087269 \| \| 2.49001648 \| \| 3.221151522 \| \| 2.265462393 \| \| 2.662987659 \| \| 2.239615252 \| \| 2.162529298 \| \| 3.220750864 \| \| 2.238710328 \| \| 3.005899622 \| \| 3.033488045 \| \| 2.00642329 \| \| 2.170257879 \| \| -5.074345088 \| \| 2.715306139 \| \| 3.641220412 \| \| 2.480820707 \| \| 3.191068916 \| \| 2.250246065 \| \| 2.271049013 \| \| 2.673439229 \| \| -2.010516054 \| \| 2.059833258 \| \| 2.38317431 \| \| 2.213672141 \| \| 2.109537754 \| \| 2.699933512 \| \| 2.455874542 \| \| -2.13494397 \| \| 2.204735947 \| \| 2.165482924 \| \| 2.569285572 \| \| 2.153162762 \| \| 4.429246303 \| \| 2.437451213 \| \| 2.054948324 \| \| 3.763940272 \| \| 2.011524477 \| \| -2.338015881 \| \| 6.532510196 \| \| 2.105902793 \| \| 2.059213934 \| \| 2.571558906 \| \| 2.383261111 \| \| 2.38072732 \| \| 2.255395706 \| \| 2.421845541 \| \| 2.114258653 \| \| 4.13935891 \| \| 2.698428803 \| \| 2.228165337 \| \| 2.018094097 \| \| 2.07768606 \| \| 2.84304987 \| \| 2.030301353 \| \| 2.272491026 \| \| 2.140972538 \| \| 2.029121187 \| \| 2.244033115 \| \| 2.867764086 \| \| -3.878968115 \| \| 2.020707825 \| \| 2.223318006 \| \| 2.295631661 \| \| 3.702940698 \| \| 2.363360201 \| \| 3.482252233 \| \| 2.002167195 \| \| 2.403890359 \| \| 2.693051497 \| \| 3.591315882 \| \| 2.28902221 \| \| 2.594072499 \| \| -2.150430131 \| \| 2.132442806 \| \| 2.341439592 \| \| 3.163652048 \| \| 2.886362353 \| \| -3.409342573 \| \| 2.363474492 \| \| 2.913368946 \| \| 3.341054617 \| \| -2.311081991 \| \| 2.156890814 \| \| 2.093679021 \| \| -4.376858267 \| \| 3.281288099 \| \| -2.34773793 \| \| 2.434195757 \| \| 2.109417974 \| \| 2.175538012 \| \| 2.490803783 \| \| 2.042114393 \| \| 2.051089814 \| \| 2.293167396 \| \| 3.073383296 \| \| 2.143103135 \| \| 2.266995988 \| \| 2.693949323 \| \| 2.828297759 \| \| 2.079079398 \| \| 2.116962668 \| \| 2.414389204 \| \| 2.667975766 \| \| -2.850567392 \| \| 2.873126363 \| \| 3.548666229 \| \| 2.396642224 \| \| -2.642133945 \| \| 2.086452839 \| \| 3.783318663 \| \| 2.471237397 \| \| 2.12211723 \| \| 3.138333331 \| \| 3.183862929 \| \| 2.478772103 \| \| 4.397588212 \| \| -2.257886969 \| \| 4.109809216 \| \| 2.423899327 \| \| 2.424497046 \| \| -2.496151907 \| \| 2.812429119 \| \| 2.081871092 \| \| 2.552285606 \| \| -2.107771837 \| \| 2.495556502 \| \| 2.818771952 \| \| 2.338732808 \| \| -6.286474684 \| \| 2.151019141 \| \| 2.574911089 \| \| 2.626859752 \| \| 3.141957229 \| \| 2.551730394 \| \| 2.283070894 \| \| 3.681446998 \| \| 2.164677105 \| \| 2.141646949 \| \| 2.060432839 \| \| 3.219482273 \| \| 2.225514655 \| \| 2.386282155 \| \| 2.18269288 \| \| 4.026756575 \| \| 2.339514124 \| \| -2.241904254 \| \| 2.155967965 \| \| 2.503006393 \| \| 2.465252452 \| \| 2.075263594 \| \| 2.12267382 \| \| 2.222642832 \| \| 2.659480864 \| \| -2.264580834 \| \| 2.996173249 \| \| 2.001896917 \| \| 2.423554554 \| \| 3.448365864 \| \| 2.081536426 \| \| 2.100648971 \| \| -2.004169831 \| \| 3.056648354 \| \| 2.129531891 \| \| 2.460711194 \| \| 2.216224659 \| \| -2.263332538 \| \| -2.603096568 \| \| 2.160095097 \| \| 2.180099307 \| \| 2.129571016 \| \| 2.261650481 \| \| -3.29064257 \| \| 3.206832503 \| \| 2.410403454 \| \| 2.370258624 \| \| 3.443696655 \| \| 2.654193766 \| \| 2.875573197 \| \| 3.667783358 \| \| -2.169734228 \| \| 2.162298668 \| \| 2.842433166 \| \| 4.431483849 \| \| 2.482848718 \| \| 3.57725537 \| \| 2.922613346 \| \| 2.726237857 \| \| -4.185624108 \| \| 2.855499108 \| \| 3.414460233 \| \| 2.075051365 \| \| 2.423338386 \| | \| 6.40E-34 \| \| --- \| \| 1.84E-08 \| \| 9.36E-13 \| \| 2.06E-29 \| \| 6.90E-05 \| \| 5.88E-13 \| \| 9.60E-37 \| \| 0.004340003 \| \| 4.41E-05 \| \| 0.000213575 \| \| 9.34E-09 \| \| 3.36E-42 \| \| 3.75E-49 \| \| 9.20E-08 \| \| 4.27E-19 \| \| 1.10E-26 \| \| 5.47E-12 \| \| 1.37E-18 \| \| 1.44E-13 \| \| 9.72E-55 \| \| 3.67E-53 \| \| 1.18E-12 \| \| 1.84E-41 \| \| 1.05E-45 \| \| 1.38E-30 \| \| 6.00E-36 \| \| 0.005893101 \| \| 2.31E-26 \| \| 4.72E-11 \| \| 2.42E-36 \| \| 2.42E-08 \| \| 4.63E-38 \| \| 2.29E-22 \| \| 1.75E-43 \| \| 1.16E-43 \| \| 2.73E-48 \| \| 4.36E-13 \| \| 2.15E-36 \| \| 6.66E-45 \| \| 1.44E-08 \| \| 1.27E-21 \| \| 9.38E-40 \| \| 4.19E-14 \| \| 2.98E-38 \| \| 1.08E-29 \| \| 3.84E-05 \| \| 1.73E-41 \| \| 5.54E-31 \| \| 2.83E-40 \| \| 5.04E-22 \| \| 1.53E-25 \| \| 5.11E-49 \| \| 6.94E-29 \| \| 0.000226627 \| \| 2.92E-13 \| \| 2.90E-30 \| \| 6.61E-05 \| \| 5.80E-15 \| \| 6.12E-39 \| \| 5.73E-23 \| \| 8.43E-18 \| \| 2.18E-39 \| \| 2.82E-38 \| \| 2.83E-10 \| \| 1.48E-34 \| \| 4.37E-41 \| \| 5.22E-37 \| \| 1.29E-20 \| \| 4.56E-09 \| \| 0.017507893 \| \| 6.61E-33 \| \| 3.28E-16 \| \| 8.57E-11 \| \| 1.54E-08 \| \| 4.43E-25 \| \| 1.24E-23 \| \| 3.46E-20 \| \| 1.77E-05 \| \| 4.28E-21 \| \| 2.77E-22 \| \| 5.55E-26 \| \| 6.01E-06 \| \| 1.54E-16 \| \| 8.74E-42 \| \| 5.46E-13 \| \| 3.58E-28 \| \| 2.62E-23 \| \| 1.20E-10 \| \| 1.13E-23 \| \| 0.018521346 \| \| 7.89E-34 \| \| 1.05E-17 \| \| 4.19E-40 \| \| 1.85E-37 \| \| 3.88E-18 \| \| 8.55E-09 \| \| 4.95E-11 \| \| 1.18E-37 \| \| 0.001075017 \| \| 5.19E-37 \| \| 2.02E-29 \| \| 6.10E-52 \| \| 2.57E-44 \| \| 7.55E-46 \| \| 4.57E-19 \| \| 0.000144519 \| \| 9.04E-06 \| \| 1.21E-44 \| \| 1.09E-38 \| \| 6.66E-08 \| \| 0.001376343 \| \| 3.94E-47 \| \| 2.38E-17 \| \| 2.94E-37 \| \| 2.29E-40 \| \| 1.51E-26 \| \| 0.005125581 \| \| 2.66E-32 \| \| 3.99E-30 \| \| 6.64E-28 \| \| 6.27E-40 \| \| 2.60E-48 \| \| 1.39E-06 \| \| 3.47E-09 \| \| 1.56E-38 \| \| 1.75E-49 \| \| 1.79E-45 \| \| 2.93E-10 \| \| 1.85E-49 \| \| 1.91E-32 \| \| 2.79E-09 \| \| 1.13E-30 \| \| 7.21E-07 \| \| 0.001701025 \| \| 3.66E-12 \| \| 5.65E-43 \| \| 2.08E-47 \| \| 2.47E-41 \| \| 4.41E-09 \| \| 7.60E-17 \| \| 5.38E-29 \| \| 3.30E-49 \| \| 2.23E-39 \| \| 6.88E-38 \| \| 1.71E-16 \| \| 2.31E-21 \| \| 2.33E-11 \| \| 2.17E-46 \| \| 1.02E-14 \| \| 1.71E-24 \| \| 4.22E-06 \| \| 3.16E-33 \| \| 3.03E-08 \| \| 0.021139629 \| \| 1.03E-35 \| \| 2.02E-34 \| \| 4.84E-14 \| \| 3.73E-07 \| \| 1.86E-10 \| \| 6.91E-29 \| \| 1.83E-22 \| \| 2.94E-20 \| \| 4.22E-22 \| \| 2.97E-20 \| \| 8.03E-46 \| \| 7.07E-34 \| \| 2.10E-17 \| \| 2.20E-05 \| \| 9.27E-36 \| \| 0.001954552 \| \| 8.95E-29 \| \| 2.97E-38 \| \| 3.24E-12 \| \| 2.27E-27 \| \| 1.95E-13 \| \| 4.56E-19 \| \| 3.96E-27 \| \| 2.78E-43 \| \| 6.36E-16 \| \| 1.04E-15 \| \| 1.21E-45 \| \| 2.66E-17 \| \| 1.56E-20 \| \| 5.63E-44 \| \| 6.07E-06 \| \| 1.29E-18 \| \| 2.34E-42 \| \| 1.25E-29 \| \| 5.82E-37 \| \| 5.14E-18 \| \| 5.82E-16 \| \| 4.02E-12 \| \| 4.32E-33 \| \| 1.56E-14 \| \| 3.68E-43 \| \| 1.76E-29 \| \| 8.99E-08 \| \| 1.26E-10 \| \| 5.05E-46 \| \| 2.16E-41 \| \| 5.32E-10 \| \| 3.87E-28 \| \| 3.18E-07 \| \| 7.05E-32 \| \| 1.44E-27 \| \| 1.75E-13 \| \| 7.54E-41 \| \| 1.01E-44 \| \| 2.77E-27 \| \| 6.27E-18 \| \| 9.67E-22 \| \| 3.48E-37 \| \| 2.91E-06 \| \| 1.46E-10 \| \| 0.0002505 \| \| 0.021034763 \| \| 0.031824952 \| \| 0.031002249 \| \| 1.14E-39 \| \| 5.79E-41 \| \| 3.91E-23 \| \| 1.46E-45 \| \| 1.54E-18 \| \| 0.003113761 \| \| 1.71E-11 \| \| 4.36E-08 \| \| 3.03E-19 \| \| 2.03E-28 \| \| 0.005070124 \| \| 4.13E-30 \| \| 2.09E-19 \| \| 1.26E-48 \| \| 0.001268775 \| \| 1.11E-08 \| \| 4.90E-47 \| \| 1.02E-35 \| \| 1.34E-46 \| \| 2.38E-39 \| \| 2.32E-51 \| \| 0.000142342 \| \| 2.17E-42 \| \| 7.30E-14 \| \| 1.50E-43 \| \| 1.90E-23 \| \| 1.54E-14 \| \| 9.36E-38 \| \| 3.68E-16 \| \| 2.95E-46 \| \| 1.71E-27 \| \| 9.46E-26 \| \| 0.029296331 \| \| 7.22E-21 \| \| 3.60E-27 \| \| 8.13E-10 \| \| 7.32E-49 \| \| 7.07E-27 \| \| 7.02E-33 \| \| 5.20E-45 \| \| 3.94E-41 \| \| 7.37E-23 \| \| 4.64E-41 \| \| 6.27E-47 \| \| 2.16E-48 \| \| 8.27E-05 \| \| 2.98E-39 \| \| 1.34E-24 \| \| 3.01E-09 \| \| 4.96E-34 \| \| 5.10E-10 \| \| 9.54E-40 \| \| 2.10E-28 \| \| 1.12E-45 \| \| 4.35E-38 \| \| 8.22E-35 \| \| 0.015082101 \| \| 2.43E-42 \| \| 1.85E-10 \| \| 1.85E-16 \| \| 3.08E-22 \| \| 4.64E-40 \| \| 1.24E-38 \| \| 7.70E-12 \| \| 1.45E-55 \| \| 5.87E-24 \| \| 2.52E-37 \| \| 1.08E-19 \| \| 1.06E-33 \| \| 2.99E-42 \| \| 1.42E-41 \| \| 5.37E-15 \| \| 2.35E-48 \| \| 5.18E-18 \| \| 6.98E-12 \| \| 1.08E-34 \| \| 9.29E-24 \| \| 1.46E-40 \| \| 2.05E-45 \| \| 1.95E-41 \| \| 5.24E-24 \| \| 3.19E-44 \| \| 1.82E-36 \| \| 6.02E-10 \| \| 5.74E-38 \| \| 6.55E-20 \| \| 4.99E-16 \| \| 3.33E-42 \| \| 2.89E-52 \| \| 1.95E-09 \| \| 5.45E-50 \| \| 4.52E-37 \| \| 4.19E-31 \| \| 7.02E-46 \| \| 1.12E-45 \| \| 9.18E-11 \| \| 1.54E-05 \| \| 1.46E-16 \| \| 6.79E-17 \| \| 3.33E-13 \| \| 1.53E-26 \| \| 1.67E-43 \| \| 5.53E-31 \| \| 2.52E-39 \| \| 1.13E-08 \| \| 2.04E-36 \| \| 2.74E-47 \| \| 1.01E-46 \| \| 1.99E-18 \| \| 1.19E-31 \| \| 1.14E-11 \| \| 6.08E-11 \| \| 3.05E-16 \| \| 3.04E-06 \| \| 6.92E-20 \| \| 1.43E-25 \| \| 1.32E-37 \| \| 0.000321755 \| \| 9.42E-09 \| \| 6.09E-32 \| \| 5.43E-09 \| \| 9.56E-16 \| \| 3.02E-08 \| \| 2.89E-08 \| \| 2.87E-43 \| \| 6.36E-27 \| \| 1.46E-13 \| \| 1.22E-11 \| \| 2.39E-21 \| \| 8.86E-31 \| \| 3.41E-37 \| \| 1.99E-05 \| \| 6.21E-12 \| \| 9.92E-34 \| \| 1.05E-29 \| \| 6.81E-53 \| \| 4.87E-41 \| \| 1.47E-41 \| \| 1.20E-07 \| \| 2.49E-18 \| \| 1.49E-23 \| \| 3.23E-30 \| \| 9.77E-49 \| \| 1.93E-08 \| \| 5.93E-43 \| \| 2.53E-11 \| \| 4.49E-38 \| \| 2.94E-27 \| \| 2.58E-17 \| \| 9.15E-09 \| \| 4.63E-05 \| \| 0.000129795 \| \| 2.59E-20 \| \| 3.36E-09 \| \| 5.24E-50 \| \| 7.91E-20 \| \| 1.21E-15 \| \| 8.20E-51 \| \| 6.58E-14 \| \| 2.34E-44 \| \| 1.38E-09 \| \| 4.97E-36 \| \| 1.83E-24 \| \| 4.30E-10 \| \| 0.015643796 \| \| 1.17E-06 \| \| 8.72E-38 \| \| 1.40E-06 \| \| 1.40E-23 \| \| 1.33E-46 \| \| 0.004476356 \| \| 0.008313531 \| \| 3.28E-34 \| \| 2.36E-06 \| \| 2.13E-39 \| \| 1.41E-26 \| \| 2.09E-24 \| \| 2.43E-33 \| \| 1.99E-29 \| \| 5.26E-29 \| \| 2.35E-32 \| \| 6.34E-10 \| \| 0.005773977 \| \| 2.84E-36 \| \| 5.30E-30 \| \| 8.71E-37 \| \| 3.88E-23 \| \| 8.07E-25 \| \| 7.78E-40 \| \| 1.75E-05 \| \| 4.89E-49 \| \| 1.51E-28 \| \| 4.23E-45 \| \| 4.12E-33 \| \| 4.01E-42 \| \| 7.46E-11 \| \| 2.86E-14 \| \| 6.28E-29 \| \| 1.75E-38 \| \| 3.83E-11 \| \| 1.43E-39 \| \| 8.59E-13 \| \| 1.50E-19 \| \| 1.95E-43 \| \| 4.84E-13 \| \| 2.40E-43 \| \| 3.17E-43 \| \| 6.23E-07 \| \| 1.11E-49 \| \| 1.05E-48 \| \| 4.70E-33 \| \| 8.26E-23 \| \| 2.60E-49 \| \| 4.00E-12 \| \| 3.82E-34 \| \| 1.72E-15 \| \| 9.05E-45 \| \| 5.10E-28 \| \| 9.68E-13 \| \| 2.15E-41 \| \| 4.94E-05 \| \| 2.05E-48 \| \| 1.37E-20 \| \| 5.34E-06 \| \| 6.40E-48 \| \| 3.79E-35 \| \| 4.67E-23 \| \| 2.03E-27 \| | \| 2.95E-32 \| \| --- \| \| 5.70E-08 \| \| 4.78E-12 \| \| 5.78E-28 \| \| 0.000136764 \| \| 3.07E-12 \| \| 6.06E-35 \| \| 0.006657736 \| \| 8.95E-05 \| \| 0.000394677 \| \| 3.00E-08 \| \| 5.25E-40 \| \| 3.13E-46 \| \| 2.61E-07 \| \| 4.53E-18 \| \| 2.38E-25 \| \| 2.55E-11 \| \| 1.38E-17 \| \| 8.13E-13 \| \| 6.90E-51 \| \| 1.74E-49 \| \| 5.94E-12 \| \| 2.46E-39 \| \| 3.24E-43 \| \| 4.37E-29 \| \| 3.55E-34 \| \| 0.008853684 \| \| 4.92E-25 \| \| 1.96E-10 \| \| 1.46E-34 \| \| 7.39E-08 \| \| 3.41E-36 \| \| 3.39E-21 \| \| 3.56E-41 \| \| 2.49E-41 \| \| 1.39E-45 \| \| 2.32E-12 \| \| 1.31E-34 \| \| 1.66E-42 \| \| 4.51E-08 \| \| 1.73E-20 \| \| 9.12E-38 \| \| 2.52E-13 \| \| 2.24E-36 \| \| 3.16E-28 \| \| 7.86E-05 \| \| 2.34E-39 \| \| 1.82E-29 \| \| 3.02E-38 \| \| 7.17E-21 \| \| 2.99E-24 \| \| 3.82E-46 \| \| 1.83E-27 \| \| 0.000417005 \| \| 1.59E-12 \| \| 8.91E-29 \| \| 0.000131415 \| \| 3.85E-14 \| \| 5.02E-37 \| \| 8.93E-22 \| \| 7.69E-17 \| \| 1.93E-37 \| \| 2.16E-36 \| \| 1.07E-09 \| \| 7.39E-33 \| \| 5.26E-39 \| \| 3.38E-35 \| \| 1.60E-19 \| \| 1.52E-08 \| \| 0.024542514 \| \| 2.69E-31 \| \| 2.51E-15 \| \| 3.46E-10 \| \| 4.82E-08 \| \| 8.48E-24 \| \| 2.05E-22 \| \| 4.06E-19 \| \| 3.79E-05 \| \| 5.54E-20 \| \| 4.08E-21 \| \| 1.14E-24 \| \| 1.37E-05 \| \| 1.22E-15 \| \| 1.24E-39 \| \| 2.86E-12 \| \| 8.92E-27 \| \| 4.24E-22 \| \| 4.74E-10 \| \| 1.87E-22 \| \| 0.025881388 \| \| 3.59E-32 \| \| 9.46E-17 \| \| 4.37E-38 \| \| 1.27E-35 \| \| 3.68E-17 \| \| 2.76E-08 \| \| 2.06E-10 \| \| 8.31E-36 \| \| 0.001811817 \| \| 3.38E-35 \| \| 5.69E-28 \| \| 1.44E-48 \| \| 5.89E-42 \| \| 2.44E-43 \| \| 4.83E-18 \| \| 0.000273363 \| \| 2.02E-05 \| \| 2.86E-42 \| \| 8.78E-37 \| \| 1.93E-07 \| \| 0.002283077 \| \| 1.70E-44 \| \| 2.07E-16 \| \| 1.99E-35 \| \| 2.48E-38 \| \| 3.24E-25 \| \| 0.007771307 \| \| 9.95E-31 \| \| 1.20E-28 \| \| 1.61E-26 \| \| 6.31E-38 \| \| 1.37E-45 \| \| 3.44E-06 \| \| 1.17E-08 \| \| 1.22E-36 \| \| 1.87E-46 \| \| 4.89E-43 \| \| 1.11E-09 \| \| 1.87E-46 \| \| 7.30E-31 \| \| 9.52E-09 \| \| 3.60E-29 \| \| 1.84E-06 \| \| 0.002781901 \| \| 1.75E-11 \| \| 9.79E-41 \| \| 9.55E-45 \| \| 3.10E-39 \| \| 1.47E-08 \| \| 6.25E-16 \| \| 1.46E-27 \| \| 2.93E-46 \| \| 1.96E-37 \| \| 4.98E-36 \| \| 1.36E-15 \| \| 3.09E-20 \| \| 1.01E-10 \| \| 7.90E-44 \| \| 6.60E-14 \| \| 3.08E-23 \| \| 9.80E-06 \| \| 1.34E-31 \| \| 9.14E-08 \| \| 0.029272159 \| \| 5.76E-34 \| \| 9.94E-33 \| \| 2.89E-13 \| \| 9.91E-07 \| \| 7.20E-10 \| \| 1.83E-27 \| \| 2.73E-21 \| \| 3.49E-19 \| \| 6.05E-21 \| \| 3.52E-19 \| \| 2.53E-43 \| \| 3.24E-32 \| \| 1.83E-16 \| \| 4.65E-05 \| \| 5.30E-34 \| \| 0.003167446 \| \| 2.34E-27 \| \| 2.24E-36 \| \| 1.55E-11 \| \| 5.22E-26 \| \| 1.08E-12 \| \| 4.82E-18 \| \| 8.96E-26 \| \| 5.34E-41 \| \| 4.73E-15 \| \| 7.53E-15 \| \| 3.52E-43 \| \| 2.30E-16 \| \| 1.90E-19 \| \| 1.25E-41 \| \| 1.38E-05 \| \| 1.30E-17 \| \| 3.87E-40 \| \| 3.60E-28 \| \| 3.75E-35 \| \| 4.82E-17 \| \| 4.34E-15 \| \| 1.91E-11 \| \| 1.81E-31 \| \| 9.93E-14 \| \| 6.69E-41 \| \| 5.00E-28 \| \| 2.55E-07 \| \| 4.97E-10 \| \| 1.71E-43 \| \| 2.76E-39 \| \| 1.97E-09 \| \| 9.60E-27 \| \| 8.53E-07 \| \| 2.50E-30 \| \| 3.38E-26 \| \| 9.79E-13 \| \| 8.64E-39 \| \| 2.43E-42 \| \| 6.35E-26 \| \| 5.78E-17 \| \| 1.33E-20 \| \| 2.32E-35 \| \| 6.90E-06 \| \| 5.70E-10 \| \| 0.000458792 \| \| 0.029135476 \| \| 0.042859977 \| \| 0.041823427 \| \| 1.09E-37 \| \| 6.74E-39 \| \| 6.18E-22 \| \| 4.14E-43 \| \| 1.54E-17 \| \| 0.004883224 \| \| 7.49E-11 \| \| 1.29E-07 \| \| 3.26E-18 \| \| 5.15E-27 \| \| 0.007691332 \| \| 1.23E-28 \| \| 2.27E-18 \| \| 7.76E-46 \| \| 0.002117509 \| \| 3.54E-08 \| \| 2.04E-44 \| \| 5.75E-34 \| \| 5.02E-44 \| \| 2.07E-37 \| \| 4.70E-48 \| \| 0.000269532 \| \| 3.63E-40 \| \| 4.28E-13 \| \| 3.14E-41 \| \| 3.10E-22 \| \| 9.79E-14 \| \| 6.68E-36 \| \| 2.80E-15 \| \| 1.05E-43 \| \| 3.99E-26 \| \| 1.90E-24 \| \| 0.039672871 \| \| 9.17E-20 \| \| 8.20E-26 \| \| 2.94E-09 \| \| 5.19E-46 \| \| 1.56E-25 \| \| 2.86E-31 \| \| 1.32E-42 \| \| 4.86E-39 \| \| 1.14E-21 \| \| 5.54E-39 \| \| 2.54E-44 \| \| 1.23E-45 \| \| 0.000161899 \| \| 2.54E-37 \| \| 2.44E-23 \| \| 1.02E-08 \| \| 2.30E-32 \| \| 1.89E-09 \| \| 9.21E-38 \| \| 5.30E-27 \| \| 3.32E-43 \| \| 3.23E-36 \| \| 4.20E-33 \| \| 0.021331624 \| \| 3.96E-40 \| \| 7.17E-10 \| \| 1.46E-15 \| \| 4.52E-21 \| \| 4.77E-38 \| \| 9.87E-37 \| \| 3.52E-11 \| \| 2.06E-51 \| \| 1.01E-22 \| \| 1.72E-35 \| \| 1.20E-18 \| \| 4.67E-32 \| \| 4.78E-40 \| \| 1.96E-39 \| \| 3.59E-14 \| \| 1.28E-45 \| \| 4.85E-17 \| \| 3.21E-11 \| \| 5.46E-33 \| \| 1.55E-22 \| \| 1.64E-38 \| \| 5.45E-43 \| \| 2.54E-39 \| \| 9.09E-23 \| \| 7.19E-42 \| \| 1.12E-34 \| \| 2.21E-09 \| \| 4.18E-36 \| \| 7.45E-19 \| \| 3.74E-15 \| \| 5.25E-40 \| \| 8.20E-49 \| \| 6.76E-09 \| \| 7.03E-47 \| \| 3.00E-35 \| \| 1.39E-29 \| \| 2.32E-43 \| \| 3.32E-43 \| \| 3.69E-10 \| \| 3.33E-05 \| \| 1.16E-15 \| \| 5.62E-16 \| \| 1.79E-12 \| \| 3.28E-25 \| \| 3.44E-41 \| \| 1.82E-29 \| \| 2.17E-37 \| \| 3.61E-08 \| \| 1.25E-34 \| \| 1.21E-44 \| \| 3.97E-44 \| \| 1.95E-17 \| \| 4.12E-30 \| \| 5.09E-11 \| \| 2.49E-10 \| \| 2.34E-15 \| \| 7.18E-06 \| \| 7.84E-19 \| \| 2.83E-24 \| \| 9.18E-36 \| \| 0.00058159 \| \| 3.02E-08 \| \| 2.18E-30 \| \| 1.79E-08 \| \| 6.98E-15 \| \| 9.11E-08 \| \| 8.74E-08 \| \| 5.43E-41 \| \| 1.41E-25 \| \| 8.24E-13 \| \| 5.42E-11 \| \| 3.18E-20 \| \| 2.85E-29 \| \| 2.28E-35 \| \| 4.23E-05 \| \| 2.88E-11 \| \| 4.41E-32 \| \| 3.07E-28 \| \| 2.42E-49 \| \| 5.77E-39 \| \| 2.00E-39 \| \| 3.38E-07 \| \| 2.41E-17 \| \| 2.45E-22 \| \| 9.83E-29 \| \| 6.61E-46 \| \| 5.96E-08 \| \| 1.01E-40 \| \| 1.09E-10 \| \| 3.32E-36 \| \| 6.72E-26 \| \| 2.23E-16 \| \| 2.94E-08 \| \| 9.38E-05 \| \| 0.000246961 \| \| 3.08E-19 \| \| 1.14E-08 \| \| 7.03E-47 \| \| 8.90E-19 \| \| 8.74E-15 \| \| 1.45E-47 \| \| 3.88E-13 \| \| 5.44E-42 \| \| 4.86E-09 \| \| 2.95E-34 \| \| 3.28E-23 \| \| 1.60E-09 \| \| 0.022062321 \| \| 2.92E-06 \| \| 6.29E-36 \| \| 3.47E-06 \| \| 2.31E-22 \| \| 5.02E-44 \| \| 0.006857273 \| \| 0.012210956 \| \| 1.56E-32 \| \| 5.66E-06 \| \| 1.91E-37 \| \| 3.04E-25 \| \| 3.73E-23 \| \| 1.04E-31 \| \| 5.62E-28 \| \| 1.43E-27 \| \| 8.84E-31 \| \| 2.32E-09 \| \| 0.008684825 \| \| 1.70E-34 \| \| 1.57E-28 \| \| 5.54E-35 \| \| 6.15E-22 \| \| 1.50E-23 \| \| 7.62E-38 \| \| 3.76E-05 \| \| 3.82E-46 \| \| 3.88E-27 \| \| 1.09E-42 \| \| 1.72E-31 \| \| 6.05E-40 \| \| 3.03E-10 \| \| 1.76E-13 \| \| 1.67E-27 \| \| 1.35E-36 \| \| 1.61E-10 \| \| 1.34E-37 \| \| 4.42E-12 \| \| 1.65E-18 \| \| 3.90E-41 \| \| 2.56E-12 \| \| 4.73E-41 \| \| 5.85E-41 \| \| 1.61E-06 \| \| 1.31E-46 \| \| 6.80E-46 \| \| 1.95E-31 \| \| 1.27E-21 \| \| 2.46E-46 \| \| 1.90E-11 \| \| 1.80E-32 \| \| 1.22E-14 \| \| 2.22E-42 \| \| 1.25E-26 \| \| 4.93E-12 \| \| 2.76E-39 \| \| 9.97E-05 \| \| 1.21E-45 \| \| 1.68E-19 \| \| 1.22E-05 \| \| 3.13E-45 \| \| 2.00E-33 \| \| 7.34E-22 \| \| 4.69E-26 \| |
