# Supplementary material for: Identification of anoikis-related molecular patterns and the novel risk model to predict prognosis, tumor microenvironment infiltration and immunotherapy response in bladder cancer
Source: Front Immunol. 2024 Nov 27;15:1491808. doi: 10.3389/fimmu.2024.1491808 (PMC11631915; doi:10.3389/fimmu.2024.1491808)
Supplement: Supplementary file 13 [file Table6.docx]

**Table S6. GO and KEGG results of anoikis phenotype-associated differently expressed genes (DEGs).**

| Oncology | ID | Description | P value | P adjust |
| --- | --- | --- | --- | --- |
| \| BP \| \| --- \| \| BP \| \| BP \| \| BP \| \| BP  CC  CC  CC  CC  CC  MF  MF  MF  MF  MF  KEGG  KEGG  KEGG  KEGG  KEGG \| | \| \| GO:0030198 \| \| --- \| \| GO:0043062 \| \| GO:0045229 \| \| GO:0030199 \| \| GO:0001503 \| \| GO:0062023 \| \| GO:0005581 \| \| GO:0005583 \| \| GO:0098643 \| \| GO:0098644 \| \| GO:0005201 \| \| GO:0005539 \| \| GO:0008201 \| \| GO:0005518 \| \| GO:1901681  hsa04512  hsa04974  hsa04061  hsa04510  hsa04060 \| \| \| --- \| --- \| --- \| --- \| --- \| --- \| --- \| --- \| --- \| --- \| --- \| --- \| --- \| --- \| --- \| --- \| | \| extracellular matrix organization \| \| --- \| \| extracellular structure organization \| \| external encapsulating structure organization \| \| collagen fibril organization \| \| ossification \| \| collagen-containing extracellular matrix \| \| collagen trimer \| \| fibrillar collagen trimer \| \| banded collagen fibril \| \| complex of collagen trimers \| \| extracellular matrix structural constituent \| \| glycosaminoglycan binding \| \| heparin binding \| \| collagen binding \| \| sulfur compound binding  ECM-receptor interaction  Protein digestion and absorption  Viral protein interaction with cytokine and cytokine receptor  Focal adhesion  Cytokine-cytokine receptor interaction \| | \| \| 3.21891E-36 \| \| --- \| \| 3.8751E-36 \| \| 5.6037E-36 \| \| 1.13548E-22 \| \| 2.0398E-19 \| \| 1.67935E-45 \| \| 1.69429E-16 \| \| 2.45225E-13 \| \| 2.45225E-13 \| \| 1.3075E-11 \| \| 1.27911E-31 \| \| 6.32189E-21 \| \| 1.0368E-17 \| \| 6.81486E-15 \| \| 1.10073E-14  9.48065E-11  1.25082E-09  6.24165E-09  1.33713E-08  2.22929E-07 \| \| \| --- \| --- \| --- \| --- \| --- \| --- \| --- \| --- \| --- \| --- \| --- \| --- \| --- \| --- \| --- \| --- \| | \| 6.94298E-33 \| \| --- \| \| 6.94298E-33 \| \| 6.94298E-33 \| \| 1.05515E-19 \| \| 1.51639E-16 \| \| 5.62583E-43 \| \| 2.83794E-14 \| \| 2.05376E-11 \| \| 2.05376E-11 \| \| 8.5358E-10 \| \| 6.61301E-29 \| \| 1.63421E-18 \| \| 1.78676E-15 \| \| 8.8082E-13 \| \| 1.13815E-12  2.26588E-08  1.49473E-07  4.97252E-07  7.98935E-07  1.0656E-05 \| |
