# Supplementary material for: Identification of anoikis-related molecular patterns and the novel risk model to predict prognosis, tumor microenvironment infiltration and immunotherapy response in bladder cancer
Source: Front Immunol. 2024 Nov 27;15:1491808. doi: 10.3389/fimmu.2024.1491808 (PMC11631915; doi:10.3389/fimmu.2024.1491808)
Supplement: Supplementary file 14 [file Table7.docx]

**Table S7. The results of 24 genes performed by the univariate Cox analysis.**

| Gene | HR | HR.95L | HR.95H | P value |
| --- | --- | --- | --- | --- |
| \| NEFL \| \| --- \| \| MT1A \| \| GXYLT2 \| \| MRO \| \| NAV3 \| \| IGF1 \| \| IL37 \| \| GFPT2 \| \| LOX \| \| FN1 \| \| KCNE4 \| \| INSYN2B \| \| SPINK14 \| \| LYVE1 \| \| FAM180A \| \| PDGFRB \| \| COL5A3 \| \| KANK4 \| \| SRPX \| \| PPY \| \| FBN1 \| \| ADAMTS16 \| \| COL5A2 \| \| IGFL2 \| | \| 1.014306002 \| \| --- \| \| 1.013054678 \| \| 1.115701985 \| \| 1.562232525 \| \| 1.557702599 \| \| 1.348262282 \| \| 1.028693829 \| \| 1.025658399 \| \| 1.036592895 \| \| 1.001241184 \| \| 1.085911998 \| \| 1.351670927 \| \| 0.054285158 \| \| 1.019330673 \| \| 1.263326841 \| \| 1.015250817 \| \| 1.036370514 \| \| 1.087230653 \| \| 1.013954472 \| \| 1.015557942 \| \| 1.038631883 \| \| 1.135481023 \| \| 1.004757877 \| \| 1.010880351 \| | \| 1.008118063 \| \| --- \| \| 1.006149116 \| \| 1.049483431 \| \| 1.245366161 \| \| 1.219812541 \| \| 1.186050866 \| \| 1.015225069 \| \| 1.012644881 \| \| 1.01603357 \| \| 1.000699441 \| \| 1.038643014 \| \| 1.131523125 \| \| 0.009630677 \| \| 1.007867102 \| \| 1.148392055 \| \| 1.006816281 \| \| 1.01782085 \| \| 1.04502043 \| \| 1.007610715 \| \| 1.00697839 \| \| 1.016975462 \| \| 1.06477962 \| \| 1.001952093 \| \| 1.00535795 \| | \| 1.020531924 \| \| --- \| \| 1.020007636 \| \| 1.186098686 \| \| 1.959721195 \| \| 1.989188752 \| \| 1.532658702 \| \| 1.042341276 \| \| 1.038839154 \| \| 1.057568235 \| \| 1.00178322 \| \| 1.135332209 \| \| 1.614650425 \| \| 0.305988694 \| \| 1.030924631 \| \| 1.389764672 \| \| 1.023756013 \| \| 1.055258243 \| \| 1.131145821 \| \| 1.020338168 \| \| 1.024210594 \| \| 1.060749476 \| \| 1.210877001 \| \| 1.007571517 \| \| 1.016433086 \| | \| 5.37E-06 \| \| --- \| \| 0.000201933 \| \| 0.000453023 \| \| 0.0001147 \| \| 0.000381348 \| \| 4.90E-06 \| \| 2.59E-05 \| \| 0.000100788 \| \| 0.000437766 \| \| 7.05E-06 \| \| 0.000283742 \| \| 0.000892961 \| \| 0.000959537 \| \| 0.000906742 \| \| 1.56E-06 \| \| 0.000376656 \| \| 0.000105814 \| \| 3.48E-05 \| \| 1.51E-05 \| \| 0.000361776 \| \| 0.000422376 \| \| 0.000107254 \| \| 0.000878406 \| \| 0.000107999 \| |
