# Supplementary material for: Identification of anoikis-related molecular patterns and the novel risk model to predict prognosis, tumor microenvironment infiltration and immunotherapy response in bladder cancer
Source: Front Immunol. 2024 Nov 27;15:1491808. doi: 10.3389/fimmu.2024.1491808 (PMC11631915; doi:10.3389/fimmu.2024.1491808)
Supplement: Supplementary file 16 [file Table9.docx]

**Table S9. 86 differentially expressed genes (DEGs) were identified among three gene subtypes.**

FN1

CALU

FAP

MMP14

ADAMTS12

LOXL2

COL18A1

CHST11

SYNDIG1

INHBA

TNC

ARL4C

CAVIN3

RASSF8

UBTD1

ADAMTS6

SPOCK1

ADAM19

PDGFB

PMEPA1

BMP8A

PPFIBP1

CYTH3

PLOD1

RFX8

ARHGAP22

TGFBI

LAMB1

SERPINE1

NCOR2

EVA1A

MATN3

MAP4K4

SULF2

FSTL3

P4HA2

CSPG4

GNA12

PLIN3

HS3ST3A1

DCBLD2

KANK4

CD109

WWC3

DEGS1

SLC2A3

NT5E

LIMK1

LAYN

FRMD5

ANXA2

MTHFD1L

RNF217

PCDHGC3

PSAP

DCBLD1

GOLT1B

CAPN2

C8orf58

ESYT2

RASGRF1

FLRT2

SNAI2

RECQL

ADCY7

SLC17A9

ATP10D

CDH13

ITPRID2

ARNTL

PITPNM1

SUSD5

BPI

NOG

CDCP1

ARHGAP29

GJA5

MYO10

RAPGEF1

AMZ1

HACD4

APCDD1L

ERRFI1

RIC8A

EHBP1

OSR2
