# Supplementary material for: Identification of anoikis-related molecular patterns and the novel risk model to predict prognosis, tumor microenvironment infiltration and immunotherapy response in bladder cancer
Source: Front Immunol. 2024 Nov 27;15:1491808. doi: 10.3389/fimmu.2024.1491808 (PMC11631915; doi:10.3389/fimmu.2024.1491808)
Supplement: Supplementary file 17 [file Table10.docx]

**Table S10. BLCA patients in the training set.**

| Sample | Survival time | Survival status |
| --- | --- | --- |
| \| TCGA-2F-A9KQ \| \| --- \| \| TCGA-2F-A9KT \| \| TCGA-2F-A9KW \| \| TCGA-4Z-AA7M \| \| TCGA-4Z-AA7O \| \| TCGA-4Z-AA7R \| \| TCGA-4Z-AA7S \| \| TCGA-4Z-AA7Y \| \| TCGA-4Z-AA80 \| \| TCGA-4Z-AA81 \| \| TCGA-4Z-AA83 \| \| TCGA-4Z-AA89 \| \| TCGA-BL-A0C8 \| \| TCGA-BL-A13I \| \| TCGA-BL-A13J \| \| TCGA-BL-A3JM \| \| TCGA-BL-A5ZZ \| \| TCGA-BT-A0S7 \| \| TCGA-BT-A20N \| \| TCGA-BT-A20Q \| \| TCGA-BT-A20T \| \| TCGA-BT-A20U \| \| TCGA-BT-A20V \| \| TCGA-BT-A20X \| \| TCGA-BT-A2LB \| \| TCGA-BT-A3PJ \| \| TCGA-BT-A42C \| \| TCGA-BT-A42E \| \| TCGA-BT-A42F \| \| TCGA-C4-A0EZ \| \| TCGA-C4-A0F0 \| \| TCGA-C4-A0F6 \| \| TCGA-CF-A27C \| \| TCGA-CF-A3MF \| \| TCGA-CF-A3MG \| \| TCGA-CF-A3MH \| \| TCGA-CF-A47V \| \| TCGA-CF-A47W \| \| TCGA-CF-A47Y \| \| TCGA-CF-A8HX \| \| TCGA-CF-A8HY \| \| TCGA-CF-A9FL \| \| TCGA-CF-A9FM \| \| TCGA-CU-A0YO \| \| TCGA-CU-A0YR \| \| TCGA-CU-A3YL \| \| TCGA-CU-A5W6 \| \| TCGA-DK-A1A3 \| \| TCGA-DK-A1A6 \| \| TCGA-DK-A1AA \| \| TCGA-DK-A1AD \| \| TCGA-DK-A1AE \| \| TCGA-DK-A1AF \| \| TCGA-DK-A2HX \| \| TCGA-DK-A2I1 \| \| TCGA-DK-A2I4 \| \| TCGA-DK-A3IL \| \| TCGA-DK-A3IN \| \| TCGA-DK-A3IQ \| \| TCGA-DK-A3IV \| \| TCGA-DK-A3WW \| \| TCGA-DK-A3WX \| \| TCGA-DK-A3X2 \| \| TCGA-DK-A6B1 \| \| TCGA-DK-A6B6 \| \| TCGA-DK-AA6L \| \| TCGA-DK-AA6P \| \| TCGA-DK-AA6Q \| \| TCGA-DK-AA6R \| \| TCGA-DK-AA71 \| \| TCGA-E5-A2PC \| \| TCGA-E5-A4U1 \| \| TCGA-E7-A519 \| \| TCGA-E7-A5KF \| \| TCGA-E7-A677 \| \| TCGA-E7-A6MD \| \| TCGA-E7-A7PW \| \| TCGA-E7-A7XN \| \| TCGA-FD-A3B5 \| \| TCGA-FD-A3B7 \| \| TCGA-FD-A3SJ \| \| TCGA-FD-A3SM \| \| TCGA-FD-A3SO \| \| TCGA-FD-A3SP \| \| TCGA-FD-A3SR \| \| TCGA-FD-A3SS \| \| TCGA-FD-A43U \| \| TCGA-FD-A5BS \| \| TCGA-FD-A5BT \| \| TCGA-FD-A5BU \| \| TCGA-FD-A5BX \| \| TCGA-FD-A5BY \| \| TCGA-FD-A62S \| \| TCGA-FD-A6TA \| \| TCGA-FD-A6TB \| \| TCGA-FD-A6TC \| \| TCGA-FD-A6TD \| \| TCGA-FD-A6TE \| \| TCGA-FD-A6TH \| \| TCGA-FD-A6TI \| \| TCGA-FD-A6TK \| \| TCGA-FJ-A3ZF \| \| TCGA-FJ-A871 \| \| TCGA-FT-A3EE \| \| TCGA-FT-A61P \| \| TCGA-G2-A2EF \| \| TCGA-G2-A2EL \| \| TCGA-G2-A3VY \| \| TCGA-G2-AA3B \| \| TCGA-G2-AA3C \| \| TCGA-G2-AA3F \| \| TCGA-GC-A3BM \| \| TCGA-GC-A3I6 \| \| TCGA-GC-A3OO \| \| TCGA-GC-A3RD \| \| TCGA-GC-A3WC \| \| TCGA-GC-A3YS \| \| TCGA-GC-A6I1 \| \| TCGA-GC-A6I3 \| \| TCGA-GD-A2C5 \| \| TCGA-GD-A3OP \| \| TCGA-GD-A3OQ \| \| TCGA-GU-A763 \| \| TCGA-GU-A764 \| \| TCGA-GU-A766 \| \| TCGA-GU-A767 \| \| TCGA-GU-AATO \| \| TCGA-GU-AATP \| \| TCGA-GV-A3JV \| \| TCGA-GV-A3JW \| \| TCGA-GV-A3JX \| \| TCGA-GV-A3JZ \| \| TCGA-GV-A40G \| \| TCGA-GV-A6ZA \| \| TCGA-H4-A2HO \| \| TCGA-H4-A2HQ \| \| TCGA-HQ-A2OE \| \| TCGA-HQ-A2OF \| \| TCGA-HQ-A5ND \| \| TCGA-K4-A3WS \| \| TCGA-K4-A3WU \| \| TCGA-K4-A3WV \| \| TCGA-K4-A54R \| \| TCGA-K4-A5RH \| \| TCGA-K4-A83P \| \| TCGA-KQ-A41O \| \| TCGA-KQ-A41Q \| \| TCGA-R3-A69X \| \| TCGA-S5-A6DX \| \| TCGA-UY-A78L \| \| TCGA-UY-A78M \| \| TCGA-UY-A78P \| \| TCGA-UY-A8OD \| \| TCGA-UY-A9PA \| \| TCGA-UY-A9PF \| \| TCGA-XF-A8HB \| \| TCGA-XF-A8HC \| \| TCGA-XF-A8HD \| \| TCGA-XF-A8HE \| \| TCGA-XF-A8HG \| \| TCGA-XF-A8HI \| \| TCGA-XF-A9SH \| \| TCGA-XF-A9SL \| \| TCGA-XF-A9SP \| \| TCGA-XF-A9ST \| \| TCGA-XF-A9SV \| \| TCGA-XF-A9SW \| \| TCGA-XF-A9T0 \| \| TCGA-XF-A9T2 \| \| TCGA-XF-A9T4 \| \| TCGA-XF-A9T6 \| \| TCGA-XF-A9T8 \| \| TCGA-XF-AAME \| \| TCGA-XF-AAML \| \| TCGA-XF-AAMR \| \| TCGA-XF-AAMT \| \| TCGA-XF-AAMW \| \| TCGA-XF-AAMX \| \| TCGA-XF-AAMZ \| \| TCGA-XF-AAN2 \| \| TCGA-XF-AAN3 \| \| TCGA-XF-AAN4 \| \| TCGA-XF-AAN7 \| \| TCGA-YC-A9TC \| \| TCGA-YF-AA3L \| \| TCGA-YF-AA3M \| \| TCGA-ZF-A9R1 \| \| TCGA-ZF-A9R3 \| \| TCGA-ZF-A9R4 \| \| TCGA-ZF-A9R7 \| \| TCGA-ZF-A9RC \| \| TCGA-ZF-A9RE \| \| TCGA-ZF-A9RL \| \| TCGA-ZF-A9RN \| \| TCGA-ZF-AA4N \| \| TCGA-ZF-AA4U \| \| TCGA-ZF-AA4V \| \| TCGA-ZF-AA4X \| \| TCGA-ZF-AA51 \| \| TCGA-ZF-AA54 \| \| TCGA-ZF-AA5H \| \| TCGA-ZF-AA5P \| | \| 7.906849315 \| \| --- \| \| 6.443835616 \| \| 0.695890411 \| \| 1.356164384 \| \| 1.402739726 \| \| 1.430136986 \| \| 2.915068493 \| \| 4.169863014 \| \| 0.052054795 \| \| 3.479452055 \| \| 5.545205479 \| \| 2.819178082 \| \| 3.339726027 \| \| 0.610958904 \| \| 0.221917808 \| \| 0.561643836 \| \| 1.032876712 \| \| 0.547945205 \| \| 2.178082192 \| \| 1.624657534 \| \| 1.24109589 \| \| 1.246575342 \| \| 0.421917808 \| \| 0.687671233 \| \| 1.347945205 \| \| 2.161643836 \| \| 2.391780822 \| \| 3.035616438 \| \| 2.367123288 \| \| 0.747945205 \| \| 0.161643836 \| \| 1.917808219 \| \| 1.164383562 \| \| 1.049315068 \| \| 1.010958904 \| \| 1.090410959 \| \| 1.038356164 \| \| 1.008219178 \| \| 1.021917808 \| \| 0.945205479 \| \| 0.945205479 \| \| 1.547945205 \| \| 1.090410959 \| \| 0.408219178 \| \| 1.260273973 \| \| 2.482191781 \| \| 0.153424658 \| \| 1.821917808 \| \| 5.534246575 \| \| 1.583561644 \| \| 9.369863014 \| \| 1.345205479 \| \| 1.468493151 \| \| 3.890410959 \| \| 1.495890411 \| \| 10.50684932 \| \| 1.131506849 \| \| 0.684931507 \| \| 1.476712329 \| \| 0.805479452 \| \| 1.734246575 \| \| 0.879452055 \| \| 1.498630137 \| \| 5.61369863 \| \| 3.054794521 \| \| 3.18630137 \| \| 1.252054795 \| \| 1.131506849 \| \| 13.8109589 \| \| 1.136986301 \| \| 3.632876712 \| \| 3.235616438 \| \| 1.391780822 \| \| 0.054794521 \| \| 2.246575342 \| \| 0.353424658 \| \| 1.139726027 \| \| 1.17260274 \| \| 0.745205479 \| \| 0.334246575 \| \| 2.024657534 \| \| 1.498630137 \| \| 0.460273973 \| \| 2.145205479 \| \| 1.649315068 \| \| 1.071232877 \| \| 1.742465753 \| \| 4.490410959 \| \| 0.898630137 \| \| 1.610958904 \| \| 0.473972603 \| \| 0.687671233 \| \| 1.112328767 \| \| 5.238356164 \| \| 1.567123288 \| \| 0.512328767 \| \| 1.057534247 \| \| 1.030136986 \| \| 0.35890411 \| \| 0.805479452 \| \| 0.904109589 \| \| 1.435616438 \| \| 0.745205479 \| \| 0.271232877 \| \| 0.923287671 \| \| 5.161643836 \| \| 2.243835616 \| \| 1.468493151 \| \| 5.501369863 \| \| 0.578082192 \| \| 2.446575342 \| \| 1.783561644 \| \| 1.726027397 \| \| 1.317808219 \| \| 1.17260274 \| \| 1.479452055 \| \| 2.076712329 \| \| 0.002739726 \| \| 0.002739726 \| \| 2.224657534 \| \| 0.175342466 \| \| 0.260273973 \| \| 2.731506849 \| \| 1.671232877 \| \| 1.315068493 \| \| 0.394520548 \| \| 0.887671233 \| \| 2.747945205 \| \| 1.189041096 \| \| 1.778082192 \| \| 1.591780822 \| \| 1.652054795 \| \| 1.589041096 \| \| 1.893150685 \| \| 0.126027397 \| \| 1.616438356 \| \| 3.216438356 \| \| 5.334246575 \| \| 0.750684932 \| \| 2.084931507 \| \| 0.287671233 \| \| 1.769863014 \| \| 2.306849315 \| \| 0.756164384 \| \| 1.356164384 \| \| 4.21369863 \| \| 0.989041096 \| \| 1.18630137 \| \| 0.153424658 \| \| 3.087671233 \| \| 1.890410959 \| \| 6.520547945 \| \| 9.402739726 \| \| 2.936986301 \| \| 0.320547945 \| \| 3.753424658 \| \| 0.547945205 \| \| 8.120547945 \| \| 10.45753425 \| \| 1.279452055 \| \| 1.490410959 \| \| 5.4 \| \| 5.534246575 \| \| 1.243835616 \| \| 0.350684932 \| \| 1.063013699 \| \| 0.991780822 \| \| 2.189041096 \| \| 1.575342466 \| \| 1.356164384 \| \| 0.175342466 \| \| 1.145205479 \| \| 7.747945205 \| \| 0.635616438 \| \| 7.643835616 \| \| 0.246575342 \| \| 0.693150685 \| \| 0.564383562 \| \| 3.693150685 \| \| 5.120547945 \| \| 7.191780822 \| \| 2.254794521 \| \| 1.547945205 \| \| 0.054794521 \| \| 0.997260274 \| \| 1.136986301 \| \| 2.117808219 \| \| 2.6 \| \| 2.523287671 \| \| 1.821917808 \| \| 7.857534247 \| \| 0.290410959 \| \| 7.405479452 \| \| 1.684931507 \| \| 0.24109589 \| \| 0.717808219 \| \| 4.947945205 \| \| 5.6 \| \| 4.695890411 \| \| 1.616438356 \| \| 2.457534247 \| \| 1.019178082 \| | \| alive \| \| --- \| \| alive \| \| alive \| \| alive \| \| alive \| \| alive \| \| alive \| \| alive \| \| alive \| \| alive \| \| alive \| \| alive \| \| alive \| \| alive \| \| alive \| \| alive \| \| alive \| \| alive \| \| alive \| \| alive \| \| alive \| \| alive \| \| alive \| \| alive \| \| alive \| \| alive \| \| alive \| \| alive \| \| alive \| \| alive \| \| alive \| \| alive \| \| alive \| \| alive \| \| alive \| \| alive \| \| alive \| \| alive \| \| alive \| \| alive \| \| alive \| \| alive \| \| alive \| \| alive \| \| alive \| \| alive \| \| alive \| \| alive \| \| alive \| \| alive \| \| alive \| \| alive \| \| alive \| \| alive \| \| alive \| \| alive \| \| alive \| \| alive \| \| alive \| \| alive \| \| alive \| \| alive \| \| alive \| \| alive \| \| alive \| \| alive \| \| alive \| \| alive \| \| alive \| \| alive \| \| alive \| \| alive \| \| alive \| \| alive \| \| alive \| \| alive \| \| alive \| \| alive \| \| alive \| \| alive \| \| alive \| \| alive \| \| alive \| \| alive \| \| alive \| \| alive \| \| alive \| \| alive \| \| alive \| \| alive \| \| alive \| \| alive \| \| alive \| \| alive \| \| alive \| \| alive \| \| alive \| \| alive \| \| alive \| \| alive \| \| alive \| \| alive \| \| alive \| \| alive \| \| alive \| \| alive \| \| alive \| \| alive \| \| alive \| \| alive \| \| alive \| \| alive \| \| alive \| \| alive \| \| alive \| \| alive \| \| alive \| \| alive \| \| dead \| \| dead \| \| dead \| \| dead \| \| dead \| \| dead \| \| dead \| \| dead \| \| dead \| \| dead \| \| dead \| \| dead \| \| dead \| \| dead \| \| dead \| \| dead \| \| dead \| \| dead \| \| dead \| \| dead \| \| dead \| \| dead \| \| dead \| \| dead \| \| dead \| \| dead \| \| dead \| \| dead \| \| dead \| \| dead \| \| dead \| \| dead \| \| dead \| \| dead \| \| dead \| \| dead \| \| dead \| \| dead \| \| dead \| \| dead \| \| dead \| \| dead \| \| dead \| \| dead \| \| dead \| \| dead \| \| dead \| \| dead \| \| dead \| \| dead \| \| dead \| \| dead \| \| dead \| \| dead \| \| dead \| \| dead \| \| dead \| \| dead \| \| dead \| \| dead \| \| dead \| \| dead \| \| dead \| \| dead \| \| dead \| \| dead \| \| dead \| \| dead \| \| dead \| \| dead \| \| dead \| \| dead \| \| dead \| \| dead \| \| dead \| \| dead \| \| dead \| \| dead \| \| dead \| \| dead \| \| dead \| \| dead \| \| dead \| \| dead \| |
