# Supplementary material for: Identification of anoikis-related molecular patterns and the novel risk model to predict prognosis, tumor microenvironment infiltration and immunotherapy response in bladder cancer
Source: Front Immunol. 2024 Nov 27;15:1491808. doi: 10.3389/fimmu.2024.1491808 (PMC11631915; doi:10.3389/fimmu.2024.1491808)
Supplement: Supplementary file 18 [file Table11.docx]

**Table S11. BLCA patients in the testing set.**

| Sample | Survival time | Survival status |
| --- | --- | --- |
| \| TCGA-2F-A9KO \| \| --- \| \| TCGA-2F-A9KP \| \| TCGA-2F-A9KR \| \| TCGA-4Z-AA7N \| \| TCGA-4Z-AA7Q \| \| TCGA-4Z-AA7W \| \| TCGA-4Z-AA82 \| \| TCGA-4Z-AA84 \| \| TCGA-4Z-AA86 \| \| TCGA-4Z-AA87 \| \| TCGA-5N-A9KI \| \| TCGA-5N-A9KM \| \| TCGA-BT-A0YX \| \| TCGA-BT-A20J \| \| TCGA-BT-A20O \| \| TCGA-BT-A20P \| \| TCGA-BT-A20R \| \| TCGA-BT-A20W \| \| TCGA-BT-A2LA \| \| TCGA-BT-A2LD \| \| TCGA-BT-A3PH \| \| TCGA-BT-A3PK \| \| TCGA-C4-A0F1 \| \| TCGA-C4-A0F7 \| \| TCGA-CF-A1HR \| \| TCGA-CF-A1HS \| \| TCGA-CF-A3MI \| \| TCGA-CF-A47S \| \| TCGA-CF-A47T \| \| TCGA-CF-A47X \| \| TCGA-CF-A5U8 \| \| TCGA-CF-A5UA \| \| TCGA-CF-A7I0 \| \| TCGA-CF-A9FF \| \| TCGA-CU-A0YN \| \| TCGA-CU-A3KJ \| \| TCGA-CU-A3QU \| \| TCGA-CU-A72E \| \| TCGA-DK-A1A5 \| \| TCGA-DK-A1A7 \| \| TCGA-DK-A1AB \| \| TCGA-DK-A1AC \| \| TCGA-DK-A1AG \| \| TCGA-DK-A2I2 \| \| TCGA-DK-A2I6 \| \| TCGA-DK-A3IK \| \| TCGA-DK-A3IM \| \| TCGA-DK-A3IS \| \| TCGA-DK-A3IT \| \| TCGA-DK-A3IU \| \| TCGA-DK-A3WY \| \| TCGA-DK-A3X1 \| \| TCGA-DK-A6AV \| \| TCGA-DK-A6B0 \| \| TCGA-DK-A6B2 \| \| TCGA-DK-A6B5 \| \| TCGA-DK-AA6M \| \| TCGA-DK-AA6S \| \| TCGA-DK-AA6T \| \| TCGA-DK-AA6U \| \| TCGA-DK-AA6W \| \| TCGA-DK-AA6X \| \| TCGA-DK-AA74 \| \| TCGA-DK-AA75 \| \| TCGA-DK-AA76 \| \| TCGA-DK-AA77 \| \| TCGA-E5-A4TZ \| \| TCGA-E7-A3X6 \| \| TCGA-E7-A3Y1 \| \| TCGA-E7-A4IJ \| \| TCGA-E7-A4XJ \| \| TCGA-E7-A541 \| \| TCGA-E7-A5KE \| \| TCGA-E7-A678 \| \| TCGA-E7-A6ME \| \| TCGA-E7-A6MF \| \| TCGA-E7-A7DU \| \| TCGA-E7-A7DV \| \| TCGA-E7-A85H \| \| TCGA-E7-A8O7 \| \| TCGA-E7-A8O8 \| \| TCGA-E7-A97P \| \| TCGA-FD-A3B3 \| \| TCGA-FD-A3B4 \| \| TCGA-FD-A3B6 \| \| TCGA-FD-A3B8 \| \| TCGA-FD-A3N5 \| \| TCGA-FD-A3N6 \| \| TCGA-FD-A3NA \| \| TCGA-FD-A3SL \| \| TCGA-FD-A3SN \| \| TCGA-FD-A3SQ \| \| TCGA-FD-A43N \| \| TCGA-FD-A43P \| \| TCGA-FD-A43S \| \| TCGA-FD-A43X \| \| TCGA-FD-A43Y \| \| TCGA-FD-A5BR \| \| TCGA-FD-A5BV \| \| TCGA-FD-A5BZ \| \| TCGA-FD-A5C0 \| \| TCGA-FD-A5C1 \| \| TCGA-FD-A62N \| \| TCGA-FD-A62O \| \| TCGA-FD-A62P \| \| TCGA-FD-A6TF \| \| TCGA-FD-A6TG \| \| TCGA-FJ-A3Z7 \| \| TCGA-FJ-A3Z9 \| \| TCGA-FJ-A3ZE \| \| TCGA-G2-A2EC \| \| TCGA-G2-A2EJ \| \| TCGA-G2-A2EK \| \| TCGA-G2-A2EO \| \| TCGA-G2-A2ES \| \| TCGA-G2-A3IB \| \| TCGA-G2-A3IE \| \| TCGA-G2-AA3D \| \| TCGA-GC-A3RB \| \| TCGA-GC-A3RC \| \| TCGA-GC-A4ZW \| \| TCGA-GD-A3OS \| \| TCGA-GD-A6C6 \| \| TCGA-GD-A76B \| \| TCGA-GU-A42P \| \| TCGA-GU-A42Q \| \| TCGA-GU-A42R \| \| TCGA-GU-A762 \| \| TCGA-GU-AATQ \| \| TCGA-GV-A3QF \| \| TCGA-GV-A3QH \| \| TCGA-GV-A3QI \| \| TCGA-GV-A3QK \| \| TCGA-GV-A40E \| \| TCGA-HQ-A5NE \| \| TCGA-K4-A4AB \| \| TCGA-K4-A4AC \| \| TCGA-K4-A5RI \| \| TCGA-K4-A5RJ \| \| TCGA-K4-A6FZ \| \| TCGA-K4-A6MB \| \| TCGA-K4-AAQO \| \| TCGA-KQ-A41N \| \| TCGA-KQ-A41P \| \| TCGA-KQ-A41R \| \| TCGA-KQ-A41S \| \| TCGA-LC-A66R \| \| TCGA-LT-A5Z6 \| \| TCGA-LT-A8JT \| \| TCGA-MV-A51V \| \| TCGA-PQ-A6FI \| \| TCGA-PQ-A6FN \| \| TCGA-S5-AA26 \| \| TCGA-SY-A9G0 \| \| TCGA-SY-A9G5 \| \| TCGA-UY-A78K \| \| TCGA-UY-A78N \| \| TCGA-UY-A78O \| \| TCGA-UY-A8OB \| \| TCGA-UY-A8OC \| \| TCGA-UY-A9PB \| \| TCGA-UY-A9PD \| \| TCGA-UY-A9PE \| \| TCGA-UY-A9PH \| \| TCGA-XF-A8HF \| \| TCGA-XF-A8HH \| \| TCGA-XF-A9SI \| \| TCGA-XF-A9SJ \| \| TCGA-XF-A9SK \| \| TCGA-XF-A9SM \| \| TCGA-XF-A9SU \| \| TCGA-XF-A9SX \| \| TCGA-XF-A9SY \| \| TCGA-XF-A9SZ \| \| TCGA-XF-A9T3 \| \| TCGA-XF-A9T5 \| \| TCGA-XF-AAMG \| \| TCGA-XF-AAMH \| \| TCGA-XF-AAMJ \| \| TCGA-XF-AAMQ \| \| TCGA-XF-AAMY \| \| TCGA-XF-AAN0 \| \| TCGA-XF-AAN1 \| \| TCGA-XF-AAN5 \| \| TCGA-XF-AAN8 \| \| TCGA-YC-A89H \| \| TCGA-YC-A8S6 \| \| TCGA-ZF-A9R0 \| \| TCGA-ZF-A9R2 \| \| TCGA-ZF-A9R5 \| \| TCGA-ZF-A9R9 \| \| TCGA-ZF-A9RD \| \| TCGA-ZF-A9RF \| \| TCGA-ZF-A9RM \| \| TCGA-ZF-AA4R \| \| TCGA-ZF-AA4T \| \| TCGA-ZF-AA4W \| \| TCGA-ZF-AA52 \| \| TCGA-ZF-AA53 \| \| TCGA-ZF-AA56 \| \| TCGA-ZF-AA58 \| \| TCGA-ZF-AA5N \| | \| 2.010958904 \| \| --- \| \| 0.997260274 \| \| 8.720547945 \| \| 3.745205479 \| \| 1.397260274 \| \| 2.301369863 \| \| 4.263013699 \| \| 1.260273973 \| \| 0.852054795 \| \| 3.983561644 \| \| 0.208219178 \| \| 1.452054795 \| \| 1.095890411 \| \| 1.58630137 \| \| 1.01369863 \| \| 1.490410959 \| \| 0.421917808 \| \| 0.695890411 \| \| 1.430136986 \| \| 1.706849315 \| \| 0.389041096 \| \| 0.830136986 \| \| 0.243835616 \| \| 0.169863014 \| \| 1.065753425 \| \| 1.046575342 \| \| 1.01369863 \| \| 0.912328767 \| \| 1.054794521 \| \| 1.052054795 \| \| 1.093150685 \| \| 1 \| \| 1.008219178 \| \| 0.989041096 \| \| 1.076712329 \| \| 1.539726027 \| \| 0.432876712 \| \| 1.131506849 \| \| 0.178082192 \| \| 1.534246575 \| \| 1.391780822 \| \| 10.90684932 \| \| 1.301369863 \| \| 0.649315068 \| \| 7.276712329 \| \| 0.4 \| \| 0.679452055 \| \| 4.189041096 \| \| 1.775342466 \| \| 1.934246575 \| \| 13.60821918 \| \| 5.504109589 \| \| 5.347945205 \| \| 6.383561644 \| \| 1.306849315 \| \| 4.224657534 \| \| 4.334246575 \| \| 13.83561644 \| \| 1.567123288 \| \| 1.583561644 \| \| 1.136986301 \| \| 1.279452055 \| \| 4.679452055 \| \| 0.931506849 \| \| 1.002739726 \| \| 1.693150685 \| \| 1.279452055 \| \| 2.476712329 \| \| 0.446575342 \| \| 1.846575342 \| \| 0.18630137 \| \| 2.131506849 \| \| 0.046575342 \| \| 2.18630137 \| \| 2.002739726 \| \| 2.054794521 \| \| 0.076712329 \| \| 0.101369863 \| \| 1.079452055 \| \| 1.276712329 \| \| 0.035616438 \| \| 1.197260274 \| \| 2.668493151 \| \| 1.397260274 \| \| 2.753424658 \| \| 1.052054795 \| \| 1.876712329 \| \| 2.331506849 \| \| 5.054794521 \| \| 1.950684932 \| \| 2.430136986 \| \| 3.898630137 \| \| 1.915068493 \| \| 2.279452055 \| \| 1.246575342 \| \| 0.301369863 \| \| 1.298630137 \| \| 2.22739726 \| \| 0.446575342 \| \| 2.287671233 \| \| 1.506849315 \| \| 4.909589041 \| \| 0.224657534 \| \| 0.591780822 \| \| 0.523287671 \| \| 0.189041096 \| \| 0.254794521 \| \| 2.589041096 \| \| 1.054794521 \| \| 0.887671233 \| \| 1.906849315 \| \| 4 \| \| 1.328767123 \| \| 4.942465753 \| \| 2.750684932 \| \| 0.602739726 \| \| 1.676712329 \| \| 5.860273973 \| \| 1.594520548 \| \| 1.326027397 \| \| 0.04109589 \| \| 1.747945205 \| \| 0.183561644 \| \| 0.61369863 \| \| 0.909589041 \| \| 0.942465753 \| \| 1.580821918 \| \| 0.635616438 \| \| 0.583561644 \| \| 1.690410959 \| \| 0.706849315 \| \| 3.04109589 \| \| 2.279452055 \| \| 0.715068493 \| \| 1.01369863 \| \| 0.208219178 \| \| 0.761643836 \| \| 0.975342466 \| \| 1.476712329 \| \| 0.150684932 \| \| 1.284931507 \| \| 0.983561644 \| \| 4.394520548 \| \| 2.997260274 \| \| 3.698630137 \| \| 0.095890411 \| \| 1.276712329 \| \| 1.298630137 \| \| 1.756164384 \| \| 1.123287671 \| \| 1.019178082 \| \| 1.389041096 \| \| 1.378082192 \| \| 2.761643836 \| \| 3.249315068 \| \| 1.468493151 \| \| 7.235616438 \| \| 6.334246575 \| \| 5.778082192 \| \| 0.002739726 \| \| 2.463013699 \| \| 1.484931507 \| \| 0.517808219 \| \| 4.276712329 \| \| 8.093150685 \| \| 0.156164384 \| \| 6.638356164 \| \| 0.268493151 \| \| 1.331506849 \| \| 2.871232877 \| \| 0.498630137 \| \| 1.969863014 \| \| 1.753424658 \| \| 2.353424658 \| \| 0.18630137 \| \| 5.553424658 \| \| 9.216438356 \| \| 0.942465753 \| \| 4.575342466 \| \| 5.964383562 \| \| 8.249315068 \| \| 4.706849315 \| \| 2.578082192 \| \| 6.282191781 \| \| 0.323287671 \| \| 1.569863014 \| \| 0.802739726 \| \| 1.863013699 \| \| 1.75890411 \| \| 2.98630137 \| \| 2.367123288 \| \| 1.117808219 \| \| 5.339726027 \| \| 3.98630137 \| \| 2.838356164 \| \| 1.64109589 \| \| 5.01369863 \| \| 2.950684932 \| \| 4.824657534 \| \| 0.709589041 \| \| 4.517808219 \| \| 0.460273973 \| | \| alive \| \| --- \| \| alive \| \| alive \| \| alive \| \| alive \| \| alive \| \| alive \| \| alive \| \| alive \| \| alive \| \| alive \| \| alive \| \| alive \| \| alive \| \| alive \| \| alive \| \| alive \| \| alive \| \| alive \| \| alive \| \| alive \| \| alive \| \| alive \| \| alive \| \| alive \| \| alive \| \| alive \| \| alive \| \| alive \| \| alive \| \| alive \| \| alive \| \| alive \| \| alive \| \| alive \| \| alive \| \| alive \| \| alive \| \| alive \| \| alive \| \| alive \| \| alive \| \| alive \| \| alive \| \| alive \| \| alive \| \| alive \| \| alive \| \| alive \| \| alive \| \| alive \| \| alive \| \| alive \| \| alive \| \| alive \| \| alive \| \| alive \| \| alive \| \| alive \| \| alive \| \| alive \| \| alive \| \| alive \| \| alive \| \| alive \| \| alive \| \| alive \| \| alive \| \| alive \| \| alive \| \| alive \| \| alive \| \| alive \| \| alive \| \| alive \| \| alive \| \| alive \| \| alive \| \| alive \| \| alive \| \| alive \| \| alive \| \| alive \| \| alive \| \| alive \| \| alive \| \| alive \| \| alive \| \| alive \| \| alive \| \| alive \| \| alive \| \| alive \| \| alive \| \| alive \| \| alive \| \| alive \| \| alive \| \| alive \| \| alive \| \| alive \| \| alive \| \| alive \| \| alive \| \| alive \| \| alive \| \| alive \| \| alive \| \| alive \| \| alive \| \| dead \| \| dead \| \| dead \| \| dead \| \| dead \| \| dead \| \| dead \| \| dead \| \| dead \| \| dead \| \| dead \| \| dead \| \| dead \| \| dead \| \| dead \| \| dead \| \| dead \| \| dead \| \| dead \| \| dead \| \| dead \| \| dead \| \| dead \| \| dead \| \| dead \| \| dead \| \| dead \| \| dead \| \| dead \| \| dead \| \| dead \| \| dead \| \| dead \| \| dead \| \| dead \| \| dead \| \| dead \| \| dead \| \| dead \| \| dead \| \| dead \| \| dead \| \| dead \| \| dead \| \| dead \| \| dead \| \| dead \| \| dead \| \| dead \| \| dead \| \| dead \| \| dead \| \| dead \| \| dead \| \| dead \| \| dead \| \| dead \| \| dead \| \| dead \| \| dead \| \| dead \| \| dead \| \| dead \| \| dead \| \| dead \| \| dead \| \| dead \| \| dead \| \| dead \| \| dead \| \| dead \| \| dead \| \| dead \| \| dead \| \| dead \| \| dead \| \| dead \| \| dead \| \| dead \| \| dead \| \| dead \| \| dead \| \| dead \| \| dead \| \| dead \| \| dead \| \| dead \| \| dead \| \| dead \| \| dead \| \| dead \| \| dead \| |
