# Supplementary material for: Identification of anoikis-related molecular patterns and the novel risk model to predict prognosis, tumor microenvironment infiltration and immunotherapy response in bladder cancer
Source: Front Immunol. 2024 Nov 27;15:1491808. doi: 10.3389/fimmu.2024.1491808 (PMC11631915; doi:10.3389/fimmu.2024.1491808)
Supplement: Supplementary file 19 [file Table12.docx]

**Table S12. The detail comparison results of correlation between tumor-infiltrating immune cells and riskScore in bladder cancer (BLCA).**

| Symbol | Correction | P value |
| --- | --- | --- |
| \| B cell_TIMER \| \| --- \| \| T cell CD4+_TIMER \| \| T cell CD8+_TIMER \| \| Neutrophil_TIMER \| \| Macrophage_TIMER \| \| Myeloid dendritic cell_TIMER \| \| B cell memory_CIBERSORT \| \| B cell plasma_CIBERSORT \| \| T cell CD8+_CIBERSORT \| \| T cell CD4+ naive_CIBERSORT \| \| T cell CD4+ memory resting_CIBERSORT \| \| T cell CD4+ memory activated_CIBERSORT \| \| T cell follicular helper_CIBERSORT \| \| T cell regulatory (Tregs)_CIBERSORT \| \| Monocyte_CIBERSORT \| \| Macrophage M0_CIBERSORT \| \| Macrophage M1_CIBERSORT \| \| Macrophage M2_CIBERSORT \| \| Myeloid dendritic cell activated_CIBERSORT \| \| Mast cell resting_CIBERSORT \| \| Neutrophil_CIBERSORT \| \| B cell naive_CIBERSORT-ABS \| \| T cell CD8+_CIBERSORT-ABS \| \| T cell CD4+ naive_CIBERSORT-ABS \| \| T cell CD4+ memory resting_CIBERSORT-ABS \| \| T cell CD4+ memory activated_CIBERSORT-ABS \| \| T cell follicular helper_CIBERSORT-ABS \| \| NK cell activated_CIBERSORT-ABS \| \| Macrophage M0_CIBERSORT-ABS \| \| Macrophage M1_CIBERSORT-ABS \| \| Macrophage M2_CIBERSORT-ABS \| \| Myeloid dendritic cell resting_CIBERSORT-ABS \| \| Myeloid dendritic cell activated_CIBERSORT-ABS \| \| Mast cell resting_CIBERSORT-ABS \| \| Neutrophil_CIBERSORT-ABS \| \| B cell_QUANTISEQ \| \| Macrophage M1_QUANTISEQ \| \| Macrophage M2_QUANTISEQ \| \| Monocyte_QUANTISEQ \| \| T cell CD8+_QUANTISEQ \| \| T cell regulatory (Tregs)_QUANTISEQ \| \| Myeloid dendritic cell_QUANTISEQ \| \| uncharacterized cell_QUANTISEQ \| \| T cell_MCPCOUNTER \| \| T cell CD8+_MCPCOUNTER \| \| cytotoxicity score_MCPCOUNTER \| \| NK cell_MCPCOUNTER \| \| B cell_MCPCOUNTER \| \| Monocyte_MCPCOUNTER \| \| Macrophage/Monocyte_MCPCOUNTER \| \| Myeloid dendritic cell_MCPCOUNTER \| \| Endothelial cell_MCPCOUNTER \| \| Cancer associated fibroblast_MCPCOUNTER \| \| Myeloid dendritic cell activated_XCELL \| \| T cell CD4+ naive_XCELL \| \| T cell CD4+ central memory_XCELL \| \| T cell CD4+ effector memory_XCELL \| \| T cell CD8+ naive_XCELL \| \| T cell CD8+_XCELL \| \| Class-switched memory B cell_XCELL \| \| Common lymphoid progenitor_XCELL \| \| Common myeloid progenitor_XCELL \| \| Myeloid dendritic cell_XCELL \| \| Eosinophil_XCELL \| \| Cancer associated fibroblast_XCELL \| \| Granulocyte-monocyte progenitor_XCELL \| \| Macrophage_XCELL \| \| Macrophage M1_XCELL \| \| Macrophage M2_XCELL \| \| Mast cell_XCELL \| \| Monocyte_XCELL \| \| Plasmacytoid dendritic cell_XCELL \| \| B cell plasma_XCELL \| \| T cell CD4+ Th2_XCELL \| \| immune score_XCELL \| \| stroma score_XCELL \| \| microenvironment score_XCELL \| \| Cancer associated fibroblast_EPIC \| \| T cell CD4+_EPIC \| \| T cell CD8+_EPIC \| \| Macrophage_EPIC \| \| NK cell_EPIC \| \| uncharacterized cell_EPIC \| | \| -0.111428478 \| \| --- \| \| 0.186800221 \| \| 0.508803072 \| \| 0.497779306 \| \| 0.462476087 \| \| 0.581960926 \| \| -0.147035038 \| \| -0.243005082 \| \| -0.18166465 \| \| -0.208208371 \| \| 0.102096961 \| \| 0.177437688 \| \| -0.254390305 \| \| -0.257218945 \| \| -0.110084233 \| \| 0.306524327 \| \| 0.27120817 \| \| 0.296573385 \| \| -0.276738094 \| \| 0.157211024 \| \| 0.153595614 \| \| 0.117894759 \| \| 0.151552711 \| \| -0.199003863 \| \| 0.241574548 \| \| 0.186327373 \| \| 0.175151973 \| \| 0.257545524 \| \| 0.378498875 \| \| 0.334572216 \| \| 0.470434269 \| \| 0.119106548 \| \| -0.171489351 \| \| 0.241518366 \| \| 0.194500762 \| \| 0.144316259 \| \| 0.525420648 \| \| 0.294577465 \| \| 0.106958582 \| \| 0.191597723 \| \| 0.33673046 \| \| -0.166892381 \| \| -0.35411695 \| \| -0.119762944 \| \| 0.185451648 \| \| 0.342345136 \| \| 0.312872925 \| \| 0.118769114 \| \| 0.475325415 \| \| 0.475325415 \| \| 0.330520728 \| \| 0.271851288 \| \| 0.557732845 \| \| 0.304620215 \| \| -0.262496477 \| \| -0.337949109 \| \| -0.157748771 \| \| -0.234013867 \| \| -0.213116828 \| \| -0.164319403 \| \| 0.139535218 \| \| 0.14244018 \| \| 0.230341499 \| \| -0.246726589 \| \| 0.303477891 \| \| 0.284696946 \| \| 0.381627499 \| \| 0.365708877 \| \| 0.267142565 \| \| 0.216491597 \| \| 0.315467154 \| \| 0.20383434 \| \| -0.209544712 \| \| 0.513688351 \| \| 0.236559504 \| \| 0.256380891 \| \| 0.311135497 \| \| 0.477876688 \| \| -0.313012691 \| \| -0.12545458 \| \| 0.409302436 \| \| 0.289896186 \| \| -0.272166853 \| | \| 0.025108836 \| \| --- \| \| 0.000159178 \| \| 5.46E-28 \| \| 0 \| \| 8.38E-23 \| \| 0 \| \| 0.003053234 \| \| 7.67E-07 \| \| 0.000241898 \| \| 2.46E-05 \| \| 0.040254516 \| \| 0.000338526 \| \| 2.19E-07 \| \| 1.58E-07 \| \| 0.026929497 \| \| 3.09E-10 \| \| 3.05E-08 \| \| 1.20E-09 \| \| 1.55E-08 \| \| 0.001525204 \| \| 0.001961112 \| \| 0.017759928 \| \| 0.002255154 \| \| 5.63E-05 \| \| 8.95E-07 \| \| 0.000165509 \| \| 0.000404727 \| \| 1.53E-07 \| \| 3.29E-15 \| \| 5.06E-12 \| \| 1.22E-23 \| \| 0.016614658 \| \| 0.000536391 \| \| 9.00E-07 \| \| 8.32E-05 \| \| 0.003649538 \| \| 4.74E-30 \| \| 1.57E-09 \| \| 0.031606762 \| \| 0.000106584 \| \| 3.62E-12 \| \| 0.000757842 \| \| 3.04E-13 \| \| 0.016066374 \| \| 0.000177861 \| \| 2.04E-12 \| \| 1.59E-10 \| \| 0.016971801 \| \| 0 \| \| 0 \| \| 1.24E-11 \| \| 3.21E-08 \| \| 0 \| \| 4.03E-10 \| \| 8.61E-08 \| \| 2.99E-12 \| \| 0.001468564 \| \| 1.98E-06 \| \| 1.56E-05 \| \| 0.000916081 \| \| 0.004958913 \| \| 0.004120549 \| \| 2.89E-06 \| \| 5.12E-07 \| \| 4.71E-10 \| \| 5.69E-09 \| \| 1.87E-15 \| \| 3.14E-14 \| \| 4.97E-08 \| \| 1.13E-05 \| \| 8.72E-11 \| \| 3.66E-05 \| \| 2.18E-05 \| \| 1.39E-28 \| \| 1.63E-06 \| \| 1.74E-07 \| \| 2.03E-10 \| \| 0 \| \| 1.56E-10 \| \| 0.011652672 \| \| 0 \| \| 3.44E-09 \| \| 3.09E-08 \| |
